# Predictability of infectious disease outbreak severity: Chikungunya as a case study

**DOI:** 10.1126/sciadv.adt5419

**Published:** 2025-10-03

**Authors:** Alexander D. Meyer, Sandra Mendoza Guerrero, Natalie E. Dean, Kathryn B. Anderson, Steven T. Stoddard, T. Alex Perkins

**Affiliations:** ^1^ Department of Biological Sciences, University of Notre Dame, Notre Dame, IN 46556, USA.; ^2^ Clinical Strategy, Bavarian Nordic Inc., Durham, NC 27703, USA.; ^3^ Department of Biostatistics and Bioinformatics, Rollins School of Public Health, Emory University, Atlanta, GA 30322, USA.; ^4^ Department of Microbiology and Immunology, The State University of New York (SUNY) Upstate Medical University, Syracuse, NY 13210, USA.

## Abstract

A single pathogen can cause outbreaks of varying size and duration in different populations. Anticipating severe outbreaks would facilitate public health preparedness, but the extent to which this is possible is unclear. We conducted a data-driven investigation into the predictability of outbreak severity, using chikungunya virus (CHIKV) as a case study. For mosquito-transmitted viruses like CHIKV, the potential for severe outbreaks is often assessed using climate-based estimates of the basic reproduction number, $R_{0}$ . We derived a large set of $R_{0}$ estimates for CHIKV by fitting a mechanistic model to data from 86 chikungunya outbreaks. These $R_{0}$ estimates were weakly predicted by climatic and other factors. Among deterministic drivers of outbreak severity, the contribution of $R_{0}$ was comparable to that of generation interval length, transmission distance, and population network structure. While aspects of chikungunya outbreak severity are predictable, innovative approaches are needed that look beyond the impacts of climate on $R_{0}$.

## Teaser

Predicting outbreak severity may require looking beyond $R_{0}$ , especially for mosquito-borne pathogens like chikungunya virus.

## INTRODUCTION

The risk of a human population experiencing an outbreak of a non-endemic disease has two components. First is the risk that the disease-causing pathogen is introduced into the population. Introductions occur randomly through various mechanisms (e.g., global travel or spillover from animal populations), so predicting their timing with certainty is usually difficult or impossible (*1*–*4*). The second component is the risk that the introduced pathogen is transmitted through the population on a large scale. Unlike the risk of the introduction itself, the risk and dynamics of subsequent transmission has been extensively studied. According to classic mathematical results, the probability that a pathogen introduction causes an outbreak is determined by the fraction of the population with preexisting immunity to infection, $p_{\text{immune}}$ , and the basic reproduction number of the pathogen in the population, $R_{0}$ . When the effective reproduction number

$$R_{e}=\left(1-p_{\text{immune}}\right)R_{0}$$ (1)

is greater than one, an introduced infection may lead to an outbreak. Provided that certain assumptions hold true, $R_{0}$ and $R_{e}$ can be used to predict several aspects of that outbreak’s dynamics, such as size, duration, peak incidence, and the time at which that peak occurs (*5*–*9*).

The ability to predict these outbreak features (together, the outbreak’s “severity”) would help health systems and other agencies prepare efficient, effective interventions before outbreaks begin. Good predictions are especially important for managing pathogens that cause outbreaks of highly variable severity, including Ebola virus (*10*), *Vibrio cholerae* (*11*), severe acute respiratory syndrome coronavirus 2 (*12*), and mosquito-borne viruses such as dengue virus (DENV) and chikungunya virus (CHIKV) (*13*). However, the factors that cause outbreak severity to vary, including differences in pathogen genetics and ecology (*14*), differences between affected human populations and their interactions with the pathogen (*15*), and the stochasticity intrinsic to the transmission process (*16*), are challenging to quantify for previous outbreaks using epidemiological data, let alone for future outbreaks using other data sources. It is also often unclear that the assumptions that enable accurate predictions based on $R_{0}$ or $R_{e}$ are true for the pathogens and populations of interest. Nonetheless, because these quantities combine several epidemiological factors into convenient single-variable summaries of transmission potential, estimating reproduction numbers is a common starting point for identifying where and when a severe outbreak might occur (*17*–*20*).

Reproduction number-based estimates of potential outbreak severity are used particularly often for assessing the risks posed by mosquito-borne viruses, such as DENV, CHIKV, and Zika virus (ZIKV). For these pathogens, $R_{0}$ is partially determined by the ecology and biology of their mosquito vectors, which are, in turn, influenced by temperature and other environmental variables (*17*, *21*). According to many studies, this implies that $R_{0}$ or related quantities can be estimated for different populations using spatial data on mosquito occurrence, pathogen occurrence, and putative environmental drivers of transmission (*21*–*31*). The resulting assessments appear successful at identifying regions where outbreaks have historically occurred, although they cannot predict when or where pathogens are actually introduced (*29*). However, whether these approaches produce accurate estimates of $R_{0}$ has not been tested. Moreover, it is not clear that $R_{0}$ alone explains the variable severity of mosquito-borne viral outbreaks. The assumptions underlying $R_{0}$-based predictions are frequently violated by reality, and outbreak variability could be more strongly driven by stochasticity or factors not captured by $R_{0}$ , such as transmission heterogeneity and population network structure (i.e., the configuration of individuals in space, and how they are connected by social structures and mosquito movements) (*32*–*37*).

We conducted a data-driven analysis of the factors that contribute to variable outbreak severity for mosquito-borne illnesses, using chikungunya as a case study. Our ultimate goal was to test the extent to which the severity of a mosquito-borne disease outbreak can be predicted using information available before the outbreak begins, especially via estimates of $R_{0}$ . To start, we calibrated a previously published stochastic model (*38*) to available data from chikungunya outbreaks in 86 different populations (see section S1.2). This resulted in estimates of $R_{0}$ and other parameters for each outbreak, as well as a toolkit for simulating additional realistic outbreaks. Next, we used machine learning to test how well those outbreak-based estimates of $R_{0}$ could be inferred before an outbreak using climatic, demographic, and biotic predictors. Last, we devised a variance partition to quantify the contributions of $R_{0}$ and other parameters, population network structure, and stochasticity to variability across simulated outbreaks. Together, these complementary analyses establish expectations for the extent to which chikungunya outbreak severity can be anticipated using climatic and other data. More broadly, this study outlines a set of approaches by which variability in other pathogens’ outbreak features may be better understood.

## RESULTS

### Review of published chikungunya outbreak severity data

**Fig. 1.**
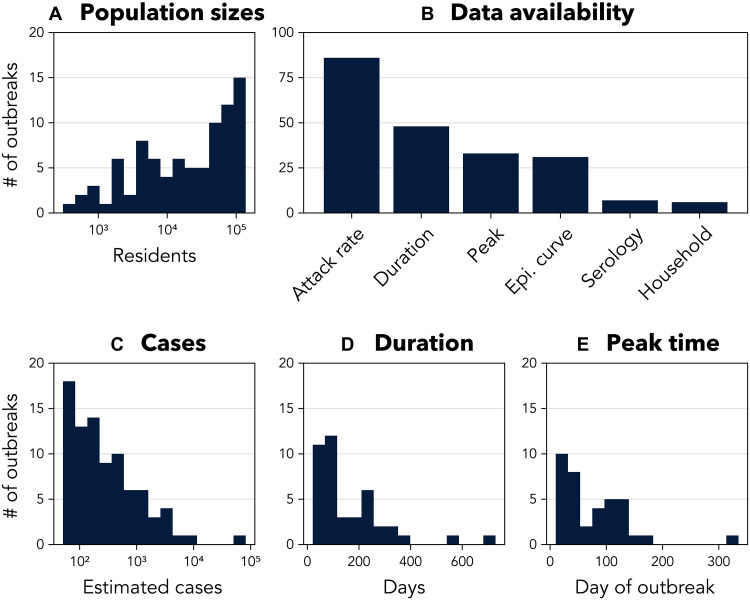
Available data for the 86 chikungunya outbreaks in this study. (**A**) Population sizes (note maximum of 150,000), (**B**) available types of data, (**C**) estimated number of reported chikungunya cases (available for all 86 outbreaks; note: minimum of 50), (**D**) estimated outbreak duration (available for 42 of 86 outbreaks), and (**E**) estimated time from outbreak start to peak incidence (available for 37 of 86 outbreaks). See section S1.2 for data from individual outbreaks.

We reviewed the literature for published records of chikungunya “outbreaks” or clusters of at least 50 documented chikungunya cases within a clearly defined population and time interval (see the “Review of published chikungunya outbreak data” section). To reduce computational costs during our modeling analysis, we excluded outbreaks in populations larger than 150,000 people. This resulted in a sample of 86 outbreaks in distinct populations ranging in size from 300 to 138,000 people (Fig. 1A). These outbreaks varied considerably in severity (size, duration, peak incidence, and peak timing). According to the available data, the outbreaks ranged in size from 51 cases of chikungunya in the Coutos neighborhood of Salvador, Brazil (*39*), to an estimated 82,000 symptomatic infections (including unreported ones) in Port Blair, India (*40*) (Fig. 1C). Attack rates varied from 0.05% (68 of 137,579) in San José, Venezuela (*41*), to 72% (3389 of 4681) in Kalpeni, Lakshadweep Islands, India (*42*). Among the outbreaks of known duration, the shortest lasted only 3 weeks, in Andrott, Lakshadweep Islands, India (*42*), while the longest reportedly lasted for 2 years, in Thung Nari, Thailand (*43*) (Fig. 1D). Because peak incidence was reported using different units by different studies (e.g., cases per day versus cases per month), we were unable to directly compare the peak incidences of different outbreaks. However, all outbreaks with known peak times reached peak incidence in less than 6 months, with the exception of the outbreak in the Chapada district of Riachão do Jacuípe, Brazil (*44*), which peaked after 11 months (Fig. 1E).

The challenge of explaining this observed variability in outbreak severity was compounded by differences in the data available for each outbreak (Fig. 1B). Our outbreak definition (at least 50 cases; see the “Review of published chikungunya outbreak data” section) required that an attack rate be estimable for each outbreak. Only 48 of 86 outbreaks had additional data (e.g., duration) besides attack rates, and only 33 of 86 had estimates of duration, peak incidence, and peak timing. More detailed information, such as serology, was available for a small number of outbreaks. These differences in data availability precluded straightforward comparisons of outbreak features besides attack rate. Rather than comparing these data directly, we used a mathematical model to estimate epidemiological parameters like $R_{0}$ for each outbreak and to simulate aspects of severity that were not measured in real life.

### Outbreak-based estimates of $R_{0}$ and other parameters

The chikungunya outbreak dynamics and data types above vary, but all arose through the same process of mosquito-mediated transmission among humans. To better understand this process, we fitted a realistic model of CHIKV transmission, entitled CHIKSIM (*38*), to data from each of the 86 outbreaks in this study. Model fitting resulted in two useful products for each outbreak: a stochastic simulator for exploring alternate transmission outcomes, and estimates of the epidemiological parameters in Table 1. These parameter estimates were more easily compared across outbreaks than study-specific data types. The parameter estimates from each outbreak also reflected the impacts of transmission drivers that varied spatially among populations at the time of each outbreak, insofar as these were manifest in available data. Here, we briefly summarize the model fitting procedure and the quality of CHIKSIM’s fit to the available data. We then present the estimates of $R_{0}$ and six key epidemiological parameters.

**Table 1.** Epidemiological parameters estimated for each chikungunya outbreak. Note that $R_{0}$ and $p_{\text{detect}}$ were derived from other parameters, rather than being estimated directly. The abbreviation CoV stands for “coefficient of variation,” the ratio of a distribution’s SD to its mean. Prior 95% uncertainty intervals (PUIs) are the 2.5th and 97.5th percentiles of the prior distributions. See the “Model calibration and parameter estimation” section for the prior distributions and section S3.1 for full details. ECSA, East-Central-South African; IOL, Indian Ocean lineage.

Symbol	Meaning	Prior median (95% PUI)	Refs. and notes
*B*	Expected transmissions per CHIKV infection	2.78 (0.99–4.69)	(*13*)
*C*	Heterogeneity (CoV) among households in expected transmissions	1.39 (0.05–7.38)	80/20 rule (*106*)
$p_{\text{immune}}$	Fraction of population with preexisting immunity	25% (1–84%)	(*67*)
$p_{\text{asymp}}$	Probability that CHIKV infection is asymptomatic		
	Asian lineage	47% (17–79%)	(*56*)
	ECSA lineage or IOL	18% (4–44%)	(*56*)
$p_{\text{report}}$	Probability that symptomatic CHIKV infection is reported	50% (9–91%)	Assumed
$p_{\text{detect}}$	Probability that any CHIKV infection is detected	–	$\left(1-p_{\text{asymp}}\right)p_{\text{report}}$
$p_{\text{house}}$	Fraction of transmission occurring within households	50% (9–91%)	(*100*)
*L*	Mean between-house transmission distance (meters)	250 m (10–1330 m)	(*85*, *107*–*110*)
*G*	Mean generation interval length (days)		
	*A. aegypti* at 20°C	22 days (17–30 days)	(*57*, *58*, *111*)
	*A. aegypti* at 30°C	8 days (4–14 days)	(*57*, *58*, *111*)
	*A. albopictus* at 20°C	23 days (17–38 days)	(*57*, *58*, *111*)
	*A. albopictus* at 30°C	8 days (3–16 days)	(*57*, *58*, *111*)
$I_{0}$	CHIKV infections at start of outbreak investigation	4 (1–17)	Assumed
$R_{0}$	Basic reproduction number	–	See the “Model of CHIKV transmission” section
$R_{e}$	Effective reproduction number	–	$\left(1-p_{\text{immune}}\right)R_{0}$

#### Model fit and outbreak summary statistics

The CHIKSIM model was originally published by Meyer *et al.* (*38*), along with a simulation-based Bayesian inference procedure for calibrating it to outbreak datasets. This procedure requires reducing each data set down to a handful (i.e., fewer than 10) of outbreak “summary statistics” that can be extracted from stochastic CHIKSIM simulations. The authors used chikungunya outbreaks in Bangladesh, Cambodia, and Italy as examples (also included in this study), and the summary statistics from each outbreak included total attack rate, outbreak duration, peak incidence and its timing, postoutbreak seropositivity, and the distribution of cases across households. After calibration, CHIKSIM successfully fitted the values of multiple summary statistics simultaneously for each outbreak, indicating that the model represents the CHIKV transmission process reasonably well. See the “Model of CHIKV transmission” and “Model calibration and parameter estimation” sections for more details on CHIKSIM and the inference procedure or (*38*) for complete details.

In this study, we extracted up to seven summary statistics from each of the 86 chikungunya outbreaks, depending on what data were available for each. These summary statistics are summarized in Fig. 1B, and statistics from individual outbreaks are listed in section S1.2. The CHIKSIM model fit most available data quite well (section S3.4) and was especially successful at recreating outbreaks’ sizes, durations, peak incidences, and peak timings (fig. S13). More generally, among all 228 summary statistics fitted across the 86 outbreaks, there were only four statistics whose true values were not contained within CHIKSIM’s 95% posterior prediction intervals (figs. S15 to S21). This included the unusually high attack rate of the outbreak in Kalpeni, India, and the late peak of the outbreak in Chapada, Brazil. Furthermore, CHIKSIM was reasonably successful at producing realistic epidemic curves (fig. S14). Key exceptions included outbreaks that began with unusually high incidence, perhaps indicating a period of undetected CHIKV circulation, or ended abruptly, possibly due to factors not included in the model, such as vector control programs or seasonal forcing. Despite these exceptions, the model fit was encouraging and suggested that CHIKSIM provided a good description of the dynamics underlying each outbreak dataset.

**Fig. 2.**
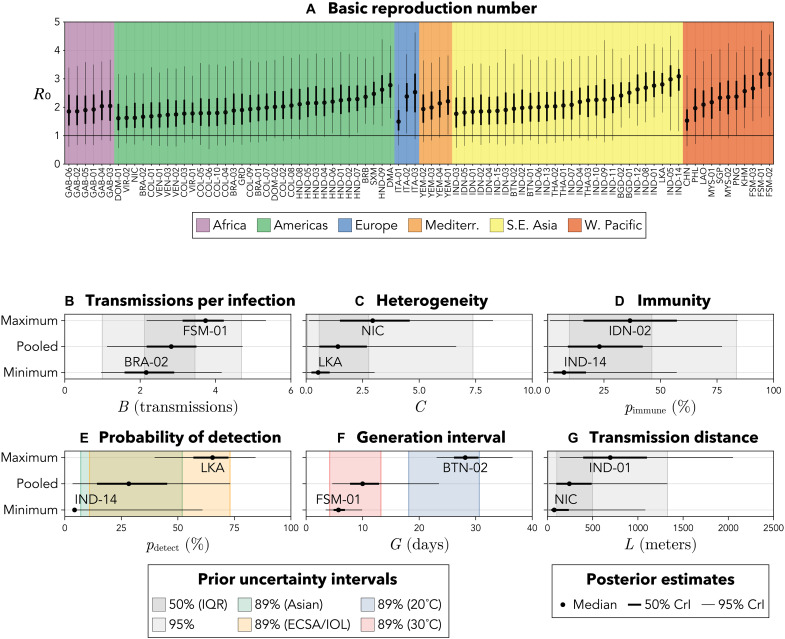
Estimates of key epidemiological parameters for the 86 chikungunya outbreaks. (**A**) Posterior estimates of $R_{0}$ for each outbreak, with a line at the critical value $R_{0}=1$ . Mediterr., Mediterranean; S.E. Asia, South East Asia; W. Pacific, Western Pacific. The remaining panels show prior estimates (shaded regions) and minimum, maximum, and pooled posterior estimates (black dots and bars) for (**B**) expected transmissions per infection, *B*; (**C**) house-to-house transmission heterogeneity, *C*; (**D**) the percentage of each nonnaive population with preexisting immunity, $p_{\text{immune}}$ ; (**E**) the percentage of CHIKV infections detected by outbreak investigations, $p_{\text{detect}}$ ; (**F**) the mean generation interval length, *G*; and (**G**) the mean transmission distance, *L*. See sections S1.2 and S3.2 for outbreaks’ abbreviations and individual parameter estimates, respectively. IQR, interquartile range.

We used simulated data to assess how well each estimated parameter was informed by each outbreak’s available data. For each real outbreak, we used CHIKSIM to generate 100 datasets containing the same types of summary statistics with known parameters (total of 8600 simulated datasets) and then attempted to recover those parameters using our inference procedure. The parameters’ true values were reliably supported by the resulting posterior distributions (fig. S10). However, the parameters presented in Fig. 2 (B to G) were particularly well estimated by these distributions’ medians. Median estimates were generally more accurate for outbreaks for which at least two summary statistics were available, and some parameters were better estimated when certain types of outbreak data were available (figs. S11 and S12). For instance, generation interval length was estimated more accurately for outbreaks with temporal data (e.g., outbreak duration or peak timing). For more details, see section S3.3.

#### *Estimates of* $R_{0}$ *and other key parameters*

*The basic reproduction number,* $R_{0}$. Across all 86 outbreaks, $R_{0}$ for CHIKV had a posterior median estimate of 2.1 [95% credible interval (CrI), 0.8 to 3.9]. Consistent with expectations, posterior mean estimates of $R_{0}$ were greater than one for each individual outbreak (Fig. 2A). We obtained the lowest posterior median estimate of $R_{0}$ , 1.5 (95% CrI, 0.7 to 2.8) for the 2017 chikungunya outbreak in Anzio, Italy [ITA-01; (*45*)], where 182 chikungunya cases were documented among a population of about 54,000 people. The highest estimates of $R_{0}$ came from the chikungunya outbreaks on Ifalik Atoll (3.2; 95% CrI, 1.7 to 4.6) and Fais Island (3.2; 95% CrI, 1.6 to 4.7), Yap Islands, Federated States of Micronesia [respectively, FSM-02 and FSM-01; (*46*)]. These outbreaks were short and intense, resulting in attack rates of 14% over 7 weeks and 44% over 5 weeks, respectively.

Estimates of $R_{0}$ differed across outbreaks of the Asian lineage, East-Central-South African (ECSA) lineage, and Indian Ocean lineage (IOL) of CHIKV. Posterior estimates of $R_{0}$ were highest for outbreaks of the IOL (median, 2.3; 95% CrI, 0.9 to 4.0) and lowest for the ECSA lineage (median, 1.9; 95% CrI, 0.7 to 3.6) (fig. S22A). Estimates of $R_{0}$ also differed across World Health Organization (WHO) regions, with the highest $R_{0}$ estimates for outbreaks in the Western Pacific region (median, 2.4; 95% CrI, 0.9 to 4.3) and the lowest for outbreaks in Africa (median, 1.9; 95% CrI, 0.7 to 3.6). However, this apparent relationship may be due to sampling biases, because both regions were underrepresented in this study compared with South East Asia and the Americas (Fig. 2A).

*Expected transmissions per infection*. The parameter *B* represented the expected number of times mosquitoes transmit CHIKV from a single infected person to other individuals. For a uniform, well-mixed population, *B* is equivalent to $R_{0}$ . Across all outbreaks, *B* had a posterior median of 2.8 and a 95% CrI of 1.1 to 4.7 (Fig. 2B). For each outbreak, the median posterior estimate of *B* was greater than that of $R_{0}$ (Fig. 2A and fig. S8A), illustrating how heterogeneous mixing and transmission can reduce a population’s potential for severe outbreaks.

*Transmission heterogeneity*. In the CHIKSIM model, the expected number of transmissions per infected person varied across households to reflect differences in mosquito abundance and human exposure (e.g., due to the presence of standing water or window screens). The parameter *C* was the coefficient of variation (CoV) across households of individuals’ expected numbers of transmissions (Fig. 2C). Larger values of *C* resulted in greater heterogeneity and, all else equal, shorter outbreaks of chikungunya (see section S2). Across all outbreaks, the parameter *C* had a posterior median of 1.4 and a 95% CrI of 0.04 to 6.6, indicating substantial variability across affected populations. The population with the greatest estimated transmission heterogeneity was District 2 of Managua, Nicaragua [NIC; (*47*)], for which *C* had a posterior median of 2.9 (95% CrI, 0.1 to 8.3).

*Preexisting immunity to CHIKV*. It is generally accepted that no large outbreaks of chikungunya occurred in continental Europe before 2007 or the Americas before 2013 (*48*–*50*). Similarly, the outbreaks that we studied in China (*51*), Laos (*52*), Papua New Guinea (*53*), the Yap Islands (*46*), and Yemen (*54*) were all reported to be the first in those regions. We assumed total susceptibility to CHIKV within in these 47 affected populations. For the outbreaks in the other 39 populations, we estimated the fraction $p_{\text{immune}}$ of individuals with preexisting immunity to CHIKV from previous exposures (Fig. 2D). Across these populations, the median level of immunity before each outbreak was 23% (95% CrI, 1 to 77%). Among outbreaks in this study, we estimated that preexisting immunity was highest before the 2001 chikungunya outbreak in Kali Jaya, Indonesia [IDN-02; (*55*)], with a posterior median of 37% and 95% CrI of 1 to 84%.

*Fraction of CHIKV infections detected*. We separately estimated the probability of a CHIKV infection being asymptomatic, $p_{\text{asymp}}$ , and the probability of a symptomatic CHIKV infection being reported to outbreak investigators as a confirmed chikungunya case, $p_{\text{report}}$ . The fraction of all CHIKV infections detected during an outbreak investigation was

$$p_{\text{detect}}=p_{\text{report}}\left(1-p_{\text{asymp}}\right)$$ (2)

In general, our posterior estimates of the probability of asymptomatic infection adhered closely to the CHIKV lineage–specific priors that we derived from Bustos Carrillo *et al.* (*56*) (fig. S9B). Across all outbreaks, the reporting probability had a posterior median of 46% (95% CrI, 6 to 90%). Combining these estimates, we determined that the posterior probability of a CHIKV infection being detected has a median of 28% (95% CrI, 3 to 73%) (Fig. 2E). We obtained the lowest estimated probability of detection (median, 4%; 95% CrI, 3 to 61%) for the 2006 chikungunya outbreak in Port Blair, India [IND-14; (*40*)]. This estimate was informed by (and comports with) the suggestion by (*40*) that government surveillance may have underestimated the attack rate of this outbreak by nearly 20-fold.

*Mean generation interval length, G*. One of the main objectives of this study was to test the extent to which $R_{0}$ is predicted by climatic and other nonepidemiological data sources. To improve our chance of detecting these relationships, we accounted for effects of temperature and vector species on the generation interval length, *G*, using outbreak-specific priors based on previous research (*57*–*61*). These priors are fully described in section S3.1. We estimated that the generation interval of CHIKV has a posterior median length of 10 days (95% CrI, 5 to 23) across outbreaks (Fig. 2F). The median length was also 10 days for outbreaks vectored by *Aedes aegypti* only or *Aedes albopictus* only. Last, sorting outbreaks by the mean temperatures of their first months (the temperature value used to inform each outbreak’s prior), the generation interval had a posterior median of 9 days (95% CrI, 4 to 15 days) across the 43 warmest populations and 12 days (95% CrI, 6 to 27) across the 43 coolest populations. However, these posterior estimates were heavily informed by our choice of priors; based on experiments using simulated data, the mean generation interval length was especially difficult to estimate for outbreaks for which no temporal data (e.g., duration or peak incidence) were available (figs. S8C and S12G).

*Mean transmission distance*. The parameter *L* represented the expected distance between an infected person and any neighbors to whom they transmitted CHIKV (Fig. 2G). Across all outbreaks, the posterior median estimate of *L* was 240 m (95% CrI, 10 to 1330 m). We estimated that the mean transmission distance was shortest for the outbreak in Managua, NIC (*47*), with a median of 75 m and a 95% CrI of 10 to 1080 m, and longest for the outbreak in Dakshina Kannada, India [IND-01; (*62*)], with a median of 700 m and a 95% CrI of 140 to 2050 m.

### Predicting $R_{0}$ using nonoutbreak data

For *Aedes*-transmitted viruses like CHIKV, efforts to identify populations at risk for transmission focus on estimates of $R_{0}$ (or related quantities) based on data available before outbreaks occur, such as climatic factors (*22*–*24*, *26*–*31*). We conducted a machine learning experiment to test whether the outbreak-based $R_{0}$ estimates above could be predicted by nonoutbreak data sources. Using 10-fold cross-validation, we compared the out-of-sample predictions of over 60,000 models that regressed our posterior $R_{0}$ estimates against combinations of the predictors in Table 2, which are independent from the outbreak data. We used both linear and nonlinear regression algorithms and included untransformed climatic variables as well as four climate-informed indicators of the potential for *Aedes*-mediated viral transmission. Because overfitting is likely when training a large suite of models, we repeated this experiment on a shuffled dataset, in which $R_{0}$ estimates were permuted across outbreaks to remove real-world trends. This established a baseline for the model fit due to overfitting alone. We also repeated this experiment for three random partitions of the 86 outbreaks into 10 validation folds.

**Table 2.** Nonoutbreak data sources considered as possible predictors for $R_{0}$.

Predictor	Notes	Source
Biotic		
Vector species^*^	Species: *A. aegypti* or *A. albopictus*	(*112*, *113*)
CHIKV lineage^*^	Lineages: Asian, ECSA, or IOL	(*21*)
	Included in all models due to observed posterior differences in *R*_0_	
Climatic		
Temperature	Time points: Annual mean, monthly means 0 to 3 months before outbreak	(*114*)
	Extracted from TerraClimate using longitude and latitude	
Relative humidity	Time points: Annual mean, monthly means 0 to 3 months before outbreak	(*114*)
	Extracted from TerraClimate using longitude and latitude	
Precipitation	Time points: Annual mean, monthly means 0 to 3 months before outbreak	(*114*)
	Extracted from TerraClimate using longitude and latitude	
Eco- and Epidemiological		
Aedes occurrence probability	Long-term monthly means derived from environmental data and mosquito presence-absence data	(*21*)
	Time points: Long-term annual mean, long-term monthly means 0 to 3 months before outbreak	
	Extracted using longitude and latitude	
Relative R0	Model of temperature effects on *Aedes*-borne viral transmission	(*29*)
	Time points: Annual mean, monthly means 0 to 3 months before outbreak	
	Computed using vector species and temperature data	
Index-P	Model of temperature and humidity effects on *Aedes*-borne viral transmission	(*26*)
	Time points: Annual mean, monthly means 0 to 3 months before outbreak	
Dengue virus FOI	Single value derived from dengue incidence and environmental data	(*23*)
	Extracted using longitude and latitude	
Demographic/other		
Population density	Log transformed	(*95*, *97*)
Mean household size^*^		(*95*, *97*)
Mean distance to nearest neighbor	Computed by CHIKSIM population submodel	This study
Subnational human development index	Combines measurements of education, health, and standard of living	(*64*, *115*, *116*)
World Health Organization region	Regions: Africa, Americas, and Europe/Mediterranean (combined because of the small sample size), South East Asia, and Western Pacific	–
	Included in all models due to observed posterior differences in $R_{0}$	

* These predictors’ values were extracted from the publications documenting each outbreak, whenever possible; otherwise, we used the sources listed in the table.

**Fig. 3.**
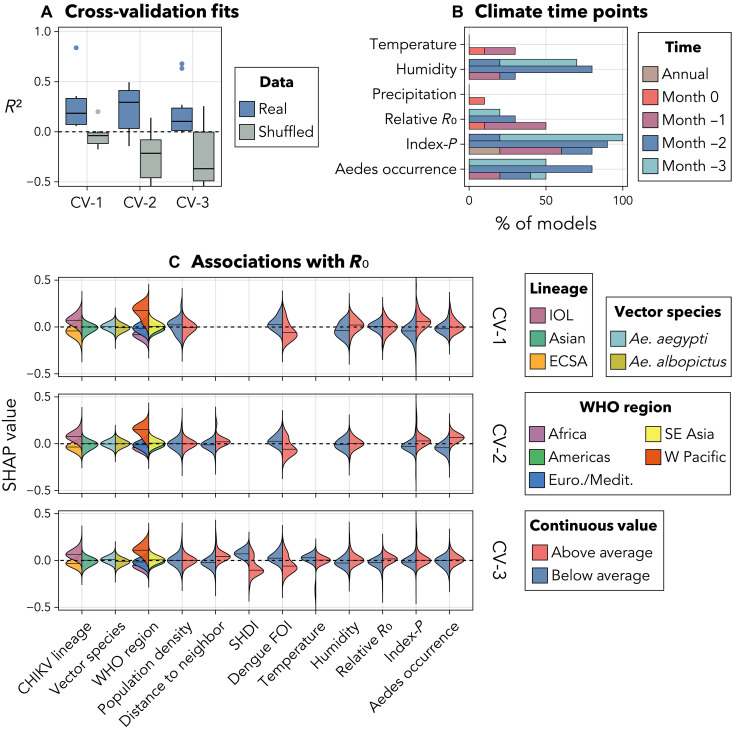
Predicting the outbreak-based $R_{0}$ estimates using nonoutbreak data. (**A**) $R^{2}$ values for the best model in each regression experiment (CV-1, CV-2, and CV-3; real and shuffled data) across validation sets. (**B**) Percentage of the top 10 models in each experiment on real data that used each time point for each climatic variable. “Annual” refers to the annual mean, “Month 0” refers to the month in which each outbreak began, and Months “−1 to −3” refer to the 3 months before each outbreak. (**C**) SHAP values for each predictor used by the 10 best models in each experiment on real data. A SHAP value is computed for each observation of each predictor in each model. A positive (negative) SHAP value indicates that an observation contributed to a larger (smaller) $R_{0}$ estimate in a given model. For example, models generally predicted larger values of $R_{0}$ for outbreaks caused by the IOL lineage but smaller values of $R_{0}$ where the estimated dengue FOI was above average. SHAP values are missing for experiments where a predictor was not used in any of the top 10 models. SHDI, subnational human development index; *Ae. aegypti*, *Aedes aegypti*; *Ae. albopictus*, *Aedes albopictus*. Euro./Medit., Europe/Mediterranean; SE Asia, South East Asia; W Pacific, Western Pacific

This experiment suggested that predictive trends may exist between $R_{0}$ and the predictors in Table 2; however, those trends are noisy and may not apply equally to all sets of outbreaks. On the real dataset, the best models performed heterogeneously across partitions and validation folds (Fig. 3A). The second repetition of cross-validation (experiment CV-2) achieved a median coefficient of determination ( $R^{2}$ ) of 0.29 across validation folds; however, scores on individual folds ranged from *R*^2^ = –0.80 to 0.50. In the third repetition (CV-3), the median $R^{2}$ was considerably lower, at 0.10. Across all 30 validation folds, the median $R^{2}$ on was 0.18. This heterogeneity indicates that the trends learned by the models on each training fold often failed to generalize to the outbreaks in each withheld validation fold. However, model fits on the shuffled dataset were markedly worse, with median $R^{2}=-0.14$ across all validations folds. Thus, while the trends that we detected did not always generalize across outbreaks, it remains likely that $R_{0}$ is associated with some of the predictors in Table 2.

We used Shapley additive explanations [SHAP values; (*63*), see the Fig. 3 caption for details] to quantify the contributions of each predictor to the 10 best models’ estimates of $R_{0}$ in each experiment. Given heterogeneity in the best models’ fits across experiments, the SHAP values suggested only moderately consistent associations between $R_{0}$ and its predictors (Fig. 3C). The differences in $R_{0}$ across CHIKV lineages and WHO regions observed in the previous section were clearest, with larger $R_{0}$ estimates associated with the IOL lineage and outbreaks in the Western Pacific region. In experiment CV-3, $R_{0}$ was positively associated with the distance between households and negatively associated with the subnational human development index, a socioeconomic indicator (*64*), perhaps suggesting greater transmission potential in more rural, less resourced regions. Among the climatic variables, humidity, Index-*P*, and the *Aedes* vector occurrence probability each associated positively with $R_{0}$ in some experiments, but not all three. However, the three experiments were consistent in their use these variables 1 to 3 months before each outbreak, rather than annual means or in each outbreak’s first month (Fig. 3B). Last, we observed a surprising negative association between $R_{0}$ and the estimated force of infection (FOI) of DENV in all three experiments (Fig. 3C; see fig. S22F for confirmation). This association should be interpreted with caution and should not be considered causal, because its biological mechanism is unclear.

### Predictability of chikungunya outbreak severity

In the previous section, we demonstrated the difficulty of estimating $R_{0}$ for CHIKV without epidemiological data. This section complements that analysis with a theoretical question that has practical consequences: If we could estimate $R_{0}$ and other parameters perfectly, then how well would we be able to predict outbreak severity? To answer this question, we applied a variance partition to data from simulated chikungunya outbreaks. The partition attributed variance in the outbreak features that comprise severity (infection attack rate, duration, peak incidence, and peak timing) to four variance sources: differences in affected populations’ epidemiological parameters, parameter uncertainty, differences in affected populations’ network structures, and stochasticity (see the “Partitioning variance in outbreak features” section for details). By “network structure,” we mean each population’s size and the distribution of its residents across households and space, structural features not quantified by any parameters that we considered. To separate the effects of parameters and network structure, we simulated outbreaks for all possible pairs of the 86 affected populations in this study and 86 posterior parameter distributions described in the “Outbreak-based estimates of *R*_0_ and other parameters” section.

**Fig. 4.**
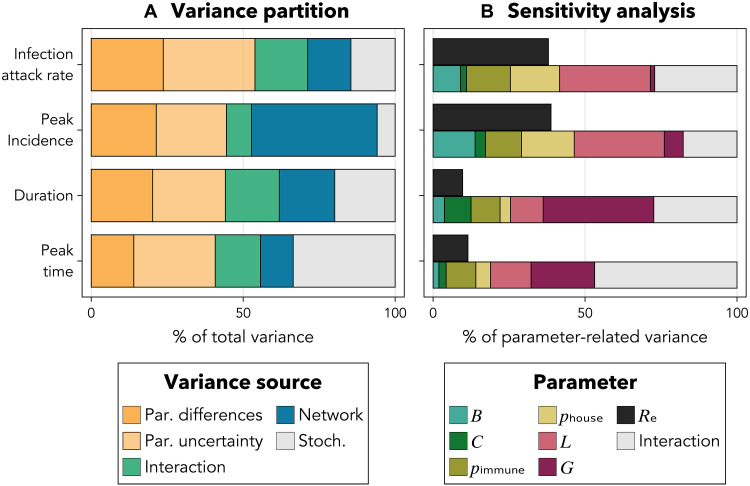
Sources of variance in outbreak severity. (**A**) Variance partition of infection attack rate, peak incidence, outbreak duration, and peak timing across parameters (Par.; differences between and uncertainty within populations), network structure, the interaction of parameters with network structure, and stochasticity (Stoch.). (**B**) First-order sensitivity indices of each outbreak feature’s mean with respect to six model parameters (bottom/multicolored bars) and $R_{e}$ (top/black bars), averaged across affected populations. Sensitivity to $R_{e}$ is shown separately because $R_{e}$ is itself a function of some of the other seven parameters. This sensitivity analysis further partitions the variance attributed to parameter differences and uncertainty in (A). See Table 1 for parameter definitions.

In principle, any variance in the severity of simulated outbreaks that is not attributable to stochasticity must be due to deterministic sources, either parameter values or population network structure. We found that stochasticity accounted for 15% of variance in the CHIKV infection attack rate, 6% of variance in peak weekly incidence, 20% of variance in duration, and 34% of variance in peak timing across simulated outbreaks (Fig. 4A). The remaining 66 to 94% of these features’ variance was due to deterministic sources. Using parameters alone, we can explain 54% of the variance in infection attack rate, 44% in the variance in peak incidence, 44% of the variance in outbreak duration, and 41% of the variance in peak timing. The largest share of the variance due to parameters was explained by parameter uncertainty, while smaller fractions were due to differences between populations and interactions with population with network structure (Fig. 4A). The large contribution of uncertainty reflects the the high variance of most outbreaks’ posterior parameter estimates compared with those parameters’ differences across populations (Fig. 2). In section S5.2, we show that using less uncertain parameter estimates reduces the total variance in outbreak severity but does not change the total fraction of variance explained by parameters. Thus, mean parameter differences and uncertainty are both biologically meaningful sources of variance when attributing variance in outbreak severity to parameters.

Population network structure seems to play an important role in shaping chikungunya outbreak severity, despite being difficult to quantify for real populations. Excluding interactions with parameter values, network structure accounted for 14% of the variance in infection attack rate, 18% of the variance in outbreak duration, 11% of the variance in peak timing, and most notably, 41% of the variance in peak incidence (Fig. 4A). We showed in section S5.3 that, in our model, $R_{0}$ is determined almost entirely by model parameters, and not by network structure. Thus, for chikungunya, classic approaches for predicting outbreak features that rely only on $R_{0}$ and ignore network structure may be misleading. This includes estimates of outbreak risk and attack rate derived from susceptible-infectious-recovered and branching process models that exclude heterogeneity in contacts, transmission, and susceptibility (*35*, *36*, *65*).

Last, we performed a variance-based sensitivity analysis to estimate the fraction of each outbreak feature’s parameter-driven variance due to each individual parameter (*66*). We omitted interactions between multiple parameters due to computational constraints and separately computed the sensitivity of each feature to the effective reproduction number $R_{e}$ , which depends on the other parameters. The outbreak features most sensitive to $R_{e}$ were infection attack rate and peak incidence (Fig. 4B). Approximately 40% of these features’ parameter-related variance was due to $R_{e}$ ; thus, $R_{e}$ explained 40% × 54% (contribution of all parameters together) = 21% of the total variance in infection attack rate and 43% × 44% = 19% of the total variance in peak incidence. The same argument results in $R_{e}$ explaining less than 5% of the total variance in outbreak duration and peak timing. Thus, $R_{e}$ alone may be an inadequate descriptor of transmission potential, both because it excludes effects of network structure and because it does not summarize the effects of all relevant parameters. Additional parameters with similar or greater effects on outbreak severity include the mean transmission distance, *L*, for outbreak magnitude (e.g., attack rate and peak incidence) and the mean generation interval length, *G*, for outbreak timing (e.g., duration and peak time) (Fig. 4B).

## DISCUSSION

Outbreaks of chikungunya and other diseases vary markedly in severity, including size, duration, and the time and magnitude of peak incidence. In this study, we sought to better understand this variability and the extent to which it can be predicted before outbreaks occur. We compiled data from chikungunya outbreaks in 86 different populations around the world. We quantified several aspects of chikungunya epidemiology underlying these outbreaks by estimating nine model-based parameters for each, including the basic reproduction number, $R_{0}$ . We achieved mixed success in using machine learning to predict these outbreak-based estimates of $R_{0}$ from climatic, demographic, and other forms of nonoutbreak data, suggesting useful but complex associations between these variables and $R_{0}$ . From there, we showed through variance partitioning that $R_{0}$ (via the effective reproduction number, $R_{e}$ ) is itself only somewhat predictive of outbreak magnitude and timing. Together, these results show that the relationship between commonly considered predictor variables and quantifiable features of chikungunya outbreaks is far from straightforward.

### Outbreak data and epidemiological parameters

There is tremendous variability in what types of data get collected during or after outbreaks of chikungunya and other diseases. For many outbreaks in this study, the only datum available was an estimated attack rate, while, for a few outbreaks, there were additional data on serology and cases per household (section S1.2). These small datasets and varying data types can make it challenging to gain quantitative insights into CHIKV transmission dynamics. Therefore, one of our major contributions was estimating several epidemiological parameters—including $R_{0}$ , preexisting immunity, transmission heterogeneity, generation interval length, and mean transmission distance—for each of 86 different outbreaks of chikungunya. Unlike outbreak-specific datasets, these parameters quantify different aspects of how CHIKV is transmitted, are directly comparable across outbreaks, and can be used to simulate the impacts of interventions. Other studies have estimated or compiled published estimates of $R_{0}$ and immunity for smaller sets of chikungunya outbreaks (*13*, *67*, *68*), but we are not aware of any that have used a single framework to derive parameter estimates for so many outbreaks at once.

Across all chikungunya outbreaks that we considered, posterior median estimates of $R_{0}$ were between 1.5 and 3.2. Among the $R_{0}$ values compiled by Liu *et al.* (*13*) were previously published estimates for seven outbreaks considered in this study: $R_{0}$ estimates for each of Anzio and Guardavalle Marina, Italy (*69*); two $R_{0}$ estimates for Castiglione di Cervia and Castiglione di Ravenna, Italy (*70*, *71*); a single $R_{0}$ estimate for all three outbreaks in Venezuela (*41*); and an $R_{0}$ estimate for Trapeang Roka, Cambodia (*72*). On average, our $R_{0}$ estimates were about 30% lower than these published ones. The source of this discrepancy is unclear, although our use of a stochastic model is one possible explanation: rather than fitting the expected transmission dynamics to the available data, we fit the dynamics conditional on the outbreak resulting in a nonzero number of cases. This may have allowed us to fit the data using lower $R_{0}$ values, even if they resulted in smaller outbreaks on average.

We estimated the level of preexisting immunity to CHIKV, $p_{\text{immune}}$ , for the 39 outbreaks in populations where chikungunya outbreaks may have previously occurred. Across these populations, 95% of posterior estimates of $p_{\text{immune}}$ were below 70%, and the greatest median estimate for a single population was 37%. Because we stipulated that a cluster of chikungunya cases is an “outbreak” if it contains at least 50 cases in a clearly defined location and time period, it seems that immunity greater than 70% makes this unlikely to occur. This is stricter than the classical requirement that $R_{e}>1$ (*7*), which for our average $R_{0}=2.2$ demands that immunity be less than 55%. However, the lower median immunity estimates suggest that populations with immunity above 50% are at low risk for CHIKV transmission.

### To what extent is $R_{0}$ predictable for CHIKV?

The basic reproduction number, $R_{0}$ , is widely used to summarize the net effect of many factors on the potential for pathogen transmission. For CHIKV, DENV, ZIKV, and other pathogens transmitted by *Aedes* mosquitoes, numerous studies have investigated or proposed relationships between $R_{0}$ and predictors such as temperature, vegetation cover, economic indices, and more (*13*, *21*–*31*, *73*–*75*). The present study is unique in its use of a large set of $R_{0}$ estimates all derived from the same transmission model, which removes differences in model design and estimation methods as potential sources of variance. This study is also among the first to use machine learning to identify predictors of $R_{0}$ , rather than using linear regression or mechanistic models and choosing a set of predictors like temperature ahead of time. Overall, our regression experiments supported the hypothesis that $R_{0}$ is associated with various biotic, demographic, and climatic data, although whether these associations are useful for predicting $R_{0}$ was ambiguous.

Two trends appeared clear from our posterior estimates of $R_{0}$ , even before running the regression experiments: $R_{0}$ differed across WHO regions and CHIKV lineages. Larger $R_{0}$ values were associated with outbreaks in the Western Pacific and outbreaks caused by the IOL, and smaller $R_{0}$ values were associated with outbreaks in Africa and outbreaks caused by the ECSA lineage. These trends warrant further study. Le Viet *et al.* (*76*) suggested that, for DENV, outbreaks on islands can be particularly severe because infrequent viral introductions allow population susceptibility to replenish between outbreaks. Many of the Western Pacific populations that we considered were on islands; if this hypothesis holds for CHIKV as well, then it could have biased the associated $R_{0}$ estimates upward. On the other hand, our sample of 86 outbreaks only included 11 outbreaks in the Western Pacific region. Moreover, different regions have historically been affected by each lineage, and it is unclear whether differences in $R_{0}$ across lineages are responsible for the apparent differences across regions (or vice versa). Because all six of the outbreaks in Africa that we considered were caused by the ECSA lineage, it is impossible to determine which factor was responsible for those outbreaks’ lower $R_{0}$ estimates. Disentangling these relationships may require a broader, more geographically balanced analysis of chikungunya epidemiology.

Among the climate-informed predictors that we considered, four were positively associated with $R_{0}$ in at least one regression experiment: relative humidity, the occurrence probability of the *Aedes* vector responsible for each outbreak (*21*), relative $R_{0}$ [a nonlinear function of temperature and vector species; (*29*)], and Index-*P* [a nonlinear function of temperature and relative humidity; (*26*)]. The best regression models that we fit mainly used these predictors’ values 1 to 3 months before each outbreak. This agrees with observations by Nakase *et al.* (*22*), Burgueño *et al.* (*77*), Lowe *et al.* (*78*), and Shocket *et al.* (*79*), who noted similar delays between climatic conditions and the transmission of CHIKV, DENV, and Ross River virus. Mechanisms underlying this delay could include lags between weather events and mosquito population responses (*80*) and the intrinsic and extrinsic incubation periods during viral transmission (*60*), as well as uncertainty regarding when chikungunya outbreaks begin due to misdiagnosis or incomplete surveillance.

We observed a negative association between the potential for CHIKV transmission, as quantified by $R_{0}$ , and the potential for DENV transmission, as quantified by the FOI estimates mapped by Cattarino *et al.* (*23*). This unexpected trend likely reflects the noncausal nature of the machine learning models used here and by Cattarino *et al.* (*23*) and should be interpreted cautiously. Although we expected that past DENV transmission would associate positively with recent CHIKV transmission due to shared vector ecology, clearly neither process causes the other, and there are likely other drivers of transmission unique to either pathogen. Additionally, all model-based interpolations of epidemiological data, including those developed by Cattarino *et al.* (*23*), are uncertain. The DENV FOI values that we used in our regression should be regarded as estimates and may be particularly uncertain for heterogeneous regions where DENV epidemiological data were not available, including most islands included in this study. Last, it is possible that DENV and CHIKV transmission are positively associated due to shared vector ecology, but this association is confounded by variables excluded from our analysis. A hypothetical example could be disease prevention: If populations with previous DENV transmission are more likely to adopt preventative measures that limit subsequent CHIKV transmission, then the pathogens’ transmission patterns could appear negatively correlated. Our study and that by Cattarino *et al.* (*23*) aimed to predict transmission potential with minimal assumptions regarding its mechanisms, and machine learning is ideal for that task. However, different approaches would be required to make causal inferences.

Consistently accurate predictions of $R_{0}$ were elusive in our analysis. The apparent fit of the best machine learning models was sensitive to the choice of training and validation datasets, with a mean of $R^{2}=0.10$ , but $R^{2}<0$ on some individual validation folds. This sensitivity suggests that the associations identified above are context dependent and do not apply equally to all sets of outbreaks. For instance, the effects of mosquito abundance and climate on CHIKV transmission might differ across urban and rural contexts due to ecological differences or across poor and wealthy populations due to housing quality. Obtaining more consistent predictions of $R_{0}$ across at-risk populations could require analyzing a broader, more diverse set of chikungunya outbreaks, as well as accounting for causal relationships between variables and integrating data on confounding variables, as discussed in the previous paragraph.

### What drives variability in chikungunya outbreak severity?

Among CHIKV outbreaks that resulted in at least 50 infections, we determined that a large fraction of the variance in outbreak severity (6 to 34% across different outbreak features) was driven by stochasticity. In a sense, this figure underestimates the role of stochasticity, because whether a pathogen introduction results in a detectable outbreak of this size is also random (*7*). Aside from stochasticity, differences between populations’ network structures (that is, sizes and spatial configurations) were an important source of variance in outbreak magnitude (infection attack rate and peak incidence), explaining a similar amount of variance to $R_{e}$ . This agrees with a growing body of work emphasizing how even when $R_{0}$ is held constant, differences in network structure can lead to enormous variability in transmission dynamics (*35*, *37*, *81*–*84*).

Differences in epidemiological parameters besides $R_{0}$ also drove variability in chikungunya outbreak severity. Variability in outbreak magnitude was mainly driven by variability in the mean transmission distance, population immunity, the probability of within-house transmission, and the expected number of transmissions per infected person (parameters *L*, $p_{\text{immune}}$ , $p_{\text{house}}$ , and *B*, respectively). Variability in outbreak timing (duration and peak time) was driven by variability in the mean generation interval length and transmission heterogeneity transmission heterogeneity (*G* and *C*, respectively), in addition to immunity and within-house transmission. While we did not explicitly model mosquito population dynamics, we suspect that differences in *B* and *C* across populations reflected differences in mosquito abundance and population patchiness, respectively. Differences in generation interval length mainly reflect differences in temperature and vector species, which were incorporated into our priors on *G*. Temperature and vector species can also drive variability in mean transmission distance through their effects on mosquito flight distance; however, differences in transmission distance across populations might also reflect human movement patterns (*32*, *85*) or architecture and spatial barriers. Last, differences in preexisting immunity to CHIKV reflect populations’ epidemiological histories, including the sizes and time elapsed since previous chikungunya outbreaks (*68*).

The contributions of these parameters and others to transmission potential are, in theory, captured by $R_{e}$ . However, we found that, in total, variability in $R_{e}$ explained a relatively small fraction of variance in outbreak severity compared with stochasticity and population network structure, only 21% for infection attack rate and less for peak incidence and outbreak timing. Differences in $R_{0}$ , which ignores the role of immunity, would explain even less outbreak variability. This suggests that, for CHIKV and, likely, other pathogens, prior publications may be inadvertently overstating the value of $R_{0}$ and $R_{e}$ as single-variable summaries of transmission potential (*86*).

### To what extent is chikungunya outbreak severity predictable?

In the previous section, we used the variance partition and sensitivity analysis to identify causes of variability in outbreak severity. Those results can also be used to measure the predictability of outbreak severity. By predictability, we mean level of certainty with which we can estimate the severity of an outbreak before it occurs, using a model and data on the conditions in the affected population. We found that 80 to 85% of the variance in outbreak size and duration was due to differences between populations and, in theory, is predictable. The peak incidence of CHIKV infections was more predictable than outbreak size and duration (94% of variance explained), but the timing of that peak was less so (66% of variance explained). The remaining variance was due to stochasticity, which limits the certainty with which these outbreak features may be predicted. However, this study demonstrates how stochastic modeling enables researchers to quantify the extent of this uncertainty.

In practice, predicting the severity of a chikungunya outbreak with this promised 66 to 94% certainty is difficult. First of all, prediction requires an accurate model of how transmission unfolds. When studying data from simulated outbreaks, our model provides a perfect representation of those outbreaks’ dynamics. While our model fits well to data from real chikungunya outbreaks (see section S3.4), any residual disagreement with the real transmission process could reduce our ability to predict severity. Second, we determined that population network structure was a particularly important source of variability in outbreak severity. Measuring the contact network of a study population without prior transmission data is challenging, as is summarizing the relevant features of a network in a convenient way that enables predictions of outbreak dynamics (*32*, *37*, *84*). Last, obtaining accurate predictions of outbreak severity requires knowledge of a study population’s epidemiological parameters. In this study, we demonstrated the difficulty of estimating $R_{0}$ before an outbreak. Levels of preexisting immunity, $p_{\text{immune}}$ , could potentially be measured directly for study populations, but even $R_{0}$ and $p_{\text{immune}}$ combined (i.e., $R_{e}$ ) do not explain enough variance in outbreak severity to result in highly certain predictions. On the other hand, leveraging estimates of $R_{0}$ , $p_{\text{immune}}$ , and other parameters like transmission distance, generation interval length, and transmission heterogeneity could greatly improve predictions of chikungunya outbreak severity. Recent studies, including some that informed our prior on *G*, already use climatic and other data to estimate the generation (or serial) interval length for *Aedes*-borne pathogens (*26*, *30*, *57*, *60*). However, models for predicting transmission distance or heterogeneity from similar data sources appear to be lacking. Thus, predicting chikungunya outbreak severity with minimal uncertainty will require accurate modeling, careful consideration of often-overlooked network structures, and innovations for estimating key epidemiological parameters before outbreaks begin.

Our analysis of the predictability of outbreak severity was not without limitation. One clear example is that our ability to predict $R_{0}$ using non-outbreak data was determined by the potential predictors we considered. While we exhaustively tested many combinations of demographic predictors, climatic predictors, and time lags, it remains possible that different ways of using climatic data [e.g., diel temperature ranges; (*31*)], as well as different data sources entirely, may have resulted in better estimates of $R_{0}$ . Additional data on vector control efforts, housing quality, and urbanness may be particularly useful for improving estimates of $R_{0}$ , although these are typically less readily available than climatic variables.

More generally, any analysis of outbreak-based $R_{0}$ estimates is likely to be biased toward locations where sufficiently large outbreaks have been documented. Although the 86 affected populations that we considered were diverse, including urban and rural populations on all continents where CHIKV transmission is known to have occurred, this sample was inherently biased toward sites where CHIKV introductions were more likely to occur, CHIKV transmission was more likely to be intense, and chikungunya cases were more likely to be detected. In practice, this bias skewed our study heavily toward Southeast Asia and the Americas, despite the long and ongoing history of CHIKV circulation in Africa (*87*). Such a data imbalance could have limited diversity across study populations and $R_{0}$ estimates, making predicting $R_{0}$ from site features more challenging. Moreover, because the WHO region in which each outbreak occurred was an important predictor of $R_{0}$ in our regression analysis, our models cannot estimate the $R_{0}$ of CHIKV in previously unaffected regions. One example is Australia (although it is technically part of the diverse Western Pacific region), which has not documented autochthonous CHIKV transmission but is believed to be at risk for chikungunya due to its climate and connectedness to South East Asia (*88*). To the extent that chikungunya outbreak severity is predictable, more work is needed to accurately map the landscape of chikungunya risk in neglected or previously unaffected regions.

For outbreak-prone pathogens like CHIKV, the enduring challenge of anticipating severe outbreaks stymies public health preparedness efforts. We presented a data-driven framework for assessing the feasibility of predicting outbreak severity and identifying targets for improving current approaches. For CHIKV specifically, we estimated $R_{0}$ and other parameters for numerous recent outbreaks, quantifying several features of chikungunya’s epidemiology and enabling outbreak simulations. However, predicting chikungunya outbreak severity was only somewhat feasible using single-variable summaries of transmission potential like $R_{0}$ . Information on factors such as generation interval length, transmission distance, and the spatial configurations of at-risk populations could result in more accurate predictions, although it is unclear how or if these factors may be quantified before outbreaks begin. Pending further research on the factors that drive this variability and how that variability translates into differences in outbreak severity, the outcomes of future chikungunya outbreaks will remain difficult to predict.

## MATERIALS AND METHODS

### Review of published chikungunya outbreak data

Between July and September of 2021, we searched GIDEON (*89*), Google, Google Scholar, and PubMed for publications documenting local transmission of CHIKV. In 2022, we updated this search with publications referenced in the review of chikungunya epidemiology by Bettis *et al.* (*48*). See section S1.1 for details. We defined a chikungunya outbreak as a cluster of chikungunya cases meeting the following requirements:

1) Size: Cluster must include at least 50 documented chikungunya cases.

2) Population: Affected population must be clearly identified and geographically contiguous.

3) Local transmission: Description of the case cluster must suggest that at least some cases were due to autochthonous CHIKV transmission (i.e., not all cases were imported).

4) Completeness: Description of the cluster must suggest that no additional transmission occurred before or after the documented cases.

5) Data availability: Description of the cluster must include sufficient detail to estimate the final attack rate of chikungunya in the affected population.

We identified 142 unique chikungunya outbreaks using this definition. Next, we then imposed two additional criteria for inclusion in this study. First, due to the computational costs of modeling and parameter estimation (which increase with population size), we excluded outbreaks in populations larger than 150,000 individuals. This excluded 28 outbreaks, leaving 114. Second, to avoid biasing the set of affected populations toward regions with more extensive data collection, we included only one CHIKV outbreak per affected population and, at most, three outbreaks per administrative level 1 (e.g., state), CHIKV lineage, and 5-year period. This criterion was motivated by studies such as those by Dwibedi *et al.* (*90*) in India, Malik *et al.* (*54*) in Yemen, Rodriguez-Morales *et al.* (*91*, *92*) in Colombia, and Zambrano *et al.* (*93*) in Honduras that reported dozens of outbreaks during waves of CHIKV transmission across states or smaller regions. This condition excluded another 28 outbreaks, leaving the 86 investigated by this study.

### Model of CHIKV transmission

We recreated the transmission dynamics of each of the 86 outbreaks in this study using the CHIKSIM model from Meyer *et al.* (*38*). CHIKSIM is coded in version 1.11.2 of the Julia Language (*94*). Here, we summarize the model’s most salient features and parameters; for a full description, see (*38*).

CHIKSIM provided stochastic, spatially explicit, and individual-based simulations of CHIKV transmission within contiguous populations. The model consisted of a population submodel, a transmission submodel, and a data submodel. The population submodel generated realistic maps of household locations for each of the 86 affected populations. For each population, we obtained total population size and household count estimates in outbreak years either from publications documenting each outbreak or else using data from ArcGIS (*95*) and the World Bank (*96*). Locations of simulated households were sampled from WorldPop’s 2020 UN-adjusted constrained population count maps with 100 m by 100 m grid cells (*97*). Each household was initialized with one individual; all remaining individuals were randomly assigned to households. This procedure resulted in simulated populations with correct total sizes, spatial distributions, and mean household sizes.

The transmission submodel simulated CHIKV outbreaks in the populations generated by the population submodel. Each simulation was initialized with $I_{0}\geq 1$ randomly selected individuals infected with CHIKV. For outbreaks in Europe and the Americas, as well as those described by publications as the first in their affected countries, we assumed that the remainder of the population was susceptible to CHIKV. For all other outbreaks, we assumed that a fraction of the affected population $p_{\text{immune}}\in \left[0,1\right]$ had infection-blocking immunity to CHIKV due to prior exposure. Each infected individual was the source of a random number of transmissions to nearby individuals. For an individual in household *h*, the expected number of such transmissions was $B_{h}$ [ $\beta_{h}$ in (*38*)]. Across households, the values $B_{h}$ had mean B>0 and CoV C>0 across households [ β and *c*, respectively, in (*38*)], capturing house-to-house differences in human-mosquito contact. We assumed that, of these transmissions, a fraction $p_{\text{house}}\in \left[0,1\right]$ were received by members of the same household as their source. The remaining transmissions were received by members of any household, with mean transmission distance L>0 m. The expected time between the start of a source individual’s infection and that of any recipient was the generation interval, G>0 days [ λ in (*38*)].

Multiple transmissions may be received by the same individual (including the source of those transmissions), resulting in a basic reproduction number $R_{0}\leq B$ . We estimated $R_{0}$ empirically for each population and parameter set by computing the average number of unique transmission recipients per source infection, excluding the source individuals themselves. Note that $R_{0}$ was not technically a model parameter, but rather an emergent property of other parameters’ values and interactions with the population.

For each of the 86 affected populations, we reduced the available data to a set of up to seven summary statistics, depending on available data, to facilitate parameter estimation via approximate Bayesian computation (see the “Model calibration and parameter estimation” section). The statistics for each outbreak are listed in section S1.2. We constructed separate data submodels for each outbreak to extract the relevant summary statistics from the output of the transmission submodel. All data submodels included the possibility of asymptomatic CHIKV infections and unreported or undiagnosed chikungunya cases, which are both considered important aspects of chikungunya’s epidemiology (*56*, *98*, *99*). In particular, we assumed that a fraction $p_{\text{asymp}}\in \left[0,1\right]$ of all CHIKV infections were asymptomatic, and only a fraction $p_{\text{report}}\in \left[0,1\right]$ of the symptomatic infections were included in the data.

### Model calibration and parameter estimation

We used Bayesian inference to estimate the nine model parameters (excluding $R_{0}$ ) in Table 1. By estimating these parameters separately for each outbreak, we accounted for spatial variability in transmission among affected populations at the time of each outbreak. We used time-constant parameters for each outbreak, under the assumption that most chikungunya outbreaks are short compared with the timescale of local climatic variability.

We derived priors for each parameter based on published data from chikungunya outbreaks not included in this study, outbreaks of DENV (which is transmitted by the same mosquito species as CHIKV), and heuristic arguments. For the transmission parameter *B*, we fitted a prior to $R_{0}$ estimates for CHIKV outbreaks tabulated by Liu *et al.* (*13*). For the heterogeneity parameter *C*, we used a prior that assumed CHIKV transmission follows the “80/20 rule,” in which 80% of transmissions are caused by 20% of the infected population (*4*). For all affected populations that were not argued to be naive to CHIKV, we used a prior on immunity, $p_{\text{immune}}$ , fitted to data from CHIKV serological studies tabulated by Fritzell *et al.* (*67*); otherwise, we assumed a priori that $p_{\text{immune}}=0$ . For the probability of asymptomatic infeciton, $p_{\text{asymp}}$ , we fitted lineage-specific priors to estimates of the probability of inapparent CHIKV infection tabulated by Bustos Carrillo *et al.* (*56*). After omitting data from outbreaks included in this study, no estimates from outbreaks of the IOL of CHIKV remained; therefore, we used the same prior on $p_{\text{asymp}}$ for outbreaks of the IOL as for ECSA lineage, of which the IOL is a sublineage. Given variability in how chikungunya cases were documented across populations, we used a relatively uninformative prior for $p_{\text{report}}$ that assumed moderate values were more likely than extreme ones. For $p_{\text{house}}$ , we based our prior on the estimated probability of within-house DENV transmission from Cavany *et al.* (*100*). For transmission distance *L*, we fitted a prior to five estimates of the mean transmission distance of DENV. For initial infections $I_{0}$ , our prior assumed that the initial number of infections in each outbreak was, on average, similar to the size of one household. Last, for mean generation interval *G*, we derived an *Aedes* species– and temperature-specific prior on the basis of published models of serial interval length (*57*) and mosquito mortality (*58*). See section S3.1 for full details.

Because outbreak data often follow complicated or unknown distributions and different types of data were available for each outbreak, specifying a likelihood function analytically for each outbreak was infeasible. Instead, we used the simulation-based likelihood estimation approach presented by Meyer *et al.* (*38*). Like other approximate Bayesian computation methods, this approach required us to reduce each outbreak’s data to a small number (median of 2, mean of 2.4, and maximum of 7) of summary statistics, such as total reported chikungunya cases (*101*). See section S1.2 for details. For each outbreak, we sampled 100,000 parameter sets from the prior described above. We estimated each parameter set’s likelihood using 250 simulations. To sample from each outbreak’s posterior parameter distribution, we redrew parameter sets from the prior sample using the computed likelihoods as weights. We computed a posterior estimate of $R_{0}$ from each parameter set using the procedure described in the “Model of CHIKV transmission” section. Last, for each of the 86 real outbreaks, we simulated 1000 outbreaks using posterior parameter combinations to verify that we had successfully calibrated CHIKSIM to the available data (section S3.4).

Following Meyer *et al.* (*38*), we evaluated the quality of our parameter inferences using 100 simulated datasets (with known parameter values) for each of the 86 outbreaks. For each outbreak and each parameter, we used the inferences on the simulated data to compute the coverage, bias, and accuracy of our method applied to the available data. See section S3.3.1 for details.

### Predicting $R_{0}$ from nonoutbreak data

We used machine learning to measure the extent to which our outbreak-based estimates of $R_{0}$ could have been predicted using the nonoutbreak data sources in Table 2. First, we constructed a dataset of these predictors and our posterior mean $R_{0}$ estimates. To account for the different levels of uncertainty in the outbreaks’ $R_{0}$ estimates, we drew a weighted sample of 1000 rows from this initial dataset, using the precisions (the reciprocal of variance) of each outbreak’s $R_{0}$ estimates as weights. Next, we partitioned these data by grouping the 86 outbreaks into 10 sets, ensuring that all rows associated with each outbreak belonged to a single set.

We used nested cross-validation (with eight inner folds structured the same way) to tune and evaluate a suite of 60,672 models for predicting $R_{0}$ on these data. Each model used ridge regression, random forest, or *K*-nearest neighbors to estimate the posterior mean $R_{0}$ for each outbreak using a subset of the predictors in Table 2. These subsets included every combination of these predictors that contained viral lineage; however, to keep the number of models manageable, we allowed each model to use only a single time point (annual mean, outbreak start, or 1 to 3 months before the outbreak) for all predictors available a multiple time points. All models were fitted using the MLJ.jl package for the Julia Language (*102*). For each model (that is, combination of a regression algorithm with a set of predictors), we chose hyperparameters that minimized the total mean squared error between its out-of-sample predictions and the posterior mean $R_{0}$ estimates across inner cross-validation folds and then evaluated model fit using the $R^{2}$ between its predictions and the posterior mean $R_{0}$ estimates on a withheld outer cross-validation fold. We ranked the models by their mean $R^{2}$ across the 10 outer validation folds. We repeated this procedure three times to account for the effects of how the outbreaks were randomly partitioned into 10 validation folds. For each repetition, we also applied this procedure to a shuffled dataset in which $R_{0}$ estimates were permuted across outbreaks. Shuffling the data eliminated real-world trends, so our fit to these data established the fit that could be expected through randomness and overfitting alone.

We computed Shapley additive explanations, or SHAP values, to quantify the importance of each predictor used in the top 10 models trained with each partition on the real (not shuffled) $R_{0}$ dataset (*63*). Because not all models used all predictors, we pooled the SHAP values across each set of top models, resulting in a set of SHAP values for each partition.

### Partitioning variance in outbreak features

We devised a variance partition for four different aspects of outbreak severity: infection attack rate, outbreak duration, peak weekly incidence (normalized by population size), and peak timing. The five sources of variance that we considered were parameter differences among populations, parameter uncertainty for each population, differences in network structure among populations, the interaction of parameters with network structure, and stochasticity.

To separate the effects of parameter differences and network structure among populations, we simulated severity data using all possible pairs of the 86 affected populations and their posterior parameter distributions. To separate variance in these simulations due to parameter uncertainty from variance due differences across populations, we included 10 parameter samples from each posterior distribution. This resulted in a total of 86×86×10 scenarios. We simulated multiple outbreaks for each scenario to capture the effects of stochasticity. Because we were specifically interested in visible outbreaks, rather than CHIKV introductions, we applied a similar inclusion criterion to these simulations as to the real outbreaks: Each must have resulted in at least 50 CHIKV infections. We focused on infections, rather than reported cases, to exclude the probability of detection as a source of variance in the infection attack rate. We ran batches of 50 simulations at a time until either 25 had met this criterion or we reached 2500 simulations total. To reduce the possibility of running 2500 simulations without including at least 25, we only considered parameter combinations for which $R_{e}\geq 1$ . We excluded a small number of parameter-population combinations for which fewer than three of 2500 simulations resulted in 50 infections, because computing a variance requires at least three samples.

Here, we motivate the derivation of the five-term variance partition used in Results. For the full derivation, as well as an alternative four-term partition that uses parameter uncertainty differently, see section S5.

We log-transformed the four aspects of outbreak severity listed above, because they varied across orders of magnitude. Let the random variable *Y* denote the log-transformed value of an outbreak feature (e.g., infection attack rate) across simulated outbreaks. Each simulation produces a realization of *Y*, which depends on the population on whose network the simulation took place, *N*, the population whose posterior parameter distribution was used for the simulation, *D*, and the specific parameter set drawn from that distribution, *P*. From the law of total variance (*103*), the variance of *Y* can be partitioned into variance explained by these nonstochastic factors, $V_{\text{nonstoch}}$ , and variance within each scenario that is not explained by these factors (and, therefore, is explained by stochasticity), $V_{\text{stoch}}$

$$\begin{array}{c} \text{Var}\left(Y\right)=\underset{V_{\text{nonstoch}}}{\underbrace{\text{Var}\left[E\left(Y\;|\;N,D,P\right)\right]}}+\underset{V_{\text{stoch}}}{\underbrace{E\left[\text{Var}\left(Y\;|\;N,D,P\right)\right]}} \end{array}$$ (3)

Because $Y_{\mathrm{NDP}}=E\left[Y\;|\;N,D,P\right]$ is a random variable, we can use a similar approach to partition its variance, $V_{\text{nonstoch}}$ . Let $V_{\text{pars}}$ represent the variance in *Y* due to parameter differences only, $V_{\text{network}}$ represent the variance due to network differences only, $V_{\text{ixn}}$ represent the variance due to the interactions of parameter and network differences, and $V_{\text{unc}}$ represent the variance due to parameter uncertainty. By applying the law of total variance to $Y_{\mathrm{NDP}}$ three different ways, we obtain the variance partition

$$V_{\text{nonstoch}}=V_{\text{pars}}+V_{\text{unc}}+V_{\text{ixn}}+V_{\text{network}}$$ (4)

Last, substituting this expression into Eq. 3 and dividing by Var(Y) result in the percentages of variance in *Y* due to parameters, network structure, the interaction thereof, uncertainty, and stochasticity shown in Fig. 4A.

### Global sensitivity analysis

Variance-based sensitivity analyses, such as the Sobol’ method, compute the fraction of the variance in a deterministic model output attributable to each of several parameters, either per se (first-order sensitivity indices) or considering interactions with all other parameters (total sensitivity indices) (*104*). Such an analysis could be applied to the average behavior of the CHIKSIM model, but the number of new simulations required to do so via the Sobol’ method was prohibitive. Instead, we used the effective algorithm for computing global sensitivity indices (EASI) method of Plischke (*66*), which can approximate first-order sensitivity indices using existing simulations, such as those we used for the variance partition. We used the implementation of EASI in the Julia package GlobalSensitivity.jl (*105*) to obtain the sensitivity indices in Fig. 4B. In general, first-order sensitivity indices sum to less than one because they exclude variance due to parameters’ interactions. Therefore, the differences between one and these sums represented variance in mean model behavior attributable to those interactions.

## Supplementary Materials

### The PDF file includes:

Supplementary Text Figs. S1 to S24 Table S1 Legends for data S1 and S2 References
