# Supplementary material for: Predictability of infectious disease outbreak severity: Chikungunya as a case study
Source: Sci Adv. 2025 Oct 3;11(40):eadt5419. doi: 10.1126/sciadv.adt5419 (PMC12494021; doi:10.1126/sciadv.adt5419)
Supplement: Supplementary file 1 — Supplementary Text Figs. S1 to S24 Table S1 Legends for data S1 and S2 References [file sciadv.adt5419_sm.pdf]

Science Advances
AAAS

Supplementary Materials for

Predictability of infectious disease outbreak severity: Chikungunya as a case study

Alexander D. Meyer *et al.*

Corresponding author: Alexander D. Meyer, ameyer7@nd.edu

Sci. Adv. **11**, eadt5419 (2025)
DOI: 10.1126/sciadv.adt5419

The PDF file includes:

Supplementary Text
Figs. S1 to S24
Table S1
Legends for data S1 and S2
References

Other Supplementary Material for this manuscript includes the following:

Data S1 and S2

Supplemental Text

S1 Review of published chikungunya outbreak data

S1.1 Literature review protocol

We used five sources to identify chikungunya outbreaks: the Global Infectious Disease and Epidemiology Online Network (GIDEON) database [89], Google search, Google Scholar, PubMed, and the recent review of chikungunya outbreaks by Bettis et al. [48]. The first four sources were accessed between July and September of 2021.

In the GIDEON database, we used the map function to visualize existing records of chikungunya outbreaks. For each country or territory with a record of an outbreak, we reviewed the corresponding GIDEON page. We followed up on any reference therein containing the word "outbreak". Records of outbreaks meeting the criteria in the main text (at least 50 cases in a clearly defined timespan and population smaller than 150,000, with evidence of local transmission and enough data to estimate an attack rate) were included in our review. If a reference led to a page in the International Society for Infectious Diseases' ProMED website, we searched that page and references therein using the same protocol.

We uncovered additional records of outbreaks by searching Google and Google Scholar for the phrase "chikungunya outbreak in [region]" for the following regions: Brazil (Fortaleza, Recife), Cambodia, Ecuador, Guatemala, Kenya, Nicaragua, Peru (northern), Republic of Congo. In PubMed, we used the searches "chikungunya AND [region]" for the same set of regions. Resulting papers were examined for records of chikungunya outbreaks meeting the criteria above. Our searches of Google, Google Scholar, GIDEON, and PubMed yielded 130 articles, 40 of which contained records of outbreaks meeting our criteria.

Finally, in 2023 we reviewed all studies referenced in the main text and supplement of Bettis et al. [48] that were not already identified by our 2021 searches. This yielded another 77 articles, of which 20 contained records of outbreaks meeting our criteria.

The 60 included articles contained records of 114 outbreaks. As indicated in the main text, we randomly discarded outbreaks to obtain a sample with no more than one outbreak per administrative level 1, per CHIKV lineage, per five-year period. This resulted in the 86 outbreaks used for our analysis.

S1.2 Descriptions of the 86 outbreaks and available data

Here, we list references, available data, and other information for the 86 outbreaks included in this study.

Unless otherwise specified, population density maps of the affected sites were obtained by finding the latitude/longitude coordinates of the location's outline from ArcGIS [95] or Google Maps [117], then extracting the outlined region from WorldPop's 2020 UN-adjusted unconstrained population density maps with 100 m $\times$ 100 m grid cells [97]. We then adjusted the total population size within the extracted map to match the population size reported in the outbreak's associated publication, if available. If not, we used country-level population growth data from the World Bank [96] to adjust WorldPop's 2020 estimates to the year in which the outbreak began. We obtained the mean household size in each affected population using information from each outbreak's associated publication, or else administrative level 4 averages from ArcGIS.

For eight outbreaks, serological tests for immunoglobulin-M or -G (IgM and IgG, respectively) were used to confirm chikungunya cases and identify asymptomatic infections. Real-time polymerase chain reaction (RT-PCR) was used to confirm cases as well, and occasionally to assay for asymptomatic infections. These tests all differ with respect to who tests positive, and when [118, 119, 120]. However, due to the inconsistent information available on each outbreak investigation's exact protocol, we made simplifying assumptions while modeling these data. We assumed that RT-PCR is only useful for confirming symptomatic cases, since its sensitivity for asymptomatic infections is not well understood [120]. For IgG, we assumed that individuals with pre-existing immunity and individuals infected during the simulated outbreak both tested positive, since IgG positivity after infection usually lasts for life. In contrast, we assumed that only individuals infected during the simulated outbreak tested positive for IgM, since IgM positivity only lasts for months to years [121], much less than the typical time between outbreaks in a single population [68]. In real life, IgM and IgG are not detectable until a few days after the start of infection [118]. We did not model this delay because in outbreak investigations, the timing of testing relative to infection was unclear.

BGD-01: Char Kushai, Bangladesh. *Study:* Khatun et al. [122]. *Population size:* 3,840 individuals in 897 households. *Outbreak year:* 2011. *Vector species: Aedes albopictus*. *CHIKV lineage:* IOL. *CHIKV-naive:* No. *Available data:* 1,105 cases; 263 households with one case; 178 households with two cases; 133 households with at least three cases.

BGD-02: Palpara Village, Bangladesh. *Study:* Salje et al. [32]. *Population size:* 1,933 individuals in 460 households. Map extracted from Figure 1A of Salje et al. [32]. *Outbreak year:* 2012. *Vector species: Aedes aegypti*. *CHIKV lineage:* IOL. *CHIKV-naive:* No. *Available data:* 364 cases over 186 days; peak incidence of 62 cases per week after 133 days.

BRB: Bridgetown, Barbados. *Study* Kumar et al. [123]. *Population size:* 85,699 individuals in 29,551 households. *Outbreak year:* 2014. *Vector species: Aedes aegypti*. *CHIKV lineage:* Asian.

CHIKV-naive: Yes. *Available data:* Among a sample of 1,983 children – 203 cases over 183 days; peak incidence of 73 cases per month after 106 days. Since our model does not include age structure, we recreated the sample of 1,983 children by selecting 1,983 random individuals.

BTN-01: Samtse District, Bhutan. *Study:* Wangchuk et al. [124]. *Population size:* 58,898 individuals in 14,365 households. *Outbreak year:* 2012. *Vector species: Aedes aegypti. CHIKV lineage:* ECSA. *CHIKV-naive:* No. *Available data:* 90 cases.

BTN-02: Thimphu District, Bhutan. *Study:* Wangchuk et al. [124]. *Population size:* 130,552 individuals in 31,084 households. *Outbreak year:* 2012. *Vector species: Aedes aegypti. CHIKV lineage:* ECSA. *CHIKV-naive:* No. *Available data:* 76 cases.

BRA-01: Alto do Cemiterio, Brazil. *Study:* Dias et al. [125]. *Population size:* 35,496 individuals in 12,240 households. *Outbreak year:* 2015. *Vector species: Aedes aegypti. CHIKV lineage:* ECSA. *CHIKV-naive:* Yes. *Available data:* 1,536 cases.

BRA-02: Chapada District, Brazil. *Study:* Cunha et al. [44]. *Population size:* 2,303 individuals in 523 households. *Outbreak year:* 2014. *Vector species: Aedes aegypti. CHIKV lineage:* ECSA. *CHIKV-naive:* Yes. *Available data:* 90 cases over 396 days; peak incidence of 39 cases per month after 35 days. Additionally, among 120 individuals from 45 randomly selected households, 24 tested positive for anti-CHIKV antibodies (IgM and IgG, but same individuals should test positive within this naive population), and 13 of those 24 displayed symptoms of chikungunya. We recreated the sampling process of selecting 120 individuals from 45 random households, and assumed the 24 seropositive people had antibodies due to infection during this outbreak, rather than some previous exposure.

BRA-03: Coutos Neighborhood, Brazil. *Study:* Tauro et al. [39]. *Population size:* 662 individuals in 230 households. *Outbreak year:* 2017. *Vector species: Aedes aegypti. CHIKV lineage:* ECSA. *CHIKV-naive:* Yes. *Available data:* 50 cases over 71 days; peak incidence of eight cases per day after 30 days; 33 households had at least one case.

KHM: Trapeang Roka, Cambodia. *Study:* Ly et al. [126]. *Population size:* 694 individuals in 134 households. Map extracted from Figure 3 of Robinson et al. [72]. *Outbreak year:* 2012. *Vector species: Aedes aegypti. CHIKV lineage:* IOL. *CHIKV-naive:* No. *Available data:* Among a sample of 425 randomly selected individuals, 190 tested positive for anti-CHIKV IgM, 180 of those 190 had chikungunya-like symptoms, and 138 of those 180 knew the date of their symptoms' onset. As in Meyer et al. [38], we treated the 42 symptomatic infections with unknown start dates as "unreported" to match the terminology of our model. The 138 cases with known start dates occurred over 48 days, with a peak incidence of 15 cases per day after 41 days.

CHN: Xincun Community, China. *Study:* Qiaoli et al. [51]. *Population size:* 11,000 individuals in 3,929 households. *Outbreak year:* 2010. *Vector species: Aedes albopictus. CHIKV lineage:* ECSA. *CHIKV-naive:* Yes. *Available data:* 253 cases over 42 days; peak incidence of 43 cases

per three-day period after 36 days; 87 households were affected and 28 households had at least two cases.

COL-01: Calarca, Colombia. *Study:* Rodriguez-Morales et al. [91]. *Population size:* 77,398 individuals in 26,689 households. *Outbreak year:* 2014. *Vector species: Aedes aegypti*. *CHIKV lineage:* Asian. *CHIKV-naive:* Yes. *Available data:* 106 cases.

COL-02: La Dorada, Colombia. *Study:* Rodriguez-Morales et al. [91]. *Population size:* 76,769 individuals in 25,590 households. *Outbreak year:* 2014. *Vector species: Aedes aegypti*. *CHIKV lineage:* Asian. *CHIKV-naive:* Yes. *Available data:* 2,060 cases.

COL-03: La Tebaida, Colombia. *Study:* Rodriguez-Morales et al. [91]. *Population size:* 42,141 individuals in 13,169 households. *Outbreak year:* 2015. *Vector species: Aedes aegypti*. *CHIKV lineage:* Asian. *CHIKV-naive:* Yes. *Available data:* 335 cases.

COL-04: La Virginia, Colombia. *Study:* Rodriguez-Morales et al. [91]. *Population size:* 31,999 individuals in 11,034 households. *Outbreak year:* 2014. *Vector species: Aedes aegypti*. *CHIKV lineage:* Asian. *CHIKV-naive:* Yes. *Available data:* 491 cases.

COL-05: Morroa, Colombia. *Study:* Rodriguez-Morales et al. [92]. *Population size:* 14,263 individuals in 3,962 households. *Outbreak year:* 2014. *Vector species: Aedes aegypti*. *CHIKV lineage:* Asian. *CHIKV-naive:* Yes. *Available data:* 50 cases.

COL-06: Quimbaya, Colombia.*Study:* Rodriguez-Morales et al. [91]. *Population size:* 34,902 individuals in 12,035 households. *Outbreak year:* 2014. *Vector species: Aedes aegypti*. *CHIKV lineage:* Asian. *CHIKV-naive:* Yes. *Available data:* 97 cases.

COL-07: San Juan de Betulia, Colombia. *Study:* Rodriguez-Morales et al. [92]. *Population size:* 12,529 individuals in 3,480 households. *Outbreak year:* 2014. *Vector species: Aedes aegypti*. *CHIKV lineage:* Asian. *CHIKV-naive:* Yes. *Available data:* 172 cases.

COL-08: Tolu, Colombia. *Study:* Rodriguez-Morales et al. [92]. *Population size:* 32,731 individuals in 8,846 households. *Outbreak year:* 2014. *Vector species: Aedes aegypti*. *CHIKV lineage:* Asian. *CHIKV-naive:* Yes. *Available data:* 1,232 cases.

COL-09: Victoria, Colombia. *Study:* Rodriguez-Morales et al. [91]. *Population size:* 8,415 individuals in 3,005 households. *Outbreak year:* 2015. *Vector species: Aedes aegypti*. *CHIKV lineage:* Asian. *CHIKV-naive:* Yes. *Available data:* 108 cases.

COL-10: Viterbo, Colombia. *Study:* Rodriguez-Morales et al. [91]. *Population size:* 12,488 individuals in 4,163 households. *Outbreak year:* 2014. *Vector species: Aedes aegypti*. *CHIKV lineage:* Asian. *CHIKV-naive:* Yes. *Available data:* 70 cases.

DMA: Dominica (whole island). *Study:* Ahmed et al. [127]. *Population size:* 71,401 individuals

in 25,500 households. *Outbreak year:* 2013. *Vector species: Aedes aegypti. CHIKV lineage:* Asian. *CHIKV-naive:* Yes. *Available data:* 3,559 cases over 315 days; peak incidence of 240 cases per week after 98 days.

DOM-01: La Romana, Dominican Republic. *Study:* Langsjoen et al. [128]. *Population size:* 137,149 individuals in 40,338 households. *Outbreak year:* 2014. *Vector species: Aedes aegypti. CHIKV lineage:* Asian. *CHIKV-naive:* Yes. *Available data:* 194 cases over 91 days.

DOM-02: Nigua, Dominican Republic. *Study:* Pimentel et al. [129]. *Population size:* 21,150 individuals in 5,875 households. *Outbreak year:* 2014. *Vector species: Aedes aegypti. CHIKV lineage:* Asian. *CHIKV-naive:* Yes. *Available data:* 767 cases.

GAB-01: Franceville, Gabon. *Study:* Caron et al. [130]. *Population size:* 98,418 individuals in 24,004 households. *Outbreak year:* 2010. *Vector species: Aedes albopictus. CHIKV lineage:* ECSA. *CHIKV-naive:* No. *Available data:* 882 cases over 69 days; peak incidence of 234 cases per week after 30 days.

GAB-02: Koulamoutou, Gabon. *Study:* Caron et al. [130]. *Population size:* 22,832 individuals in 5,569 households. *Outbreak year:* 2010. *Vector species: Aedes albopictus. CHIKV lineage:* ECSA. *CHIKV-naive:* No. *Available data:* 64 cases over 69 days.

GAB-03: Lambarene, Gabon. *Study:* Caron et al. [130]. *Population size:* 32,384 individuals in 7,899 households. *Outbreak year:* 2008. *Vector species: Aedes albopictus. CHIKV lineage:* ECSA. *CHIKV-naive:* No. *Available data:* 80 cases over 274 days.

GAB-04: Lastourville, Gabon. *Study:* Caron et al. [130]. *Population size:* 10,014 individuals in 2,442 households. *Outbreak year:* 2008. *Vector species: Aedes albopictus. CHIKV lineage:* ECSA. *CHIKV-naive:* No. *Available data:* 63 cases over 274 days.

GAB-05: Libreville, Gabon. *Study:* Caron et al. [130]. *Population size:* 99,693 individuals in 24,315 households. *Outbreak year:* 2007. *Vector species: Aedes albopictus. CHIKV lineage:* ECSA. *CHIKV-naive:* No. *Available data:* 267 cases over 121 days.

GAB-06: Moanda, Gabon. *Study:* Caron et al. [130]. *Population size:* 52,654 individuals in 12,842 households. *Outbreak year:* 2010. *Vector species: Aedes albopictus. CHIKV lineage:* ECSA. *CHIKV-naive:* No. *Available data:* 147 cases over 69 days.

GRD: Grenada (whole island). *Study:* Macpherson et al. [131]. *Population size:* 108,781 individuals in 36,826 households. *Outbreak year:* 2014. *Vector species: Aedes aegypti. CHIKV lineage:* Asian. *CHIKV-naive:* Yes. *Available data:* 493 cases over 92 days.

HND-01: Aramecina, Honduras. *Study:* Zambrano et al. [93]. *Population size:* 7,308 individuals in 1,624 households. *Outbreak year:* 2015. *Vector species: Aedes aegypti. CHIKV lineage:* Asian. *CHIKV-naive:* Yes. *Available data:* 4,050.4 cases per 100,000 people (296 total).

HND-02: Caridad, Honduras. *Study:* Zambrano et al. [93]. *Population size:* 3,961 individuals in 880 households. *Outbreak year:* 2015. *Vector species: Aedes aegypti*. *CHIKV lineage:* Asian. *CHIKV-naive:* Yes. *Available data:* 4,822.5 cases per 100,000 people (191 total).

HND-03: Juticalpa, Honduras. *Study:* Zambrano et al. [93]. *Population size:* 129,875 individuals in 30,203 households. *Outbreak year:* 2015. *Vector species: Aedes aegypti*. *CHIKV lineage:* Asian. *CHIKV-naive:* Yes. *Available data:* 3,017.5 cases per 100,000 people (3,919 total).

HND-04: San Antonio de Flores, Honduras. *Study:* Zambrano et al. [93]. *Population size:* 5,455 individuals in 1,364 households. *Outbreak year:* 2015. *Vector species: Aedes aegypti*. *CHIKV lineage:* Asian. *CHIKV-naive:* Yes. *Available data:* 2,896.4 cases per 100,000 people (158 total).

HND-05: San Juan, Honduras. *Study:* Zambrano et al. [93]. *Population size:* 12,121 individuals in 2,474 households. *Outbreak year:* 2015. *Vector species: Aedes aegypti*. *CHIKV lineage:* Asian. *CHIKV-naive:* Yes. *Available data:* 3,787.9 cases per 100,000 people (459 total).

HND-06: Santa Rita, Honduras. *Study:* Zambrano et al. [93]. *Population size:* 4,005 individuals in 969 households. *Outbreak year:* 2015. *Vector species: Aedes aegypti*. *CHIKV lineage:* Asian. *CHIKV-naive:* Yes. *Available data:* 5,218.5 cases per 100,000 people (109 total).

HND-07: San Francisco de Ojuera, Honduras. *Study:* Zambrano et al. [93]. *Population size:* 7,288 individuals in 1,763 households. *Outbreak year:* 2015. *Vector species: Aedes aegypti*. *CHIKV lineage:* Asian. *CHIKV-naive:* Yes. *Available data:* 3,197.0 cases per 100,000 people (233 total).

HND-08: Santa Rosa de Copán, Honduras. *Study:* Zambrano et al. [93]. *Population size:* 63,829 individuals in 15,568 households. *Outbreak year:* 2015. *Vector species: Aedes aegypti*. *CHIKV lineage:* Asian. *CHIKV-naive:* Yes. *Available data:* 3,117.7 cases per 100,000 people (1,990 total).

HND-09: San Vicente Centenario, Honduras. *Study:* Zambrano et al. [93]. *Population size:* 3,673 individuals in 896 households. *Outbreak year:* 2015. *Vector species: Aedes aegypti*. *CHIKV lineage:* Asian. *CHIKV-naive:* Yes. *Available data:* 24,312.6 cases per 100,000 people (893 total).

IND-01: Adyanadka, India. *Study:* Manimunda et al. [62]. *Population size:* 13,861 individuals in 3,000 households. Map is a disk centered about the Adyanadka primary health center in Dakshina Kanada, Karnataka, India. *Outbreak year:* 2008. *Vector species: Aedes albopictus*. *CHIKV lineage:* IOL. *CHIKV-naive:* No. *Available data:* 2,001 cases over 240 days; peak incidence of 903 cases per month after 150 days. The symptomatic attack rate among 300 randomly selected households was 780/1,174. Among a subset of 360 residents from those

households, 234 tested positive for anti-CHIKV IgM, of whom 220 showed symptoms of chikungunya.

IND-02: Andrott Island, India. *Study:* Samuel et al. [42]. *Population size:* 11,621 individuals in 2,039 households. *Outbreak year:* 2006. *Vector species: Aedes albopictus*. *CHIKV lineage:* IOL. *CHIKV-naive:* No. *Available data:* 2,815 cases over 21 days; peak incidence of 1,152 cases per week after 10 days.

IND-03: Atmakur, India. *Study:* Uthappa et al. [132]. *Population size:* 3,839 individuals in 893 households. *Outbreak year:* 2013. *Vector species: Aedes albopictus*. *CHIKV lineage:* IOL. *CHIKV-naive:* No. *Available data:* 114 cases over 36 days among a random sample of 954 individuals; peak incidence after 6 days.

IND-04: Barpada, India. *Study:* Dwibedi et al. [90]. *Population size:* 1,732 individuals in 345 households. *Outbreak year:* 2007. *Vector species: Aedes albopictus*. *CHIKV lineage:* IOL. *CHIKV-naive:* No. *Available data:* 88 cases.

IND-05: Bavi, India. *Study:* Chopra et al. [133]. *Population size:* 1,450 individuals in 337 households. *Outbreak year:* 2006. *Vector species: Aedes albopictus*. *CHIKV lineage:* IOL. *CHIKV-naive:* No. *Available data:* 509 cases over 74 days.

IND-06: Bolagarh, India. *Study:* Dwibedi et al. [90]. *Population size:* 85,710 individuals in 19,643 households. *Outbreak year:* 2007. *Vector species: Aedes albopictus*. *CHIKV lineage:* IOL. *CHIKV-naive:* No. *Available data:* 80 cases.

IND-07: Dhamnagar, India. *Study:* Dwibedi et al. [90]. *Population size:* 112,699 individuals in 22,666 households. *Outbreak year:* 2007. *Vector species: Aedes albopictus*. *CHIKV lineage:* IOL. *CHIKV-naive:* No. *Available data:* 226 cases.

IND-08: Gowripet, India. *Study:* Kaur et al. [134]. *Population size:* 2,649 individuals in 679 households. *Outbreak year:* 2006. *Vector species: Aedes aegypti*. *CHIKV lineage:* IOL. *CHIKV-naive:* No. *Available data:* 575 cases over 51 days.

IND-09: Kalpeni Island, India. *Study:* Samuel et al. [42]. *Population size:* 4,681 individuals in 1,089 households. *Outbreak year:* 2006. *Vector species: Aedes albopictus*. *CHIKV lineage:* IOL. *CHIKV-naive:* No. *Available data:* 3,388 cases over 34 days; peak incidence of 800 cases per week after 14 days.

IND-10: Keluapali, India. *Study:* Dwibedi et al. [90]. *Population size:* 1,565 individuals in 340 households. *Outbreak year:* 2006. *Vector species: Aedes albopictus*. *CHIKV lineage:* IOL. *CHIKV-naive:* No. *Available data:* 70 cases.

IND-11: Mahakalapada, India. *Study:* Dwibedi et al. [90]. *Population size:* 116,454 individuals in 26,405 households. *Outbreak year:* 2006. *Vector species: Aedes albopictus*.

CHIKV lineage: IOL. *CHIKV-naive:* No. *Available data:* 752 cases.

IND-12: Mallela, India. *Study:* Kaur et al. [134]. *Population size:* 1,931 individuals in 471 households. *Outbreak year:* 2005. *Vector species: Aedes aegypti*. *CHIKV lineage:* IOL. *CHIKV-naive:* No. *Available data:* 242 cases over 112 days; peak incidence of 40 cases per week after 95 days. Among a sample of 100 randomly selected asymptomatic individuals, 15% tested positive for anti-CHIKV IgM.

IND-13: Odagaon, India. *Study:* Dwibedi et al. [90]. *Population size:* 109,529 individuals in 26,871 households. *Outbreak year:* 2007. *Vector species: Aedes albopictus*. *CHIKV lineage:* IOL. *CHIKV-naive:* No. *Available data:* 932 cases.

IND-14: Port Blair, India. *Study:* Manimunda et al. [40]. *Population size:* 136,000 individuals in 34,085 households. *Outbreak year:* 2006. *Vector species: Aedes albopictus*. *CHIKV lineage:* IOL. *CHIKV-naive:* No. *Available data:* 4,469 cases, but 86,000 symptomatic infections (suspected but not reported). Based on suspected cases, the outbreak lasted 214 days and reached its peak incidence after 92 days.

IND-15: Tura, India. *Study:* Khan et al. [135]. *Population size:* 67,169 individuals in 13,994 households. *Outbreak year:* 2010. *Vector species: Aedes albopictus*. *CHIKV lineage:* IOL. *CHIKV-naive:* No. *Available data:* 64 cases over 41 days; peak incidence after 32 days.

IDN-01: Bogor, Indonesia. *Study:* Laras et al. [55]. *Population size:* 43,340 individuals in 10,701 households. *Outbreak year:* 2002. *Vector species: Aedes aegypti*. *CHIKV lineage:* Asian. *CHIKV-naive:* No. *Available data:* 119 cases over 112 days; peak incidence of 21 cases per week after 91 days. Among 45 randomly selected asymptomatic individuals (18 in Kebon Pedes, 27 in Kedung Badak), eight individuals tested positive for anti-CHIKV IgM and 22 tested positive for anti-CHIKV IgG. The authors identified 52 affected households in the affected part of Bogor. Among these households, 38 had exactly one chikungunya case. Among 25 randomly selected asymptomatic residents of the 52 affected households, 12 tested positive for anti-CHIKV IgM and 13 tested positive for anti-CHIKV IgG.

IDN-02: Kali Jaya, Indonesia. *Study:* Laras et al. [55]. *Population size:* 26,672 individuals in 7,409 households. *Outbreak year:* 2002. *Vector species: Aedes aegypti*. *CHIKV lineage:* Asian. *CHIKV-naive:* No. *Available data:* 169 cases over 161 days; peak incidence of 20 cases per week after 91 days. Among 124 randomly selected asymptomatic individuals, 12 tested positive for anti-CHIKV IgM and 50 tested positive for anti-CHIKV IgG. The authors identified 91 affected households in the affected part of Kali Jaya. Among these households, 43 had three or more chikungunya cases. Among 21 randomly selected asymptomatic residents of the 91 affected households, four tested positive for anti-CHIKV IgM and 15 tested positive for anti-CHIKV IgG.

IDN-03: Nagasepaha, Indonesia. *Study:* Sari et al. [136]. *Population size:* 1,589 individuals in 454 households. *Outbreak year:* 2015. *Vector species: Aedes aegypti*. *CHIKV lineage:* Asian.

CHIKV-naive: No. *Available data:* 96 cases over 28 days; peak incidence of 53 cases per week after 20 days.

IDN-04: Sei Suka, Indonesia. *Study:* Sitepu et al. [137]. *Population size:* 54,773 individuals in 13,359 households. *Outbreak year:* 2013. *Vector species: Aedes albopictus. CHIKV lineage:* IOL. *CHIKV-naive:* No. *Available data:* 94 cases over 92 days; peak incidence of 12 cases per day after 46 days.

IDN-05: Sukadana, Indonesia. *Study:* Sitepu et al. [138]. *Population size:* 21,142 individuals in 4,917 households. *Outbreak year:* 2009. *Vector species: Aedes aegypti. CHIKV lineage:* Asian. *CHIKV-naive:* No. *Available data:* 65 cases over 29 days; peak incidence of 16 cases per two-day period after 21 days.

ITA-01: Anzio, Italy. *Study:* Vairo et al. [45]. *Population size:* 54,311 individuals in 28,585 households. *Outbreak year:* 2017. *Vector species: Aedes albopictus. CHIKV lineage:* IOL. *CHIKV-naive:* Yes. *Available data:* 182 cases over 132 days; peak incidence after 70 days.

ITA-02: Guardavalle Marina, Italy. *Study:* Riccardo et al. [139]. *Population size:* 2,346 individuals in 978 households. *Outbreak year:* 2017. *Vector species: Aedes albopictus. CHIKV lineage:* ECSA. *CHIKV-naive:* Yes. *Available data:* 132 cases over 89 days; peak incidence of eight cases per day after 52 days.

ITA-03: Castiglione di Ravenna and Castiglione di Cervia, Italy. *Study:* Rezza et al. [50]. *Population size:* 3,968 individuals in 1,890 households. *Outbreak year:* 2007. *Vector species: Aedes albopictus. CHIKV lineage:* IOL. *CHIKV-naive:* Yes. *Available data:* 161 cases over 85 days; peak incidence of 11 cases per day after 45 days.

LAO: Moonlapamok and Khong Districts, Lao People's Democratic Republic. *Study:* Soulaphy et al. [52]. *Population size:* 126,321 individuals in 22,557 households. *Outbreak year:* 2012. *Vector species: Aedes albopictus. CHIKV lineage:* IOL [140]. *CHIKV-naive:* Yes. *Available data:* 197 cases over 113 days; peak incidence of 13 cases per two-day period after 101 days.

MYS-01: Ipoh City, Malaysia. *Study:* Noridah et al. [141]. *Population size:* 16,598 individuals in 1,660 households. Map was a disk of radius 1,100 m centered about the part of Jalan Bendahara discussed in Noridah et al. [141]. *Outbreak year:* 2006. *Vector species: Aedes albopictus. CHIKV lineage:* IOL. *CHIKV-naive:* No. *Available data:* 52 cases.

MYS-02: Taman Kem, Malaysia. *Study:* Lam et al. [142]. *Population size:* 3,894 individuals in 742 households. *Outbreak year:* 1998. *Vector species: Aedes aegypti. CHIKV lineage:* Asian. *CHIKV-naive:* No. *Available data:* 51 cases over 84 days; peak incidence of four cases per day after 36 days.

NIC: District 2 of Managua, Nicaragua. *Study:* Gordon et al. [47]. *Population size:* 61,203

individuals in 10,902 households. *Outbreak year:* 2014; we only considered the first wave of chikungunya cases reported by Gordon et al. [47]. *Vector species: Aedes aegypti*. *CHIKV lineage:* Asian. *CHIKV-naive:* Yes. *Available data:* Among a sample of 3,837 children (modeled as randomly selected individuals), there were 95 cases over 195 days, with a peak incidence of 10 cases per week after 70 days. The entire cohort seems to have been tested for recent CHIKV infection using an IgM assay. Among the IgM-positive individuals, 45.5% reported symptomatic disease.

PHL: Concepcion, Philippines. *Study:* Ballera et al. [143]. *Population size:* 7,881 individuals in 1,970 households. *Outbreak year:* 2012. *Vector species: Aedes aegypti*. *CHIKV lineage:* Asian. *CHIKV-naive:* No. *Available data:* 98 cases over 44 days; peak incidence of 10 cases per two-day period after 27 days.

PNG: Vanimo-Green River District, Papua New Guinea. *Study:* Horwood et al. [53]. *Population size:* 70,531 individuals in 11,858 households. *Outbreak year:* 2012. *Vector species: Aedes albopictus*. *CHIKV lineage:* IOL. *CHIKV-naive:* Yes. *Available data:* 1,590 cases over 153 days; peak incidence of 213 cases per week after 84 days.

SGP: Sungei Kadut, Singapore. *Study:* Ng et al. [144]. *Population size:* 881 individuals in 187 households. *Outbreak year:* 2008. *Vector species: Aedes albopictus*. *CHIKV lineage:* IOL. *CHIKV-naive:* No. *Available data:* 85 cases.

LKA: Galagedara-Madige, Sri Lanka. *Study:* Kularatne et al. [145]. *Population size:* 1,001 individuals in 199 households. *Outbreak year:* 2006. *Vector species: Aedes albopictus*. *CHIKV lineage:* IOL. *CHIKV-naive:* No. *Available data:* 513 cases over 212 days; peak incidence of 182 cases per month after 61 days; 159 households had at least one case.

SXM: Saint Martin (whole island). *Studies:* Gay et al. [146] for the French side (Saint Martin) and Henry et al. [147] for the Dutch side (Sint Maarten).*Population size:* 78,410 individuals in 29,625 households. *Outbreak year:* 2013 (first known outbreak in the Americas). *Vector species: Aedes aegypti*. *CHIKV lineage:* Asian. *CHIKV-naive:* Yes. *Available data:* Because we combined data from separate studies on the French and Dutch sides of the island, we had access to a small amount of spatial data. On the French side, there were 5,487 cases over 547 days with a peak incidence of 358 cases per week after 112 days. On the Dutch side, there were 658 cases over 364 days.

THA-01: Khuan Don, Thailand. *Study:* Chaisongkram et al. [148] *Population size:* 26,814 individuals in 8,650 households. *Outbreak year:* 2018. *Vector species: Aedes aegypti*. *CHIKV lineage:* Asian. *CHIKV-naive:* No. *Available data:* 75 cases over 116 days; peak incidence after 84 days.

THA-02: Mueang Satun, Thailand. *Study:* Chaisongkram et al. [148] *Population size:* 115,641 individuals in 37,304 households. *Outbreak year:* 2018. *Vector species: Aedes aegypti*. *CHIKV

lineage: Asian. *CHIKV-naive:* No. *Available data:* 407 cases over 116 days; peak incidence after 84 days.

THA-03: Thung Nari, Thailand. *Study:* Nakkhara et al. [43]. *Population size:* 48,579 individuals in 13,150 households. *Outbreak year:* 2009. *Vector species: Aedes albopictus*. *CHIKV lineage:* ECSA. *CHIKV-naive:* No. *Available data:* Among 507 randomly selected individuals from 338 households, there were 166 cases over 729 days, with a peak incidence of 37 cases per month after 165 days. Within this sample of 507 individuals, 314 individuals tested positive for anti-CHIKV IgG.

VIR-01: Saint Croix, U.S. Virgin Islands. *Study:* Feldstein et al. [149]. *Population size:* 50,379 individuals in 23,173 households. *Outbreak year:* 2014. *Vector species: Aedes aegypti*. *CHIKV lineage:* Asian. *CHIKV-naive:* Yes. *Available data:* 143 cases over 245 days; peak incidence of 58 cases per month after 122 days.

VIR-02: Saint John, U.S. Virgin Islands. *Study:* Feldstein et al. [149]. *Population size:* 51,408 individuals in 27,054 households. *Outbreak year:* 2014. *Vector species: Aedes aegypti*. *CHIKV lineage:* Asian. *CHIKV-naive:* Yes. *Available data:* 469 cases over 214 days; peak incidence of 259 cases per month after 153 days.

VEN-01: San Jose, Venezuela. *Study:* Lizarazo et al. [41]. *Population size:* 137,579 individuals in 43,433 households. *Outbreak year:* 2014. *Vector species: Aedes aegypti*. *CHIKV lineage:* Asian. *CHIKV-naive:* Yes. *Available data:* 68 cases.

VEN-02: Santa Rosa, Venezuela. *Study:* Lizarazo et al. [41]. *Population size:* 73,130 individuals in 19,256 households. *Outbreak year:* 2014. *Vector species: Aedes aegypti*. *CHIKV lineage:* Asian. *CHIKV-naive:* Yes. *Available data:* 70 cases.

VEN-03: Tocuyito, Venezuela. *Study:* Lizarazo et al. [41]. *Population size:* 134,883 individuals in 35,809 households. *Outbreak year:* 2014. *Vector species: Aedes aegypti*. *CHIKV lineage:* Asian. *CHIKV-naive:* Yes. *Available data:* 70 cases.

FSM-01: Fais Island, Yap Islands, Federated States of Micronesia. *Study:* Pastula et al. [46]. *Population size:* 307 individuals in 68 households. *Outbreak year:* 2013. *Vector species: Aedes aegypti*. *CHIKV lineage:* Asian. *CHIKV-naive:* Yes. *Available data:* 136 cases over 38 days; peak incidence of 73 cases per week after 14 days.

FSM-02: Ifalik Atoll, Yap Islands, Federated States of Micronesia. *Study:* Pastula et al. [46]. *Population size:* 603 individuals in 86 households. *Outbreak year:* 2014. *Vector species: Aedes aegypti*. *CHIKV lineage:* Asian. *CHIKV-naive:* Yes. *Available data:* 88 cases over 49 days; peak incidence of 28 cases per week after 21 days.

FSM-03: Main Island, Yap Islands, Federated States of Micronesia. *Study:* Pastula et al. [46]. *Population size:* 7,586 individuals in 1,764 households. *Outbreak year:* 2013. *Vector species:*

Aedes aegypti. *CHIKV lineage:* Asian. *CHIKV-naive:* Yes. *Available data:* 1,412 cases over 343 days; peak incidence of 172 cases per week after 84 days.

YEM-01: Al Munirah, Yemen. *Study:* Malik et al. [54]. *Population size:* 44,361 individuals in 6,621 households. *Outbreak year:* 2010. *Vector species: Aedes aegypti*. *CHIKV lineage:* IOL. *CHIKV-naive:* Yes. *Available data:* 515 cases.

YEM-02: Alluheyah, Yemen. *Study:* Malik et al. [54]. *Population size:* 122,468 individuals in 18,279 households. *Outbreak year:* 2011. *Vector species: Aedes aegypti*. *CHIKV lineage:* IOL. *CHIKV-naive:* Yes. *Available data:* 95 cases.

YEM-03: As Salif, Yemen. *Study:* Malik et al. [54]. *Population size:* 7,503 individuals in 1,120 households. *Outbreak year:* 2010. *Vector species: Aedes aegypti*. *CHIKV lineage:* IOL. *CHIKV-naive:* Yes. *Available data:* 79 cases.

YEM-04: At Tuhayat, Yemen. *Study:* Malik et al. [54]. *Population size:* 81,999 individuals in 12,239 households. *Outbreak year:* 2010. *Vector species: Aedes aegypti*. *CHIKV lineage:* IOL. *CHIKV-naive:* Yes. *Available data:* 512 cases.

S2 Effects of each model parameter

Figures S1-S5 show how each model parameter (excluding R_0, R_e, and p_{detect}, which are functions of other parameters) affects R_0 and four common outbreak data types: attack rate, duration, peak incidence of new cases per day, and peak timing. These example simulations were run for the Char Kushai, Bangladesh, population (outbreak BGD-01). As each parameter was varied one-at-a-time, the remaining parameters were randomly sampled from their posterior distribution. Below, we summarize the effects of each parameter.

- Parameter B (expected transmissions per infected person) has a positive, nearly linear effect of R_0 (Figure S1). Outbreak size and peak incidence increase nonlinearly with B (Figures S2 and S4). Duration and the time of peak incidence vary non-monotonically with B, increasing sharply over lower values of B but slowly decreasing over larger values (Figures S3 and S5).
- Parameter C (transmission heterogeneity, i.e., the coefficient of variation of household-specific expected transmissions per infected person) has negative effects on R_0, outbreak size, outbreak duration, and peak incidence (Figures S1-S4), since house-to-house variability tends to break transmission chains prematurely. Peak timing generally decreases with C as well, but the simulated outbreaks with the latest peaks had intermediate values of C (Figure S5). This may reflect a trade-off where C is low enough that outbreaks do not

fade early and high enough that, if the outbreak reaches a house with unusually high transmission, a late burst of cases could occur.

- Parameter p_{immune} (proportion of population with pre-existing immunity) has no effect on R_0, since R_0 assumes a completely naive population (Figure S1). Greater immunity generally results in smaller, shorter outbreaks with lower, earlier peak incidence (Figures S2-S5). However, relatively high immunity (around 50%) produced the longest simulated outbreaks, probably by lowering $R_e = (1 - p_{\text{immune}})R_0$ to an intermediate value, such that the outbreak takes off but does not burn through the susceptible population too quickly.

- Parameters p_{asymp} and p_{report} (probabilities of asymptomatic infection and detecting a symptomatic infection, respectively) have no effect on R_0, which treats detected and undetected infections as equivalent (Figure S1). These parameters also have little to no effect on outbreak duration and peak timing (Figures S3 and S5). Total and peak daily cases (that is, detected infections) both decrease with p_{asymp} and both increase with p_{report} (Figures S2 and S4).

- Parameter p_{house} (fraction of transmissions received by members of an infected person's own household) has a negative relationship with R_0 and all four aspects of outbreak severity, to varying degrees (Figures S1-S5). This is because the more transmissions occur between members of the same household, the less CHIKV is transmitted to susceptible people throughout the population.

- Parameter G (mean generation interval length) has little effect on R_0 and total cases (Figures S1-S2). Longer generation intervals result in longer outbreaks and later times of peak incidence (Figures S3 and S5), but lower peak cases per day by spreading out cases over time (Figure S4).

- Parameter L (mean between-house transmission distance) has positive effects on R_0, outbreak size, peak incidence, and peak timing (Figures S1-S2 and S4-S5). The relationship between outbreak duration and L is more ambiguous, although it is not obvious why a shorter transmission distance would be associated with a longer outbreak duration (Figure S3).

- Parameter I_0 (initial number of infections) increases the probability that an outbreak will occur, but has little effect on the size and duration of the resulting outbreak (Figures S2-S3). Peak cases per day seems to increase slightly with I_0, probably due to multiple transmission chains unfolding at once (Figure S4). That peak tends to occur earlier as I_0 is increased (Figure S5). Since R_0 describes transmissions resulting from the introduction of a single infected person, it is not affected by I_0 at all (Figure S1).

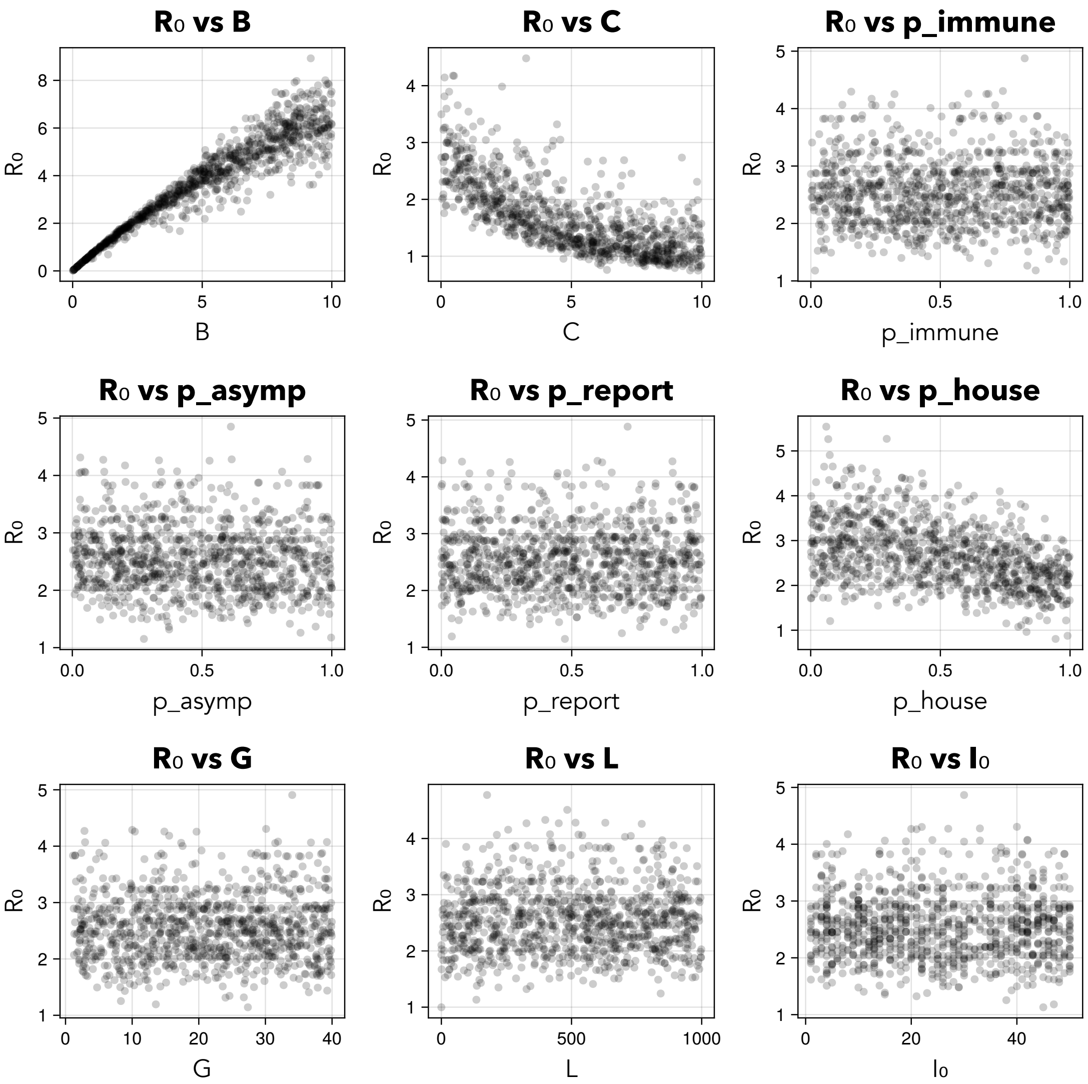

Figure S1: Relationship between R_0 and each model parameter. While each parameter was varied one-at-a-time, the other parameters were sampled from their posterior distribution.

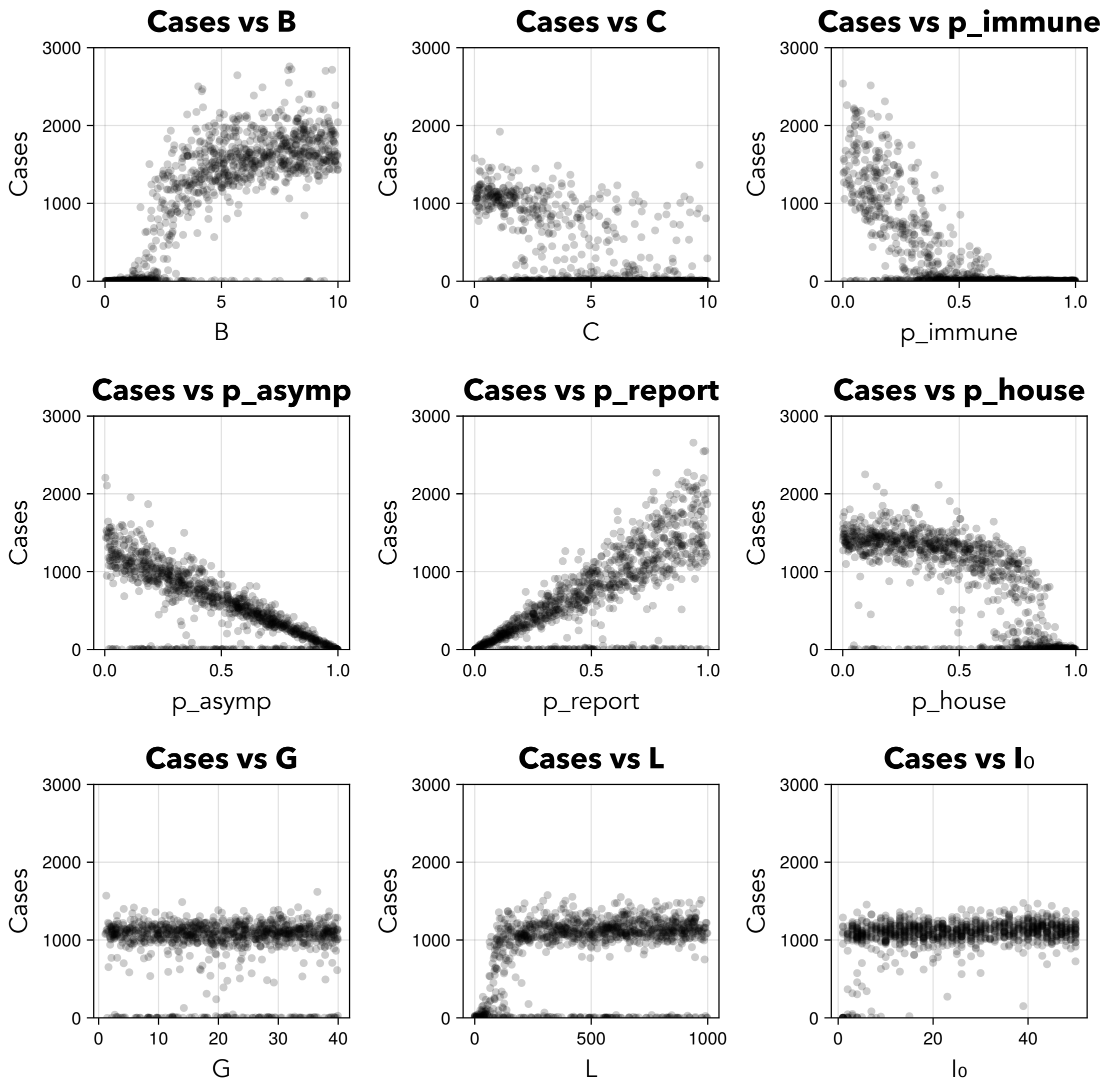

Figure S2: Relationship between chikungunya cases (reported symptomatic infections) and each model parameter and R_0. While each parameter was varied one-at-a-time, the other parameters were sampled from their posterior distribution. The dependence on R_0 was obtained from pooling these results from each individual parameter.

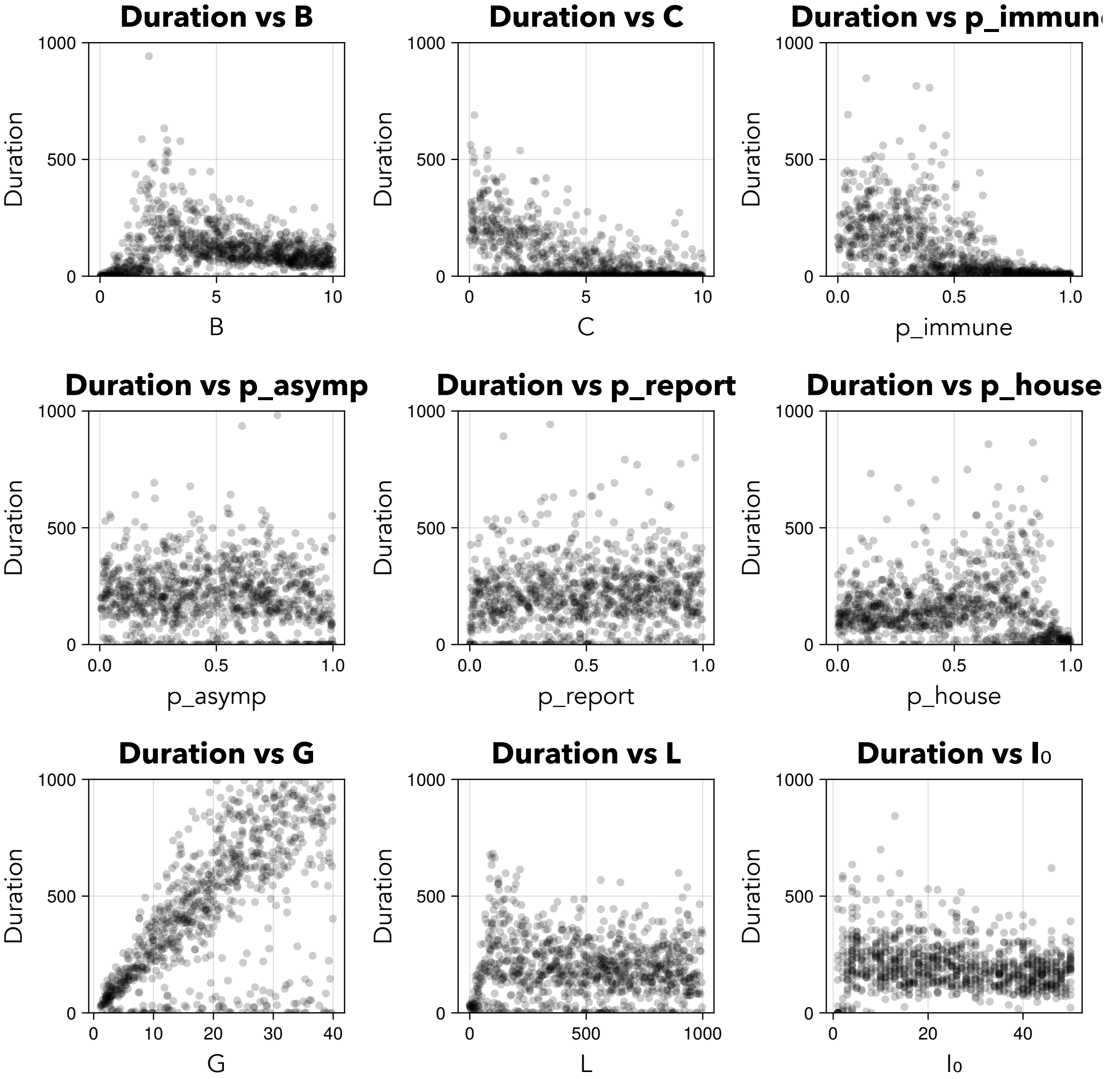

Figure S3: Relationship between outbreak duration and each model parameter and R_0. While each parameter was varied one-at-a-time, the other parameters were sampled from their posterior distribution. The dependence on R_0 was obtained from pooling these results from each individual parameter.

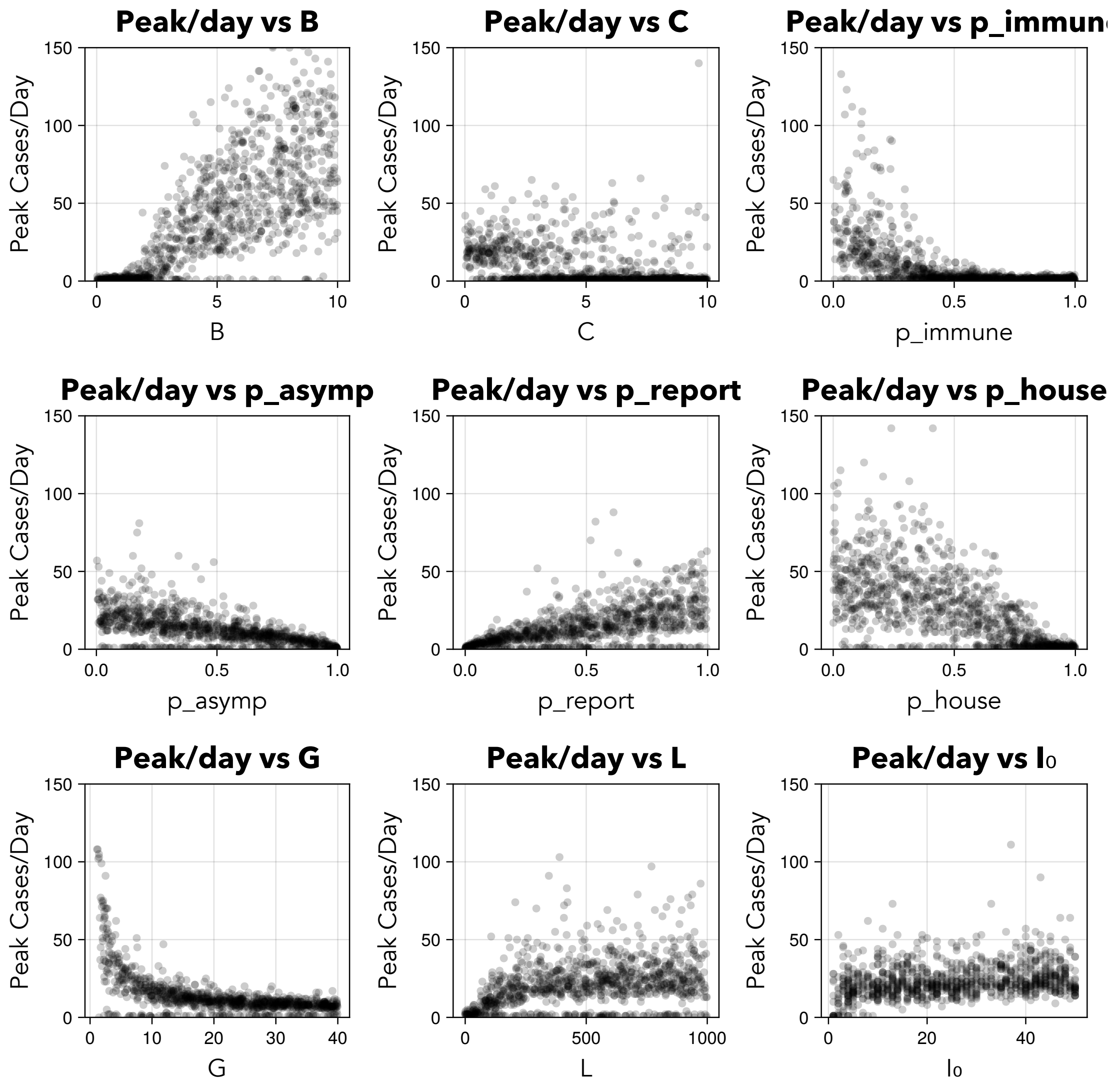

Figure S4: Relationship between peak incidence of chikungunya cases per day and each model parameter and R_0. While each parameter was varied one-at-a-time, the other parameters were sampled from their posterior distribution. The dependence on R_0 was obtained from pooling these results from each individual parameter.

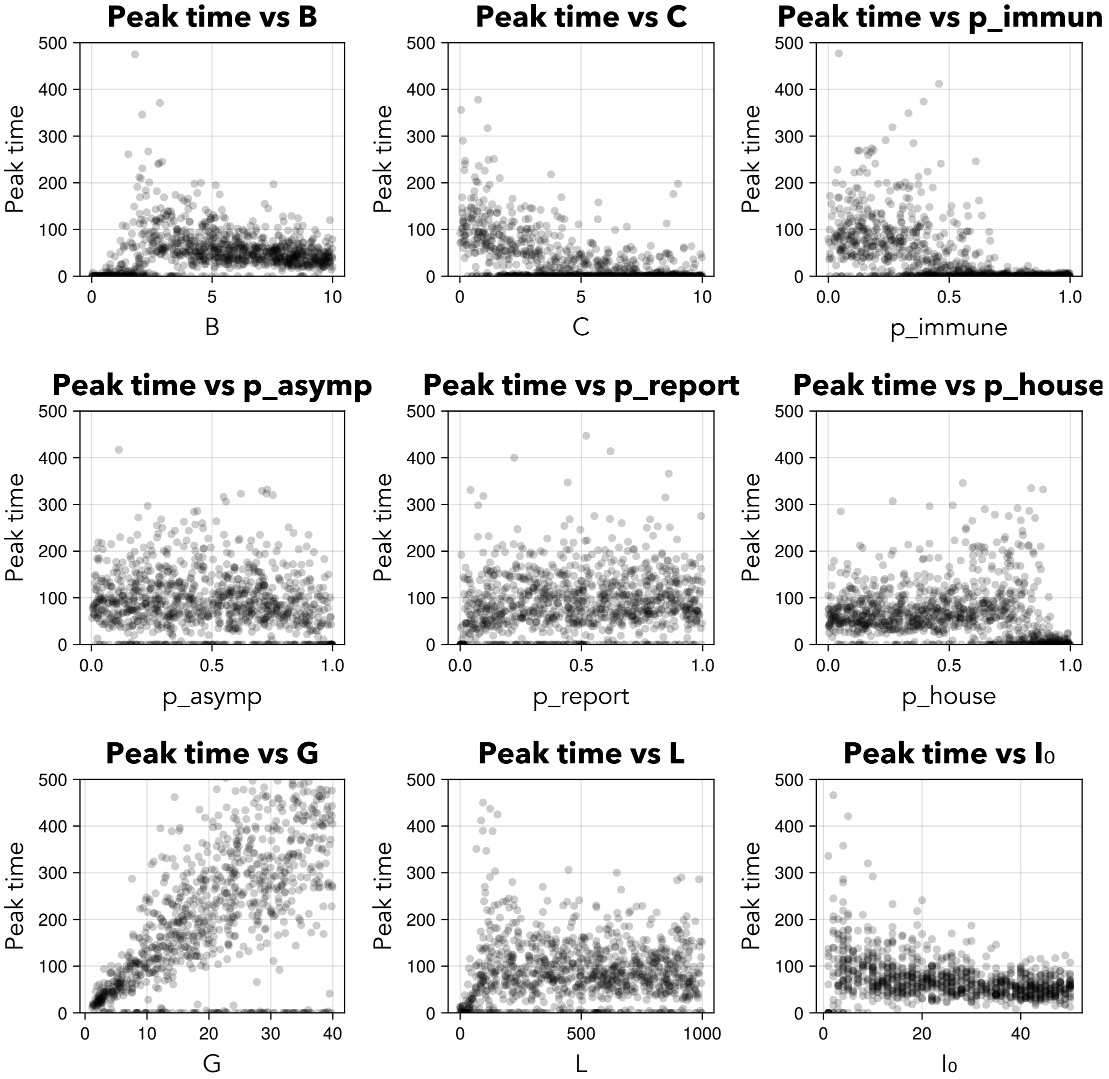

Figure S5: Relationship between the time of peak incidence and each model parameter and R_0. While each parameter was varied one-at-a-time, the other parameters were sampled from their posterior distribution. The dependence on R_0 was obtained from pooling these results from each individual parameter.

S3 Parameter Inference and Model Fit

S3.1 Parameter Priors

Estimating the CHIKSIM model parameters in main text **Table 1** for each outbreak required prior distributions for each parameter. These distributions are detailed below and summarized in **Table S1**.

Expected transmissions per infections, *B*. As mentioned in the main text, the quantity B is equivalent to R_0 for an infinite population with homogeneous mixing (i.e., the situation considered by most ordinary differential equation models). Thus, we fit a prior for B to published estimates of R_0 for CHIKV. Liu et al. [13] tabulated 20 estimates of R_0 for CHIKV. We discarded nine estimates from outbreaks to which we planned to fit the CHIKSIM model. Since we selected outbreaks with at least 50 cases, this biased our set of R_0 estimates downward; to compensate, we also discarded three estimates of R_0 that were below 1. To the remaining eight R_0 estimates, we fit several distributions (Normal, LogNormal, InverseGaussian, Weibull, Rayleigh), of which the Weibull distribution fit best (**Figure S6A**). This resulted in the prior

$$B \sim \text{Weibull}(3.21, 3.12). \tag{S1}$$

Transmission heterogeneity, *C*. Unlike R_0, transmission heterogeneity for CHIKV has not been measured extensively in the field. As in [38], we chose an Exponential distribution as a weak prior,

$$C \sim \text{Exponential}(2) \tag{S2}$$

(**Figure S6B**). The mean $C = 2$ results in transmission heterogeneity that matches the classic 80/20 rule, in which 80% of transmissions are caused by 20% of the infected population [106].

Pre-existing immunity, p_{immune}. Fritzell et al. [67] compiled estimates of anti-CHIKV seropositivity in populations around the world from 54 studies. Among these estimates, 39 were for populations to which we did not plan to fit the CHIKSIM model. We fit a Beta distribution to these estimates (**Figure S6C**), resulting in the prior

$$p_{\text{immune}} \sim \text{Beta}(0.81, 1.89). \tag{S3}$$

Probability of asymptomatic infection, p_{asymp}. [56] compiled 21 published estimates of the fraction of serologically confirmed CHIKV infections that are inapparent (asymptomatic or undocumented), and used them to propose prior distributions on the probability of asymptomatic infection for the Asian, ECSA, and IOL lineages of CHIKV. We refitted these priors, weighting studies by sample size and excluding seven serosurveys of populations included in our analysis. This resulted in the priors

$$p_{\text{asymp}} \sim \text{Beta}(4.09, 4.54) \tag{S4}$$

for the Asian lineage of CHIKV and

$$p_{\text{asymp}} \sim \text{Beta}(2.55, 10.63) \quad \text{(S5)}$$

for the ECSA and IOL lineages of CHIKV (**Figure S6D-E**). We used a single prior for both ECSA and IOL because our final sample contained no serosurveys of IOL to which to fit a separate prior, but [56] determined that these lineages have nearly equal probabilities of asymptomatic infection.

Probability that a symptomatic infection is reported, p_{report}. Reporting probabilities are notoriously variable across populations, pathogens, and studies [150]. We used the weakly informative prior

$$p_{\text{report}} \sim \text{Beta}(2, 2), \quad \text{(S6)}$$

which assumes that intermediate values are more likely for p_{report}, but any value between 0 and 1 is possible (**Figure S6F**).

Fraction of transmissions from an infected person received by members of their household, p_{house}. We derived a weak prior based on estimate for dengue virus (DENV), which is also transmitted by *Aedes aegypti* and *Aedes albopictus*. Cavany et al. [100] estimated, using an agent-based model that captured realistic human contact and mixing patterns, that the proportion of DENV transmissions received by members of the infected person's household is 0.54. Based on this, we chose the prior

$$p_{\text{house}} \sim \text{Beta}(2, 2), \quad \text{(S7)}$$

which (as noted above) assumes that intermediate values for p_{house} are most likely (**Figure S6G**).

Mean transmission distance, *L*. Studies by Guzzetta et al. [151] and Salje et al. [32] quantified the mean transmission distance of CHIKV, but used data from outbreaks included in our study. To avoid using these data twice, we constructed a prior on L using published estimates of the mean transmission distance of DENV. Broadly, research suggests that between-house transmission mediated by mosquito movement occurs on a lengthscale below 100 m, while human movement is responsible for transmission over greater distances [85, 152]. Specifically:

- Mammen Jr et al. [107] documented focal transmission of dengue in rural villages in Thailand. Fitting an Exponential distribution to the relationship between attack rate and distance from an index case (Figure 3 in that study) suggests a mean transmission distance of approximately 30 m.
- Vazquez-Prokopec et al. [108] noted that during a dengue outbreak in Cairns, Australia, cases during the first 5 weeks were clustered within 100 m of the index case. Following the authors assumption of a 20-day generation interval, this suggests a mean transmission distance of approximately 60 m.
- Stoddard et al. [85] suggested the distance 100 m as an ecologically meaningful lengthscale that separates mosquito- and human-mediated DENV transmission between households.

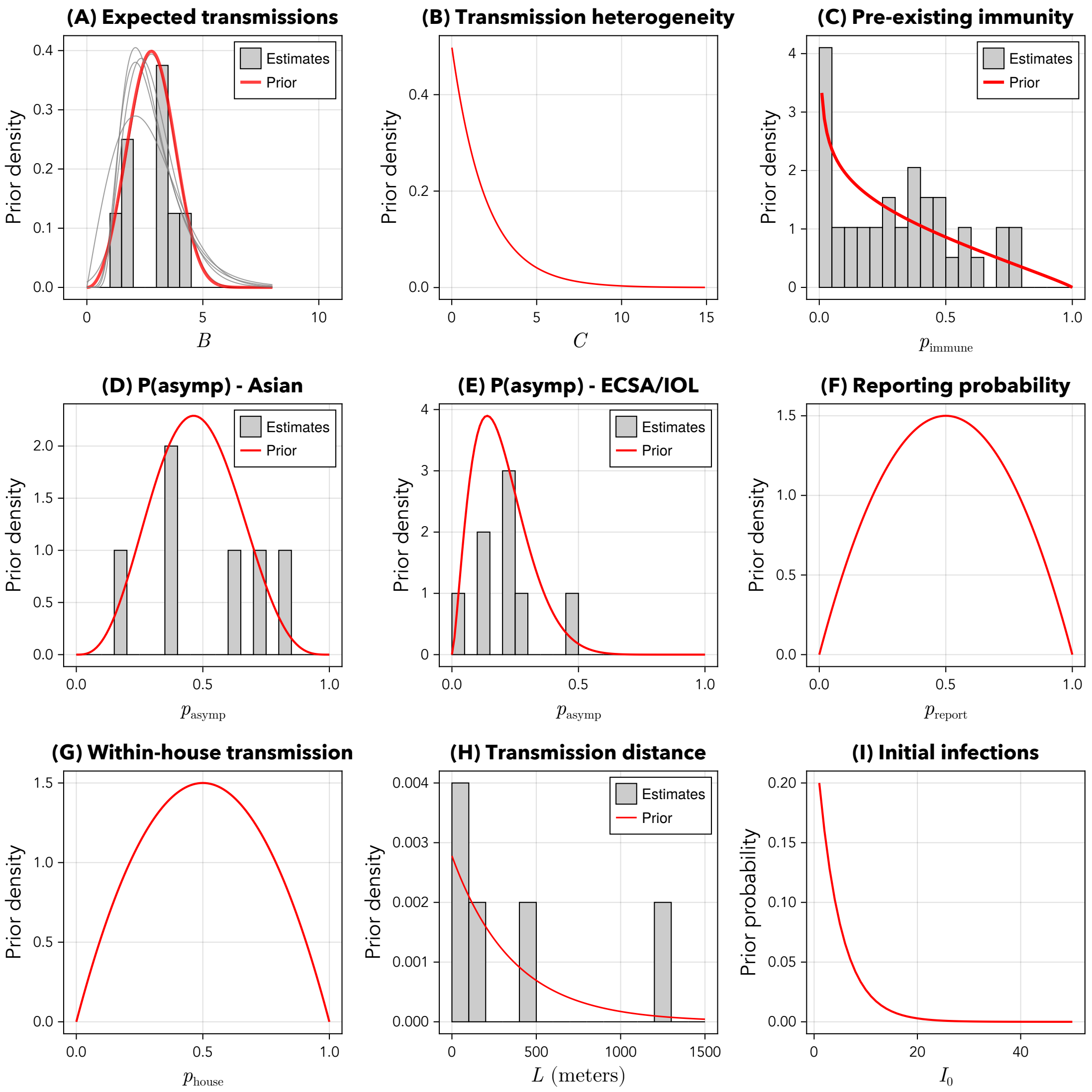

Figure S6: Priors for all CHIKSIM parameters besides the generation interval length. Red curves show the prior densities of each parameter, while gray histograms show any data used to inform those priors. In (A) the gray curves show alternative choices of distribution that did not fit the data as well as the Weibull.

- Guzzetta et al. [109] used genomic data to reconstruct a dengue transmission chain in Porto Alegre, Brazil. The authors quantified determined that 70% of transmissions took place over a distance less than 500 m. Assuming an Exponential distribution of transmission distances, this suggests a mean transmission distance of 410 m.

- Berry et al. [110] used genomic data to reconstruct between-house transmissions of dengue in Kamphaeng Phet, Thailand. Averaging the transmission distances presented in Table 1 of

that study results in the mean transmission distance 1210 m.

Fitting an Exponential distribution to these five estimates results in the prior

$$L \sim \text{Exponential}(360) \tag{S8}$$

with mean 360 m (**Figure S6H**).

Initial infections, I_0. To account for the possibility of multiple CHIKV infections being present in each population before the first chikungunya case is detected, we used the prior

$$I_0 \sim 1 + \text{Geometric}(1/5), \tag{S9}$$

a Geometric distribution that excludes $I_0 = 0$ and has mean 5 (**Figure S6I**).

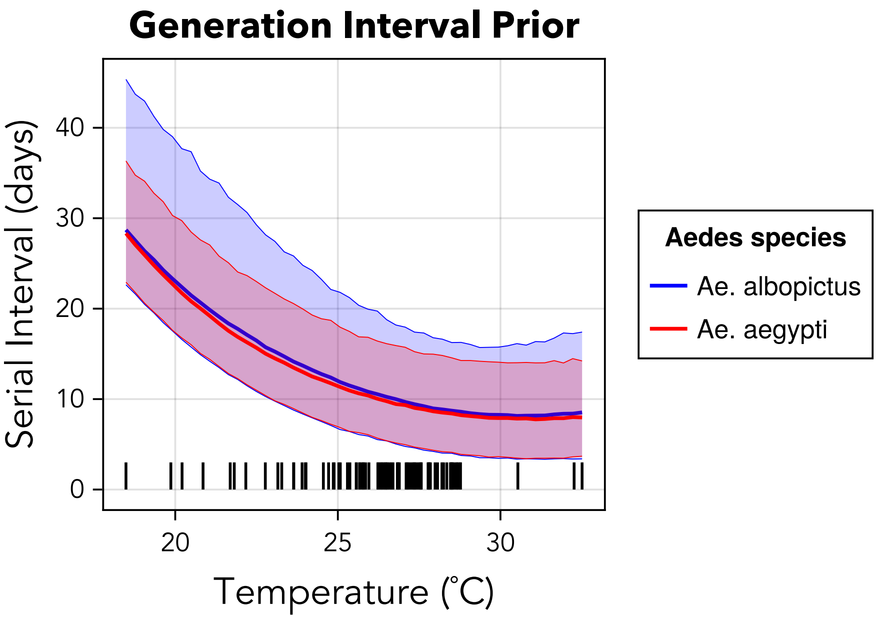

Figure S7: Median (lines) and 95% probability intervals (bands) for the generation interval G as a function of temperature and *Aedes* vector species. The tick marks along the horizontal axis show the mean temperatures during the first months of the 86 chikungunya outbreaks considered in this study.

Mean generation interval length, G. Previous studies have proposed qualitative relationships between climate and R_0, and testing some of those relationships is a goal of this study. In contrast, quantitative relationships between temperature and the generation intervals (or closely related serial intervals) are established for several *Aedes aegypti*-transmitted viruses [57, 59, 60, 61]. As our prior for the generation interval G (the duration between index and secondary infections), we

used the temperature-dependent distribution of the serial interval S (the duration between the onsets of the index and secondary infections' symptoms) for CHIKV derived by Riou et al. [57]. However, we modified the authors' argument to account for differences between *Aedes aegypti* and *Aedes albopictus*.

Riou et al. [57] decomposed the serial interval into the sum of four terms,

$$S = -t_v + t_b + t_m(T,A) + t_i, \tag{S10}$$

where T denotes temperature (degrees Celsius), A denotes vector species (*Ae. aegypti* or *Ae. albopictus*) and t_v, t_b, t_m, and t_i denote the lengths (in days) of different stages of the serial interval:

- t_V = time from the index infection becoming infectious to presenting symptoms. Riou et al. [57] used used $t_v \sim \text{Uniform}(0,3)$ for CHIKV.
- t_b = time from index case becoming infectious to index case transmitting CHIKV to a mosquito. Riou et al. [57] assumed that infectious duration for CHIKV lasts for $\tau \sim \text{Uniform}(3,8)$ and human-to-mosquito transmission could occur at any moment during that duration, $t_b|\tau \sim \text{Uniform}(0,\tau)$.
- $t_m(T,A)$ = time from mosquito becoming infected to mosquito transmitting CHIKV to a human secondary infection. This is the temperature- and species-dependent component of the serial interval distribution. First, $t_m(T,A)$ depends on the extrinsic incubation period (EIP, the time from when the mosquito becomes infected to when it becomes infectious), κ. Differences in the EIP of CHIKV across *Aedes* vector have not been clearly documented. Therefore, we used the same *Aedes aegypti*-derived expression as Riou et al. [57],

$$\kappa(T) = 3\exp[-0.21(T-28)]. \tag{S11}$$

Second, $t_m(T,A)$ depends on the length of the mosquito's gonotrophic cycle, $\gamma(T,A)$. Casas-Martínez et al. [111] found that, on average, the gonotrophic cycle of *Aedes albopictus* is 0.5 d shorter than that of *Ae. aegypti*. Therefore, we slightly modified the expression used by Riou et al. [57] to obtain

$$\gamma(T,A) = 56.6 - 3.74T + 0.064T^2 - 0.5 \cdot \mathbf{1}_{A=albopictus}. \tag{S12}$$

Third, $t_m(T,A)$ depends on the number of gonotrophic cycles $C(T,A)$ that elapse between the end of the EIP and when the mosquito transmits CHIKV to a human host. Let

$$s(T,A) = \exp[-\delta(A)\gamma(T,A)]$$

denote the probability that a mosquito survives one gonotrophic cycle. Here, $\delta(A)$ is the daily mortality rate of the mosquito; based on [58], we used $\delta = 0.29$ for *Ae. aegypti* and

$\delta = 0.12$ for *Ae. albopictus*. Riou et al. [57] assumed that the probability of the mosquito first transmitting CHIKV during its cth gonotrophic cycle was proportional to s^c.

We modified this logic slightly to be rigorous and account for the *Aedes* species' different transmission efficiencies. Let $p(A)$ be the probability that an infectious mosquito of species A transmits CHIKV during a gonotrophic cycle. For this value, we used estimates of the probability of an infectious mosquito transmitting CHIKV (that is, transmission efficiency) derived from [153, 154], and obtained the value $p = 0.6$ for *Ae. aegypti* and $p = 0.4$ for *Ae. albopictus*. The probability that the mosquito transmits CHIKV during its cth gonotrophic cycle (but not sooner) is proportional to $s^c(1-p)^{c-1}p$. This implies that the gonotrophic cycle during which the mosquito transmits follows the Geometric distribution

$$C(T,A) \sim 1 + \text{Geometric}(f(T,A)) \tag{S13}$$

which excludes 0 and has failure (i.e., surviving but not transmitting) probability

$$f(T,A) = s(T,A)[1 - p(A)].$$

Finally, we combined these results with the form for t_m proposed by Riou et al. [57] to obtain

$$t_m|C(T,A) \sim \kappa(T) + \gamma(T,A) \cdot \text{Uniform}\left(C(T,A) - \frac{1}{3}, C(T,A) + \frac{1}{3}\right). \tag{S14}$$

- t_i = the incubation period from infection (*not* infectiousness) until the onset of symptoms in the secondary infection. Riou et al. [57] used

  $$t_i \sim \text{LogNormal}(1.01, 0.41), \tag{S15}$$

  which has mean 3 d and standard deviation 1.3 d.

The resulting prior distributions for G are shown in **Figure S7** as functions of temperature for each vector species.

S3.2 Posterior estimates of all parameters

Due to space constraints, main text **Figure 2** only shows the individual outbreaks' posterior estimates of R_0. Figures S8-S9 show posterior estimates of the remaining parameters for each outbreak.

Table S1: Parameter priors.

Parameter	Prior	Source
B	$\text{Weibull}(3.21, 3.12)$	[13]
C	$\text{Exponential}(2)$	Assumed
p_{immune}	Naive: 0<br>Not naive: $\text{Beta}(0.81, 1.89)$	[67]
p_{asymp}	Asian: $\text{Beta}(4.09, 4.54)$<br>ECSA/IOL: $\text{Beta}(2.55, 10.63)$	[56]
p_{report}	$\text{Beta}(2, 2)$	Assumed
p_{house}	$\text{Beta}(2, 2)$	[100]
L	$\text{Exponential}(360)$	[85, 108, 107, 110, 109]
I_0	$1 + \text{Geometric}(1/5)$	Assumed
G	Temperature/species-dependent; see text for details.	[57]

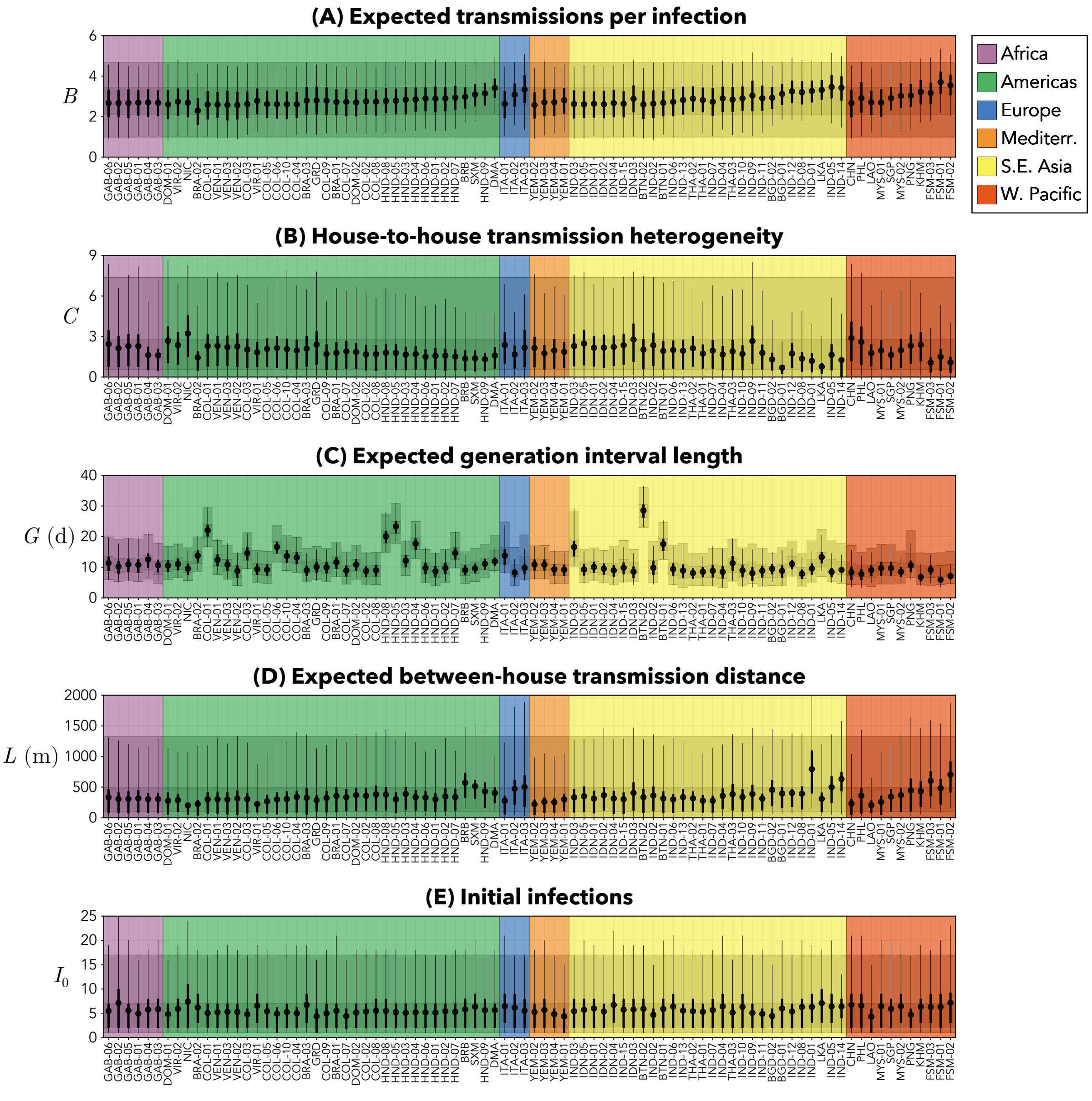

Figure S8: Posterior parameter estimates for individual outbreaks, part 1. Shaded regions show prior interquartile ranges (darker) and 95% probability intervals (lighter). Note the use of different priors for the generation interval length, G, for each outbreak, depending on vector species and initial temperature.

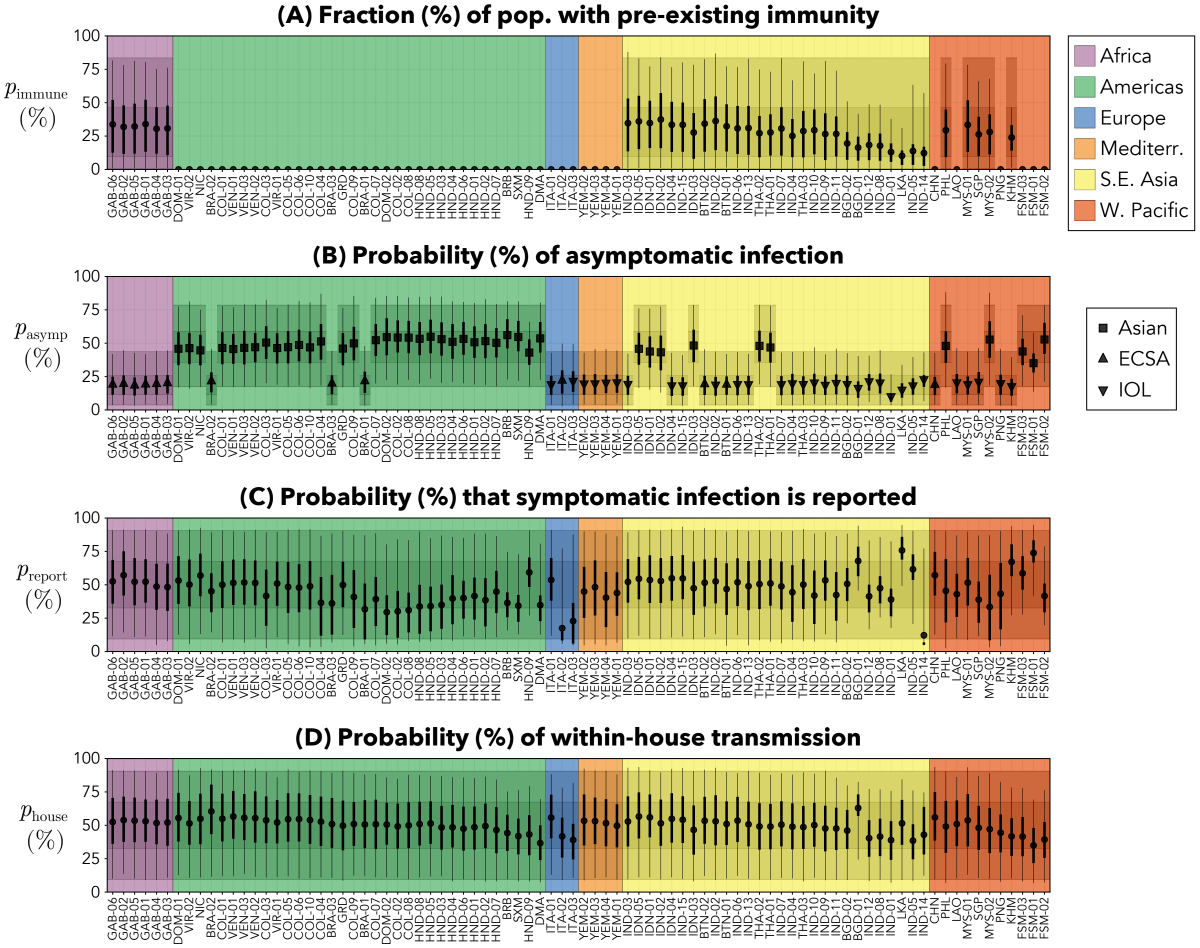

Figure S9: Posterior parameter estimates for individual outbreaks, part 2. Shaded regions show prior interquartile ranges (darker) and 95% probability intervals (lighter). Note the use of different priors for p_{immune} in outbreak sites considered to be naive (row 1), and for p_{asymp} for outbreaks of each CHIKV lineage (row 2, with different markers for each lineage).

S3.3 Experiments with Simulated Data

S3.3.1 Quality of the parameter estimates: coverage, bias, and concordance

To test the extent to which the parameters above were estimable from the available data, we applied our parameter inference method to outbreaks simulated with the CHIKSIM model using known parameters. For each real outbreak, we simulated 100 datasets with parameters drawn from their prior distributions (8,600 simulated datasets in total). We discarded simulations with fewer than 50 chikungunya cases so that these simulated data would be similar in magnitude to the real data. We used same procedure described in the main text to compute posterior distributions for each parameter from each simulated dataset. Finally, we used the known and estimated parameters for each outbreak's 100 simulated datasets to compute three metrics describing the quality of our estimates (three metrics per parameter per real outbreak, for a total of 2,322 values). To define these metrics, let θ_{ij} denote the true value of parameter θ for simulated dataset $j = 1, \ldots, 100$ for outbreak $i = 1, \ldots, 86$, let $\hat{\theta}_{ijk}$ be the kth draw from that parameter's posterior distribution, and let $\bar{\theta}_{ij}$ denote the parameter's posterior mean. Then:

- **Coverage** is the probability that the posterior credible intervals of a parameter for an outbreak contain the parameter's true value. Using 95% credible intervals, coverage probabilities should be close to 0.95. If $\hat{\theta}_{ij}^{\alpha}$ denotes the αth percentile of the posterior estimate of θ for outbreak i's simulated dataset j, then coverage is

$$\text{Coverage}(i, \theta) = \frac{1}{100} \sum_{j=1}^{100} \mathscr{I}\{\hat{\theta}_{ij}^{2.5} \leq \theta_{ij} \leq \hat{\theta}_{ij}^{97.5}\}. \tag{S16}$$

- **Bias** is the tendency for a parameter's true value to be over-/underestimated by its posterior estimates. Here, we compute bias as the mean signed relative error between the parameter's posterior means and true values. The bias of our estimates of θ for outbreak i is defined

$$\text{Bias}(i, \theta) = \frac{1}{100} \sum_{j=1}^{100} \frac{\bar{\theta}_{ij} - \theta_{ij}}{\theta_{ij}}. \tag{S17}$$

  These values should be close to 0, and positive (negative) values indicate a tendency to overestimate (understimate) the true value of θ.

- **Accuracy** is the extent to which a parameter's posterior estimates agree with the parameter's true values. Here, we define accuracy as the concordance correlation coefficient (or simply "concordance") between the parameter's true values and posterior means. Concordance modifies the standard Pearson correlation coefficient to measure the agreement between these sets of values (along $y = x$), rather than any other positive linear relationship (along $y = mx + b$ with $m > 0$) [155]. Let ρ_i denote the Pearson correlation

between θ_{ij} and $\bar{\theta}_{ij}$, σ_i and $\bar{\sigma}_i$ denote the standard deviations of θ_{ij} and $\bar{\theta}_{ij}$ (respectively), and μ_i and $\bar{\mu}_i$ denote the means of θ_{ij} and $\bar{\theta}_{ij}$ (respectively). The concordance of the posterior mean estimates of θ for outbreak i is

$$\text{Concordance}(i,\theta) = \left(\frac{2\sigma_i\bar{\sigma}_i}{\sigma_i^2 + \bar{\sigma}_i^2 + (\mu_i - \bar{\mu}_i)^2}\right) \cdot \rho_i. \tag{S18}$$

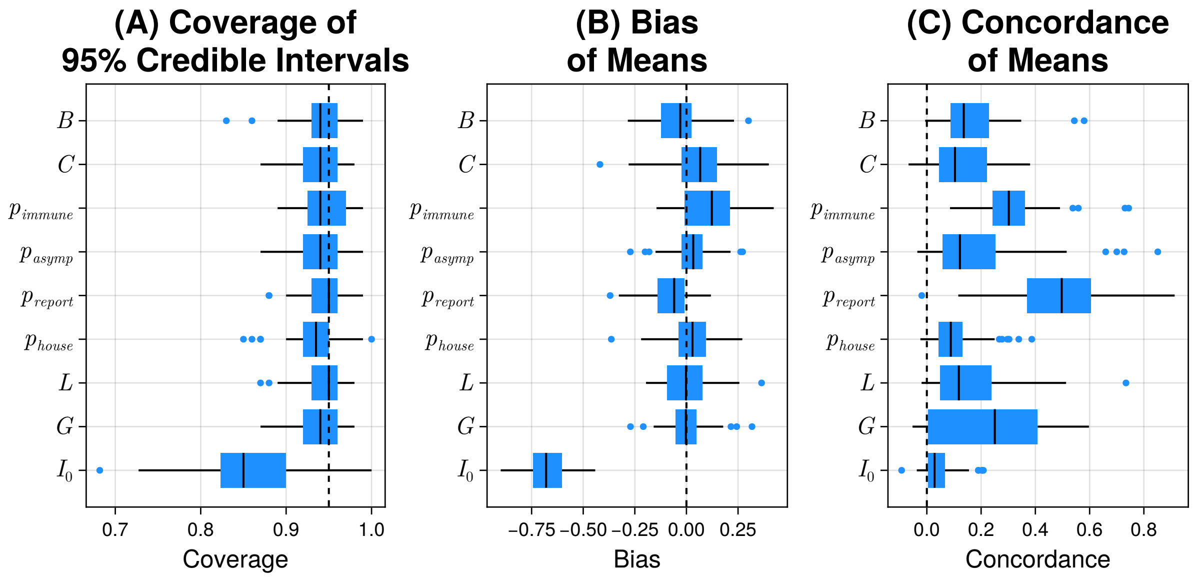

Figure S10: Parameter inference performance scores. Each boxplot shows the median (line), interquartile range (box), and outliers (dots) of coverage, bias, or concordance across the 86 outbreaks.

S3.3.2 Relationship between available data and quality of parameter estimates

As previously stated, we estimated epidemiological parameters for chikungunya outbreaks by fitting the CHIKSIM model to a small number of summary statistics available from each outbreak. As shown in Main Text **Figure 1B**, the 86 outbreaks varied in the types of data that were available. For example, while all outbreaks had an attack rate estimate, there were 38 outbreaks for which that was the only datum available. An estimate of outbreak duration was available for about half of all outbreaks, and only a small number had summary statistics regarding serology or the spatial distribution of cases. The mean number of summary statistics available per outbreak was 2.6, and the maximum number for a single outbreak was seven statistics.

Illuminating how the number and types of available summary statistics per outbreak affects the estimability of epidemiological parameters could be the topic of an entirely separate paper.

However, we were able to gain insight on this question using our inferences on the simulated datasets described above. This analysis is limited, in that it does not include combinations of summary statistics that did not appear among the 86 real outbreaks. For example, because only studies that gathered multiple summary statistics included serological or household-level data, it is difficult to separate the effects of these specific data types with those of adding any additional ones. Nonetheless, this analysis generated several useful insights.

Whenever an outbreak had only one available summary statistic, that statistic was the attack rate. Since the same observed attack rate can result from a wide range of parameters (for instance, high R_e with a low case reporting probability, or vice versa), adding just one additional summary statistic significantly improved the accuracy (as measured using concordance) of our estimates of most parameters (**Figures S11** and **S12**). Since the second-most commonly included statistic was outbreak duration, this effect is particularly pronounced for the generation interval length, G (**Figure S12**H). It was also clear for the fraction of the population with pre-existing immunity, p_{immune} (**Figure S12C**), suggesting that even coarse outbreak features such as duration can help distinguish between intense transmission (due to high R_e) with poor reporting, or the opposite.

Serological data appeared particularly valuable for estimating several parameters, although this may also reflect a correlation between the number of summary statistics used and the inclusion of a serological statistic. Nonetheless, estimates of the probability of asymptomatic infection, p_{asymp}, and the probability that a symptomatic infection is reported, p_{report}, were markedly more accurate for outbreaks with serological data (**Figure S12D,E**). This should translate to more accurate estimates of the probability of any infection being detected, $p_{\text{detect}} = (1 - p_{\text{asymp}})p_{\text{report}}$, as well. Finally, data on the distribution of cases across household were useful for estimating parameters governing the spatial dynamics of CHIKV transmission, including transmission heterogeneity across households, C, and the mean between-house transmission distance, L (**Figure S12B,G**). Household-level data also slightly improved our estimates of the fraction of transmissions that occur between members of the same household, p_{house}, although this parameter was estimated quite poorly overall (**Figure S12**).

Figure S11 seems to suggest that including more summary statistics results in better estimates of most parameters. However, as noted in Meyer et al. [38], there is a trade-off to using more summary statistics: the more summary statistics included, the less likely a model simulation is to match all statistics at once—and, therefore, the more simulations needed during parameter inference. This is a core issue in approximate Bayesian computation, and the purpose of using summary statistics in the first place [101]. More work is needed to determine the optimal number and types of summary statistics for estimating the parameters underlying infectious disease outbreaks. However, “optimality” most likely includes researchers’ unique constraints on time and computational resources.

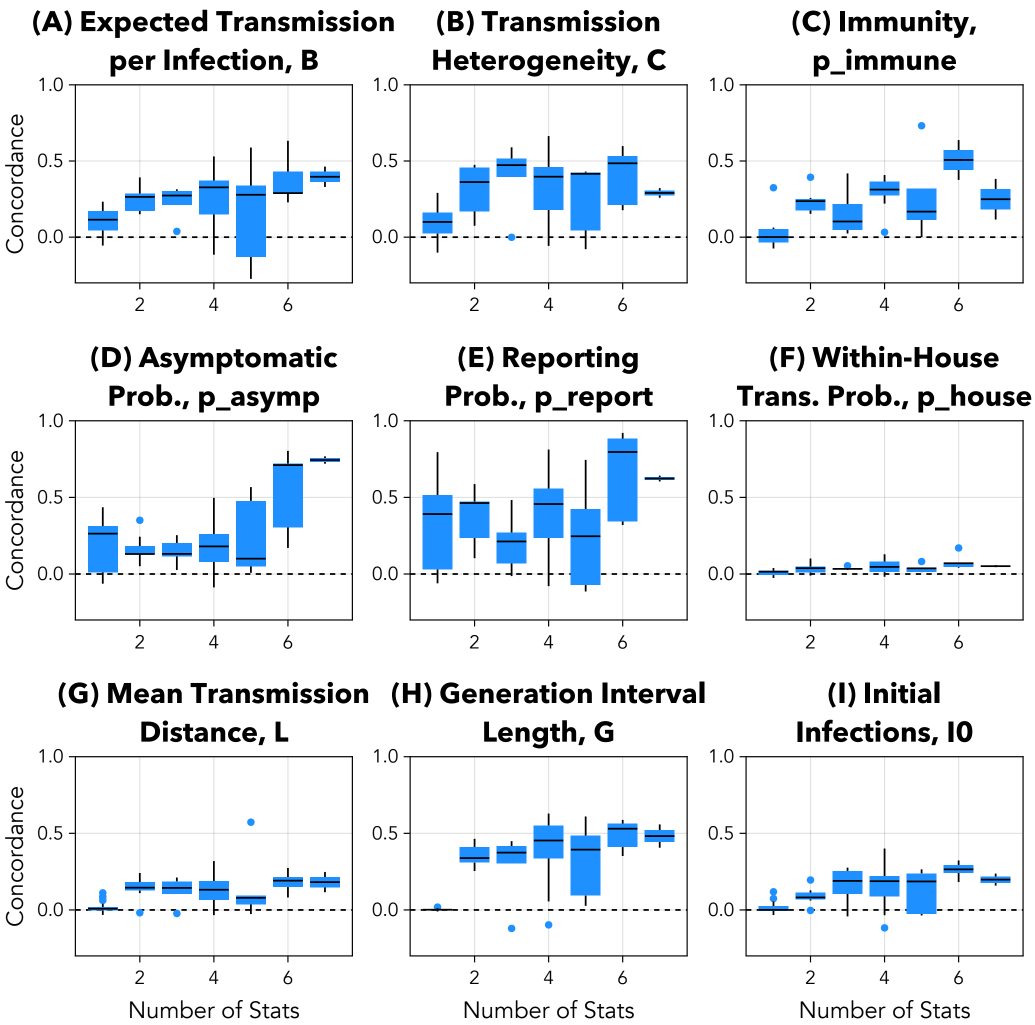

Figure S11: Impact of the number of summary statistics available for an outbreak on the accuracy of each parameters' estimates, as measured using the concordance of the posterior means with the true parameter values.

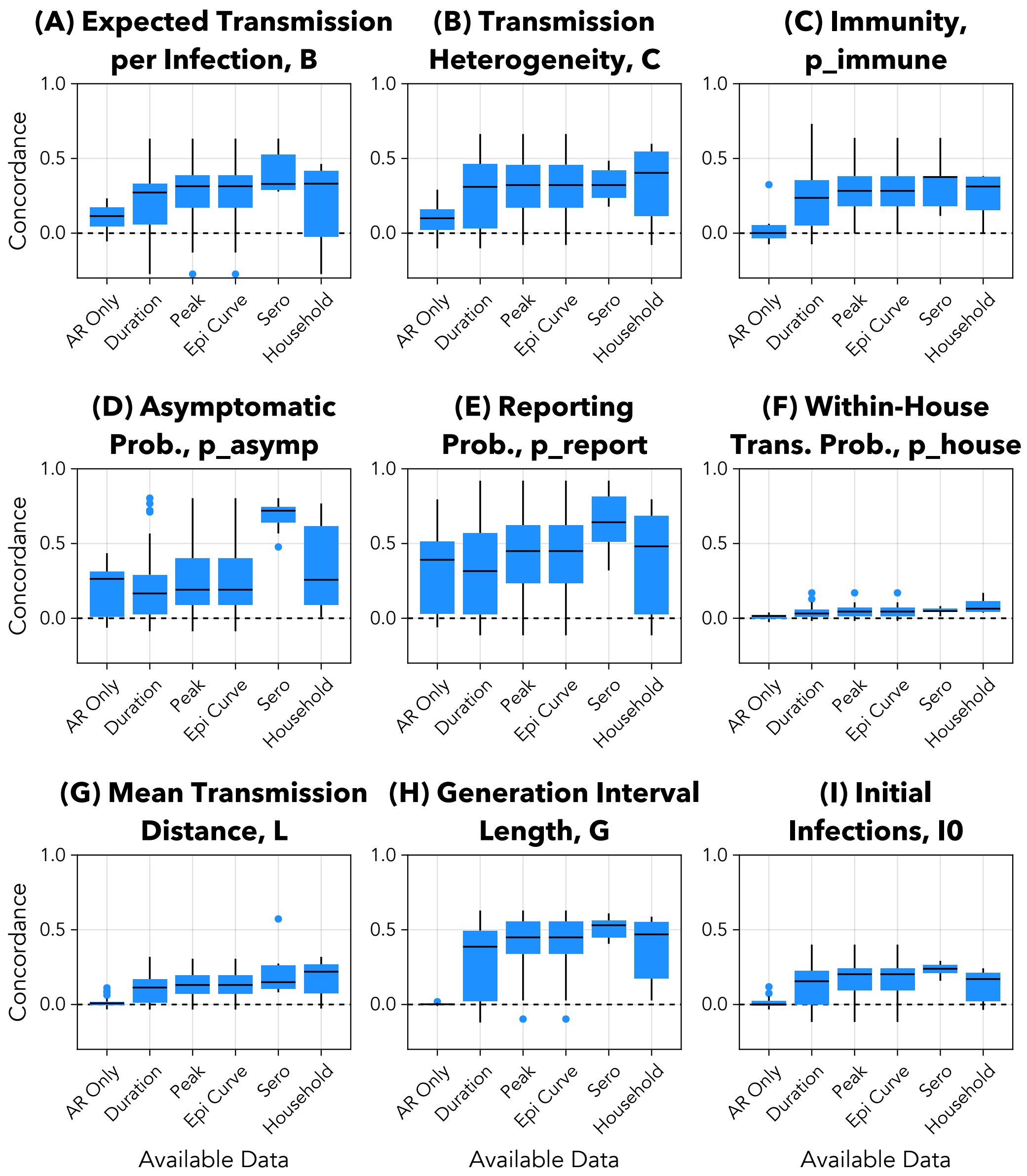

Figure S12: Impact of the types of summary statistics available for an outbreak on the accuracy of each parameters' estimates, as measured using the concordance of the posterior means with the true parameter values.

S3.4 Model fit to the available data

To verify that the CHIKSIM model captures the dynamics of real chikungunya outbreaks, we compared the models' posterior predictions against the real data available from each outbreak. **Figure S13** shows the agreement between the data and posterior predictive distributions of the four most common outbreak summary statistics: attack rate, outbreak duration, peak incidence, and the time at which the peak incidence was attained. For these statistics, we observed strong correlations between the observed values and mean posterior predictions. Model simulations were more likely to underestimate these quantities than to overestimate them. However, the observed values of these quantities were nearly always contained within the model's 95% prediction intervals. This suggests that each dataset was one of many plausible outcomes for each outbreak, based on the model. Perfect agreement between posterior means and the data is not necessary, since we do not expect all outbreaks to have unfolded in the most likely way possible given its underlying parameters.

Prior and posterior predictive distributions for all outbreak summary statistics are shown in **Figures S15-S21**. To visualize the predictions for each outbreak's data types (which often had different units and magnitudes) in single plots, we normalized the predictions $\hat{x}_k$ for each data type x by that datum's true value, $\hat{x}_k \mapsto (\hat{x}_k - x)/x$. After normalization, a prediction of 0 indicates agreement with the data. Across all 228 summary statistics fitted to all outbreaks, there were only four that did not fall within CHIKSIM's 95% posterior prediction intervals (highlighted in red in **Figures S15-S21**)—well below the 5% expected to occur through random chance. Among these four summary statistics were two observations that were unusual even among real chikungunya outbreaks: the delayed peak of the outbreak in Chapada, Brazil (commented on in the main text), and the extremely high attack rate (72%) of the outbreak in Kalpeni, India. Besides these, most summary statistics' true values were supported quite well by their posterior predictive distributions.

Finally, there were 19 outbreaks for which epidemic curves showing chikungunya incidence over time were available. These epidemic curves are compared against posterior simulations using functional boxplots (a generalization of the standard boxplot to timeseries data [156]) in **Figure S14**. For nearly all outbreaks, the observed epidemic curve (black) was of similar magnitude to the median simulation (red), and was well contained within the region occupied by most simulations (gray). The model fit was particularly good for the epidemic curve in Dominica. The model was less successful at recreating epidemic curves for outbreaks that peaked late relative to their size, including those in Palpara, Bangladesh; Trapeang Roka, Cambodia; and Chapada, Brazil. This disagreement could reflect seasonal forcing or interventions applied during those outbreaks, which were not included in the model. The simulated timeseries did not successfully fit the outbreaks in Franceville, Gabon, and Kalpeni and Andrott, India. Compared with other outbreaks' epidemic curves, these datasets were unique in having exceptionally high chikungunya incidence during the first week of each outbreak: nearly 100 in Franceville, nearly 600 in Kalpeni, and nearly 1,000 in Andrott. It is plausible that CHIKV circulated undetected for some time in

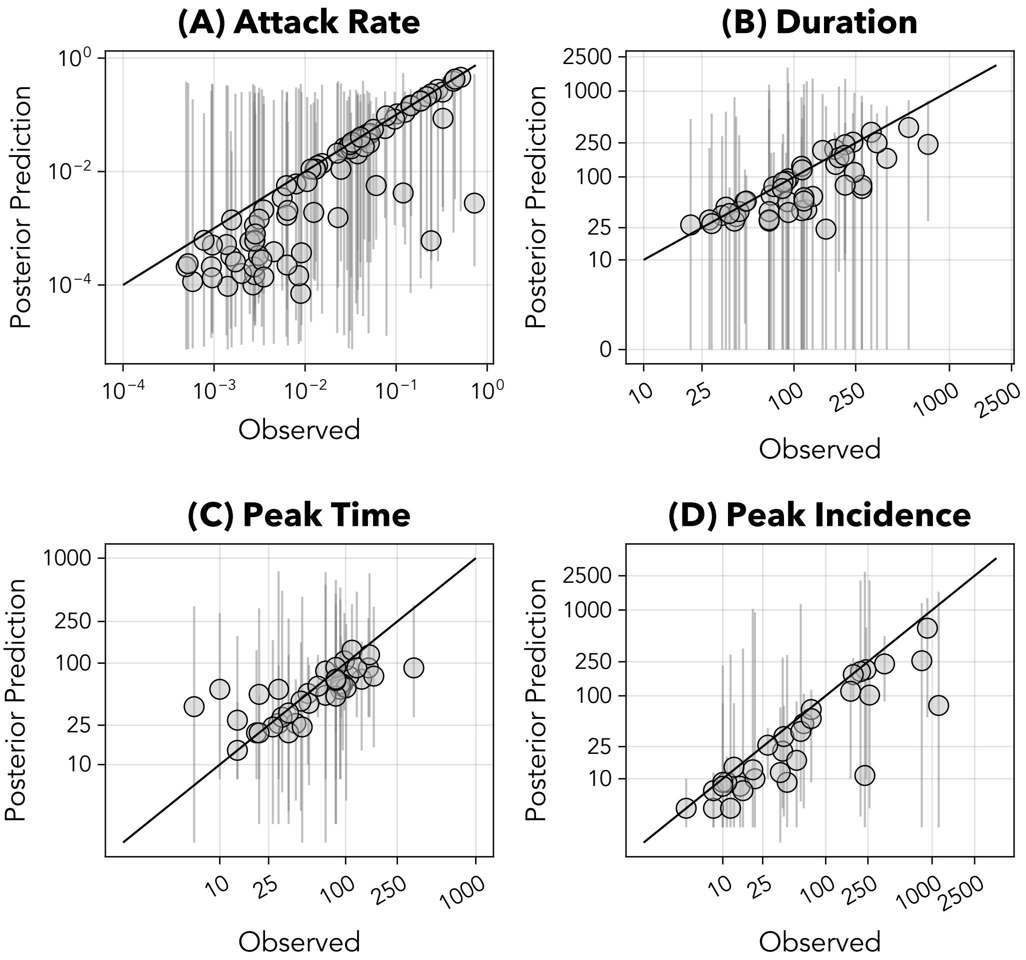

Figure S13: Posterior fits to the four most common outbreak summary statistics: (A) attack rate, (B) outbreak duration, (C) outbreak peak timing, and (D) outbreak peak incidence. Each dot represents a different outbreak, excluding outbreaks for which these data were unavailable. The horizontal position of each dot denotes the summary statistic's true value; the vertical position of the dot denotes the posterior median prediction of that statistic; and the gray error bars denote the 95% prediction interval of that statistic. The black line is $y = x$: a dot positioned on this line indicates close agreement between the data and its posterior median prediction, and an error bar overlapping with this line indicates that the data is supported by the middle 95% of its posterior predictive distribution.

these populations before data collection began. In that case, it is unsurprising that our model, which assumes that each outbreak started with just a few infections, was unable to recreate these short, intense outbreak patterns.

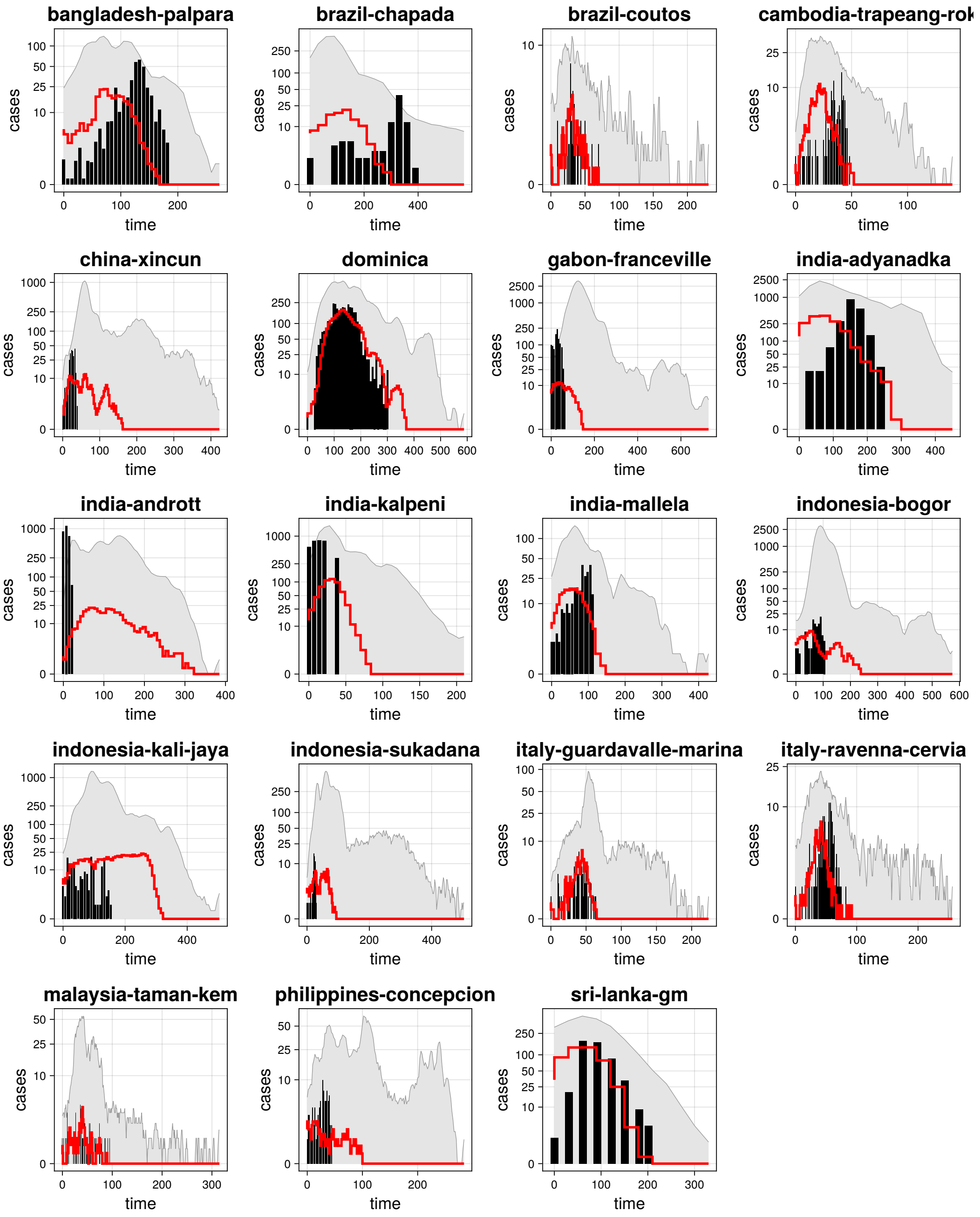

Figure S14: Available epidemic curves and posterior simulated timeseries for 19 outbreaks. Within each panel, the true epidemic curve is shown as a black barplot. The simulations are shown using simplified functional boxplots [156]: the red curve shows a median simulation, and the gray region shows where most simulated timeseries lie. Epidemic curves that protrude beyond the gray region are considered outliers relative to the simulations.

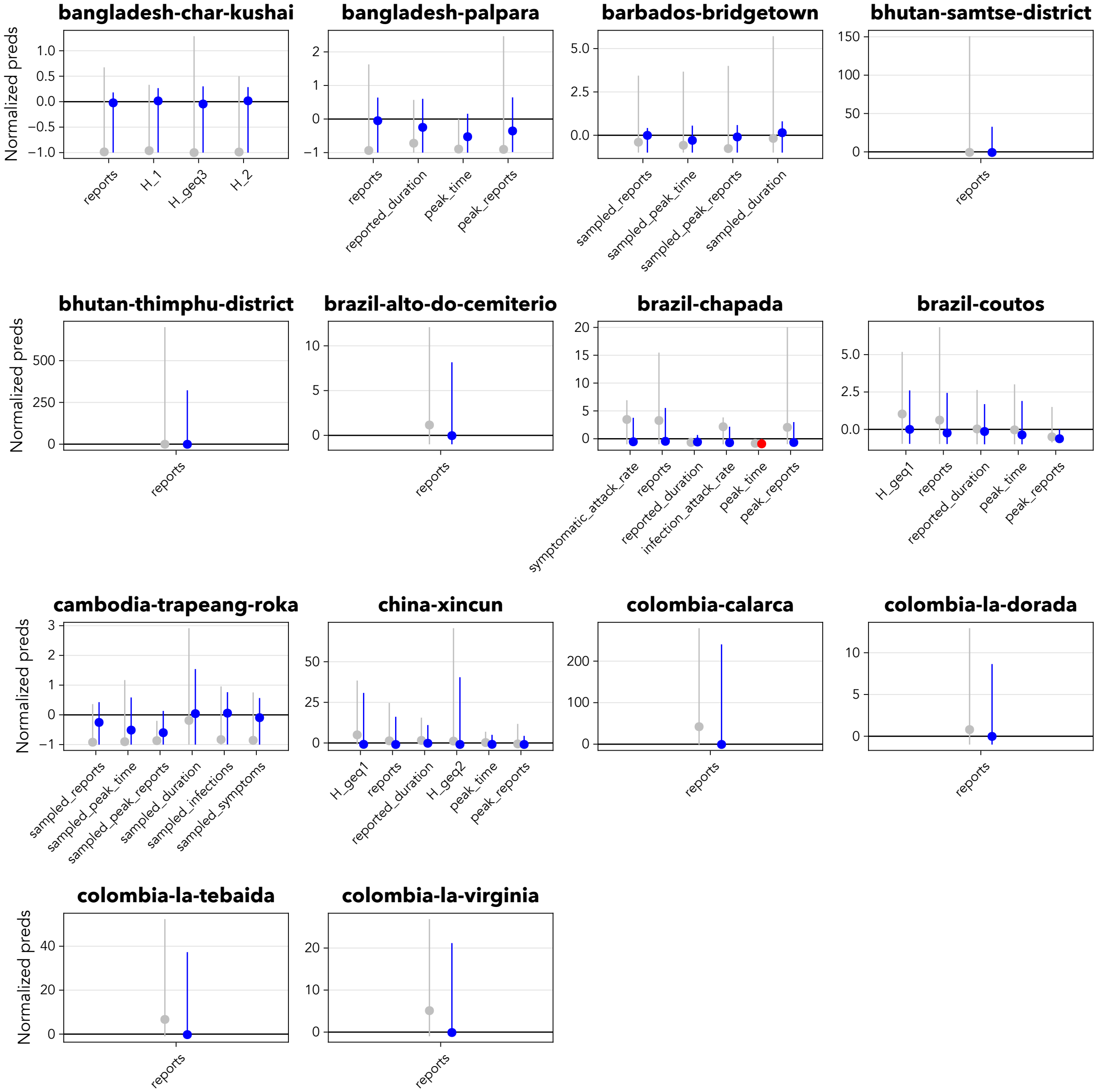

Figure S15: Prior (gray) and posterior (blue) predictive distributions of each data type for outbreaks 1-14. Dots and vertical lines show these distributions' medians and 95% prediction intervals. After normalization, a prediction of 0 (marked with a black horizontal line) indicates agreement with the data. Summary statistics whose posterior 95% prediction intervals do not contain the true value (i.e., do not cross the black horizontal line) are highlighted in red.

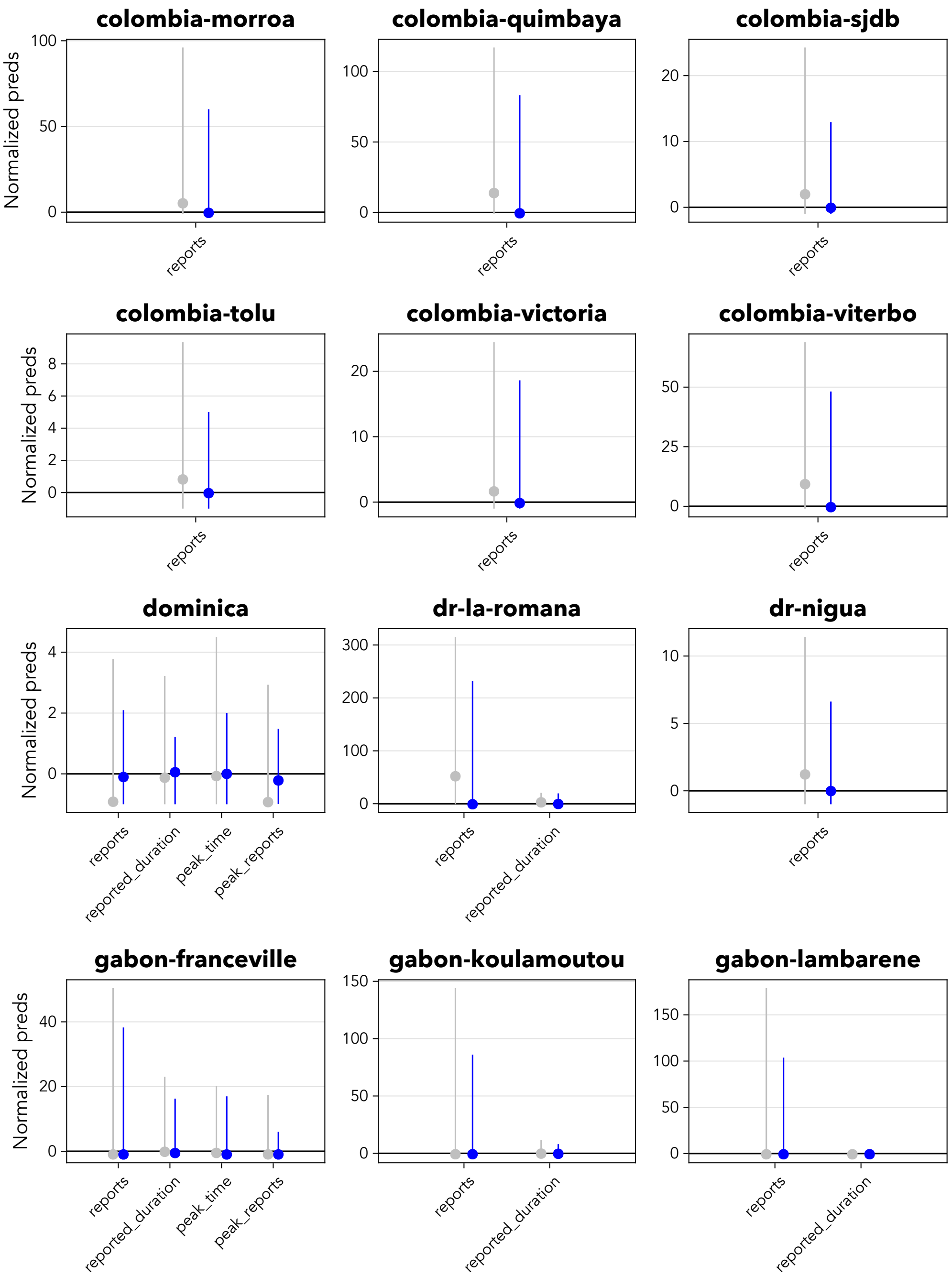

Figure S16: Prior (gray) and posterior (blue) predictive distributions of each data type for outbreaks 15-26. Dots and vertical lines show these distributions' medians and 95% prediction intervals. After normalization, a prediction of 0 (marked with a black horizontal line) indicates agreement with the data. Summary statistics whose posterior 95% prediction intervals do not contain the true value (i.e., do not cross the black horizontal line) are highlighted in red.

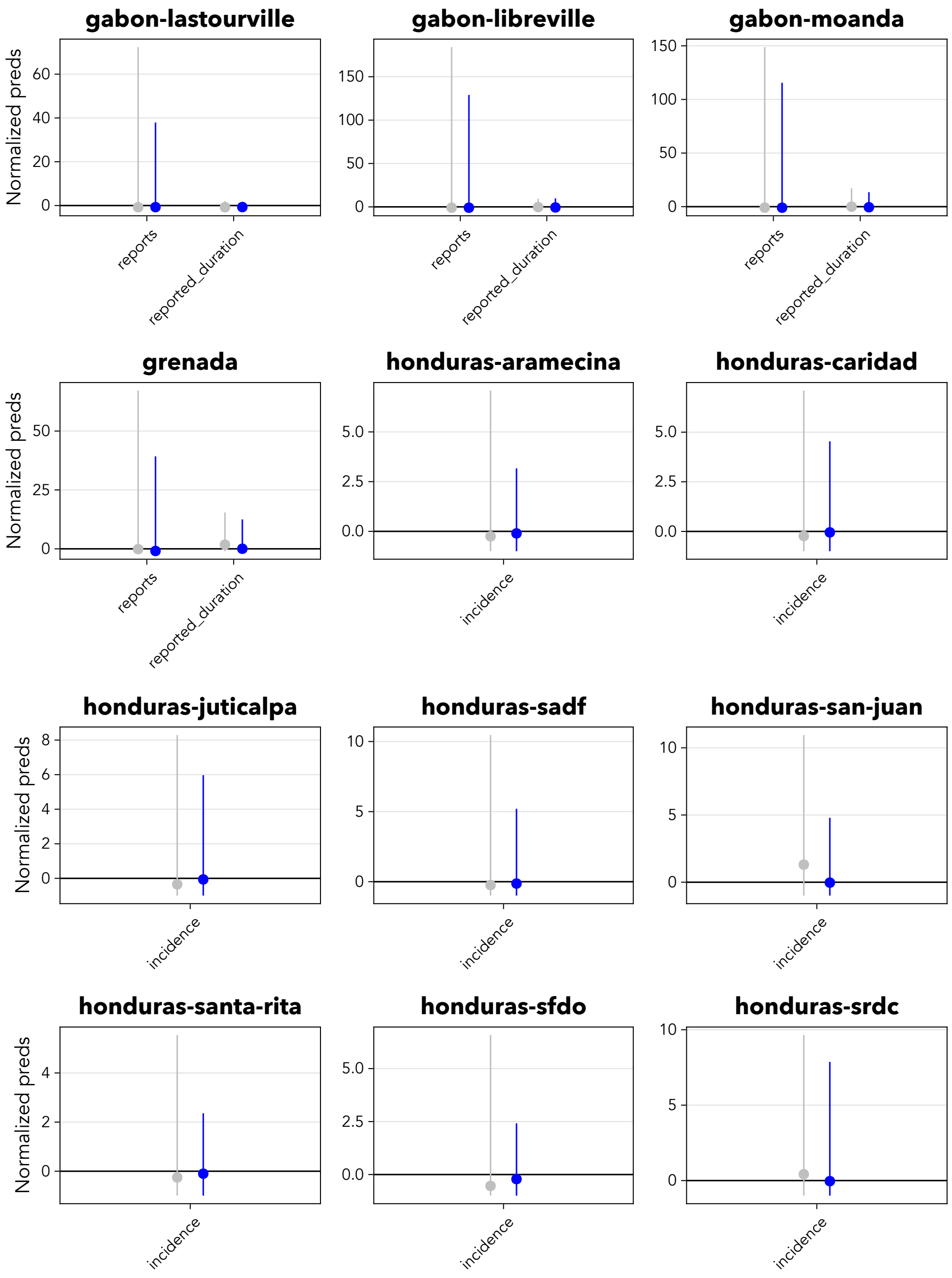

Figure S17: Prior (gray) and posterior (blue) predictive distributions of each data type for outbreaks 27-38. Dots and vertical lines show these distributions' medians and 95% prediction intervals. After normalization, a prediction of 0 (marked with a black horizontal line) indicates agreement with the data. Summary statistics whose posterior 95% prediction intervals do not contain the true value (i.e., do not cross the black horizontal line) are highlighted in red.

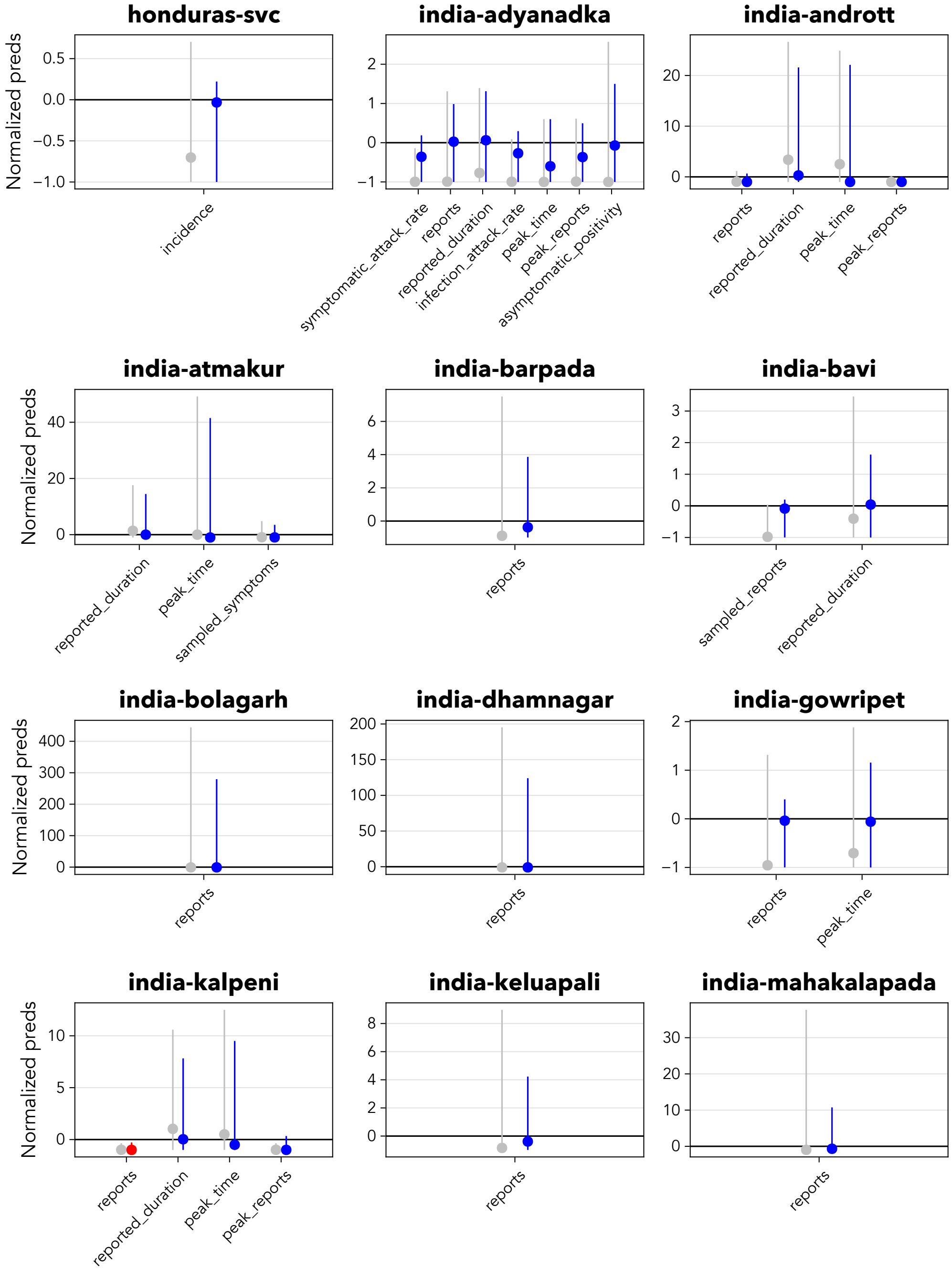

Figure S18: Prior (gray) and posterior (blue) predictive distributions of each data type for outbreaks 39-50. Dots and vertical lines show these distributions' medians and 95% prediction intervals. After normalization, a prediction of 0 (marked with a black horizontal line) indicates agreement with the data. Summary statistics whose posterior 95% prediction intervals do not contain the true value (i.e., do not cross the black horizontal line) are highlighted in red.

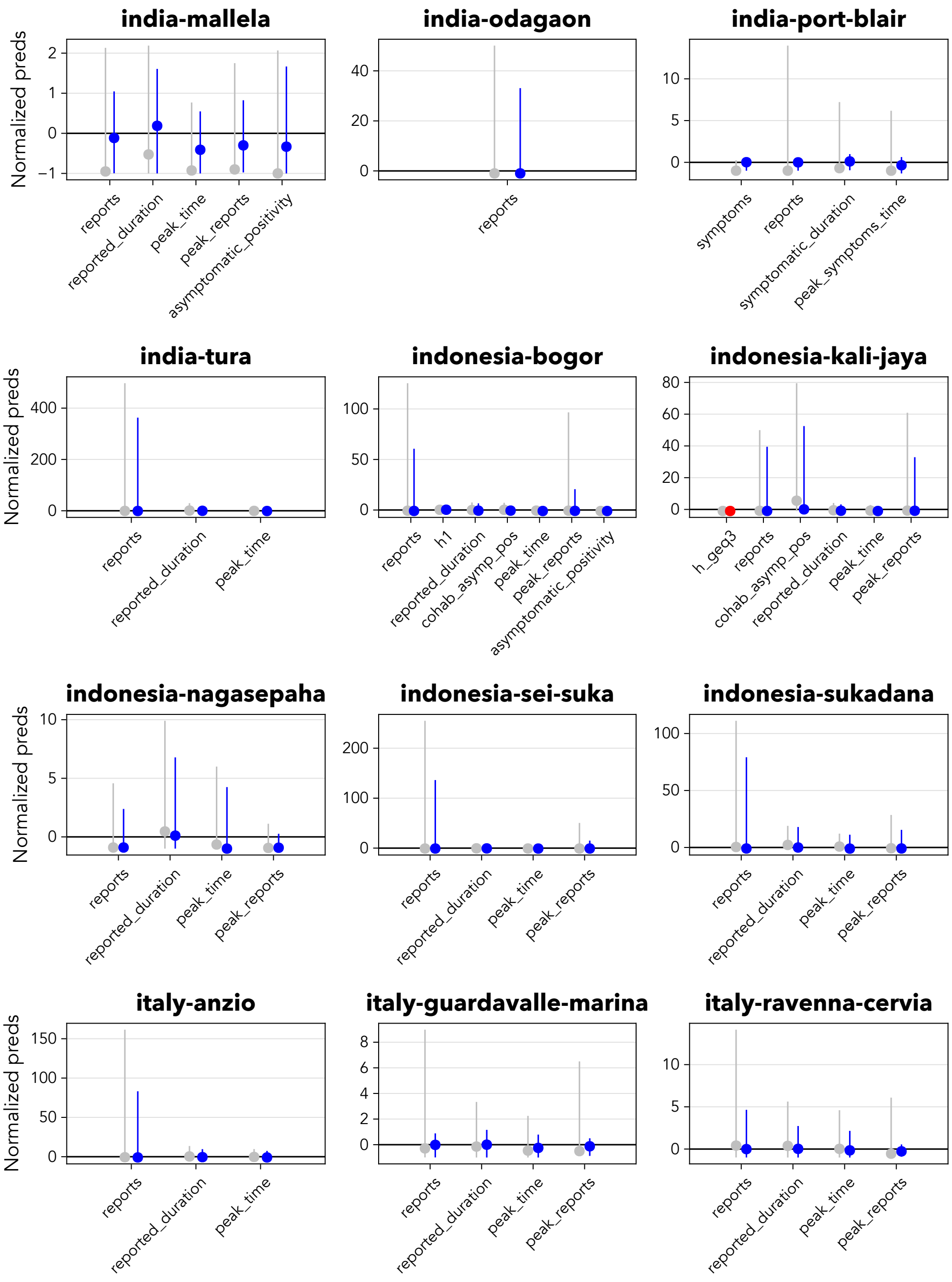

Figure S19: Prior (gray) and posterior (blue) predictive distributions of each data type for outbreaks 51-62. Dots and vertical lines show these distributions' medians and 95% prediction intervals. After normalization, a prediction of 0 (marked with a black horizontal line) indicates agreement with the data. Summary statistics whose posterior 95% prediction intervals do not contain the true value (i.e., do not cross the black horizontal line) are highlighted in red.

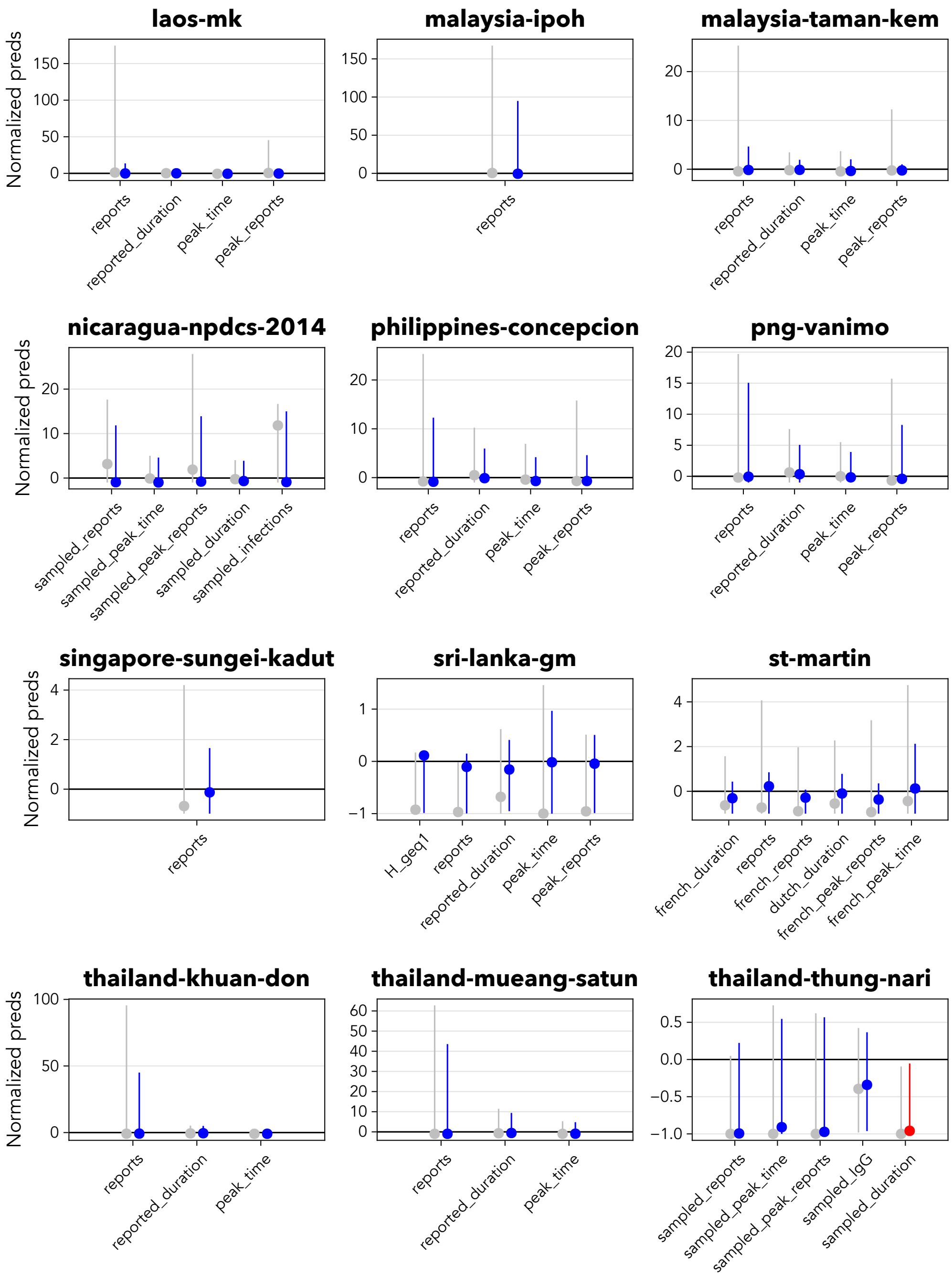

Figure S20: Prior (gray) and posterior (blue) predictive distributions of each data type for outbreaks 63-74. Dots and vertical lines show these distributions' medians and 95% prediction intervals. After normalization, a prediction of 0 (marked with a black horizontal line) indicates agreement with the data. Summary statistics whose posterior 95% prediction intervals do not contain the true value (i.e., do not cross the black horizontal line) are highlighted in red.

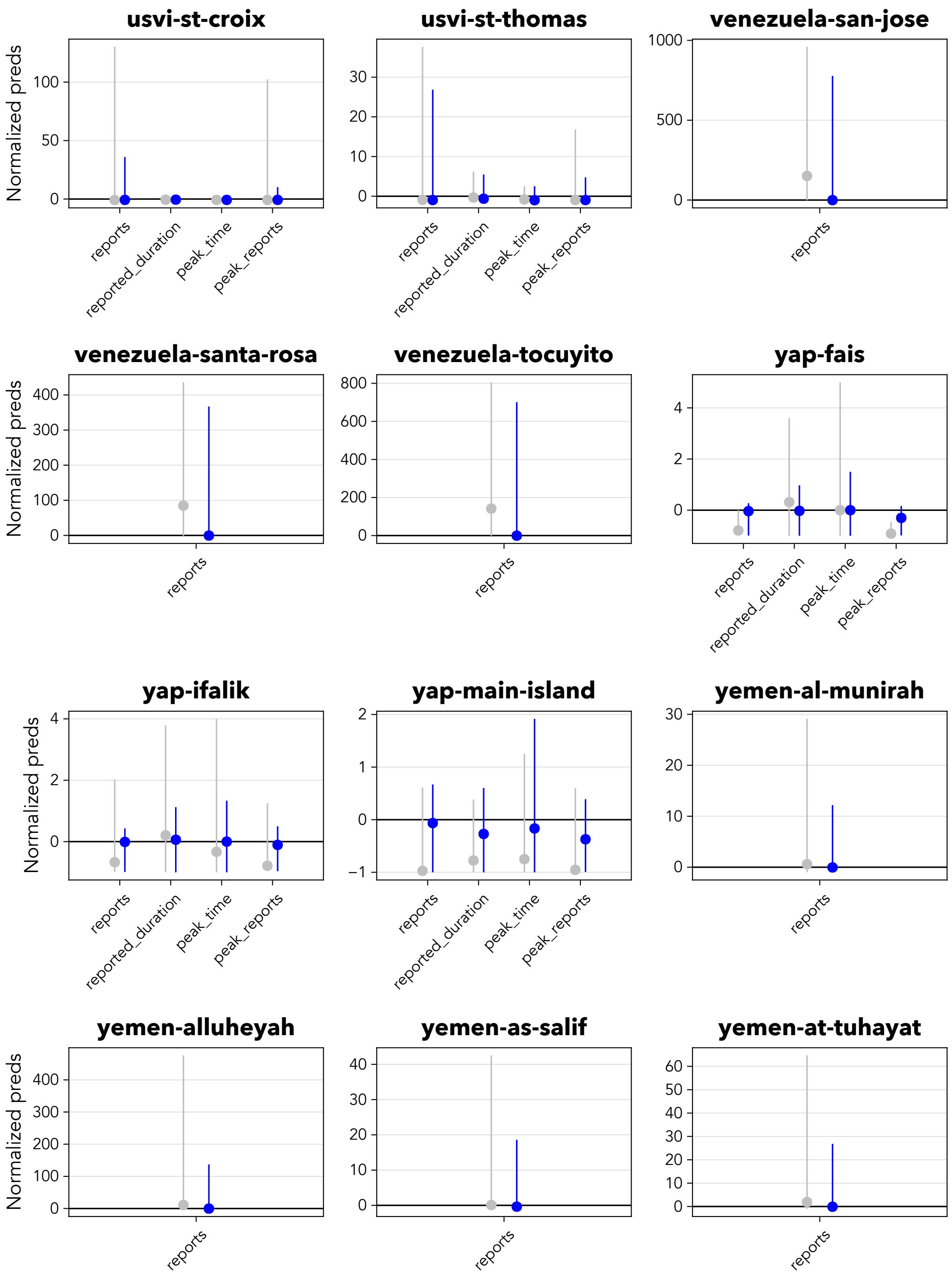

Figure S21: Prior (gray) and posterior (blue) predictive distributions of each data type for outbreaks 75-86. Dots and vertical lines show these distributions' medians and 95% prediction intervals. After normalization, a prediction of 0 (marked with a black horizontal line) indicates agreement with the data. Summary statistics whose posterior 95% prediction intervals do not contain the true value (i.e., do not cross the black horizontal line) are highlighted in red.

S4 Potential Predictors of R_0

We obtained values of the 13 non-outbreak variables in main text **Table 2** for each of the 86 outbreaks in our study.

Viral lineage & vector species. Whenever outbreak investigations reported a culpable vector species or CHIKV lineage, we used those data. For the outbreaks for which a CHIKV lineage was not reported, we inferred a lineage from the reviews by Silva et al. [113] and Rezza et al. [112]. We assumed that outbreaks of the Asian lineage of CHIKV were mediated by *Aedes aegypti* only, since no published evidence exists for *Aedes albopictus* transmitting that lineage. When vector species were not reported, we used the global occurrence probability maps for *Aedes aegypti* and *Aedes albopictus* published by Kraemer et al. [21]. These maps were available as monthly averages, and we assumed that the culpable species was the one with the greater occurrence probability in the month the outbreak began.

A key result highlighted in the main text was that our posterior estimates of R_0 differed across the three CHIKV lineages included in this study, with the highest values for the Indian Ocean Lineage (IOL), followed by the Asian lineage and the East-Central-South African (ECSA) lineage. This is visible in **Figure S22A**. Based on this result, we included viral lineage in every machine learning model we tested. We did not see a clear difference in R_0 across *Aedes* vector species.

Demographic data. Whenever an investigation reported the number of individuals and households in an affected population during an outbreak, we used those values. We used ArcGIS [95] to locate each population and determine its boundaries, then used those boundaries to extract a map of each site from WorldPop's 2020 population count maps with 100 m $\times$ 100 m grid cells [97]. We approximated the area of each site as the sum of the areas of its populated grid cells. The ArcGIS maps provided estimates of the number of individuals and household in each site, which we used when those data were not directly available from outbreak investigations. We adjusted these population size estimates from census years to outbreak years based on national-level population growth trends published by WorldBank. Finally, we used CHIKSIM's Population Submodel to simulate the locations of households in these sites based on these data. This was sufficient for us to estimate each site's population density, mean household size, and mean distance from each house to its nearest neighbor. The posterior mean estimates of R_0 appear to decrease with population density and increase with mean household size (**Figure S22B-C**. The effect of the distance between houses on R_0 is less visually clear (**Figure S22D**).

For almost all populations, we were able to find Subnational Human Development Indices (SHDIs) in the outbreak years from the dataset published by Smits et al. [64]. These data did not include SHDI estimates for the US Virgin Islands (St. Croix and St. Thomas), which we instead obtained from Cassella [116], or for the island of Saint Martin, which we obtained from Davenport [115]. Posterior mean estimates of R_0 appeared to decrease with SHDI (**Figure S22E**).

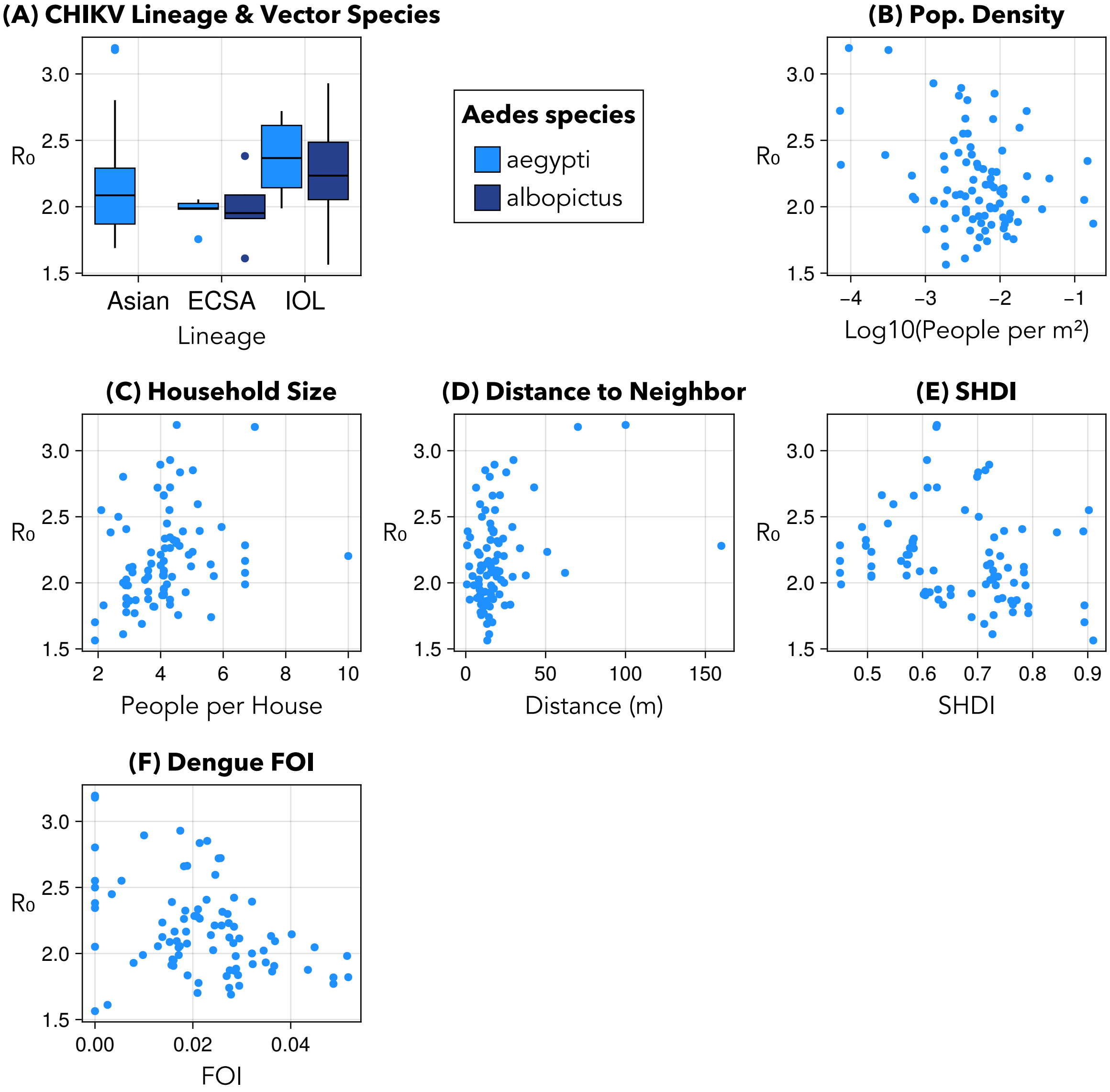

Figure S22: Exploratory data analysis of scalar predictors of R_0 In each panel, the y-axis variable is the outbreak-based posterior mean estimate of R_0.

Climatic data and other quantities. We downloaded timeseries of temperature, humidity, and precipitation at each outbreak's coordinates from one year before each outbreak until the end of 2021 from the TerraClimate database [114]. These timeseries comprised monthly averages of daily minima and maxima, which we averaged to obtain a single values for temperature, humidity, and precipitation for each month. Humidity was available as vapor pressures and saturation pressures, which we used to compute relative humidity. Visually, the associations between R_0 and these three climatic variables are not obvious (**Figure S23**, first three rows). While temperature

(annual mean and months 0 and -1) looks promising as a predictor, the most valuable of these untransfomed climatic variables turned out to be humidity in month -2 (main text **Figure 3C**).

To obtain monthly Relative R_0 estimates, we plugged the untransformed temperature estimates from TerraClimate into the *Aedes* vector-specific functions described by Mordecai et al. [29]. For the Index-P, we input the temperature and humidity estimates from TerraClimate into the R software package described by Obolski et al. [26]. Based on row 4 of **Figure S23**, a positive relationship between relative R_0 and the outbreak-based R_0 estimates seems plausible. The relationship between R_0 and Index-P is less visually clear (row 5).

Our estimates of *Aedes* occurrence probability were taken from Kraemer et al. [21], as described earlier in this section, and did not depend on the TerraClimate data. Similarly, our estimates of the force of infection for dengue at each outbreak site were taken directly from Cattarino et al. [23]. Note that while we did not include a vegetation index as a predictor, both of these variables are themselves predicted by vegetation indices. Although the *Aedes* occurrence probability turned out to be a useful predictor of R_0 (**Figure 3**), its relationship to R_0 is not clear in **Figure S23** (row 6). On the other hand, the relationship between the dengue FOI and the CHIKV R_0 appears to be negative (**Figure S22F**)—a surprising result that is discussed at length in the main text, and should not be interpreted as a causal relationship.

S5 Variance partitioning

S5.1 Five-term partition

In the main text, we described a five-term variance partition for outbreak severity (infection attack rate, outbreak duration, peak incidence of new infections, and peak timing) that used simulated outbreak data. Each simulated outbreak used parameter set P from outbreak D's posterior parameter distribution, but the population network structure from a potentially different outbreak N. The partition attributed variance in outbreak severity to parameter differences among outbreaks, parameter uncertainty, network differences among affected populations, the interaction between parameter values and network structure, and stochasticity. Let Y denote any of the four (log-transformed) aspects of outbreak severity listed above. In the main text, we used the Law of Total Variance to separate variance in Y due to stochasticity and non-stochastic factors (P, D, and N),

$$\mathsf{Var}(Y) = \underbrace{\mathsf{Var}(\mathsf{E}[Y|N,D,P])}_{\mathscr{V}_{\text{nonstoch}}} + \underbrace{\mathsf{E}[\mathsf{Var}(Y|N,D,P)]}_{\mathscr{V}_{\text{stoch}}}. \tag{S1}$$

Since $Y_{NDP} = \mathsf{E}[Y|N,D,P]$ is a random variable, we can use a similar approach to further partition its variance, $\mathscr{V}_{\text{nonstoch}}$ into

$$\mathscr{V}_{\text{nonstoch}} = \mathscr{V}_{\text{pars}} + \mathscr{V}_{\text{unc}} + \mathscr{V}_{\text{ixn}} + \mathscr{V}_{\text{network}}. \tag{S2}$$

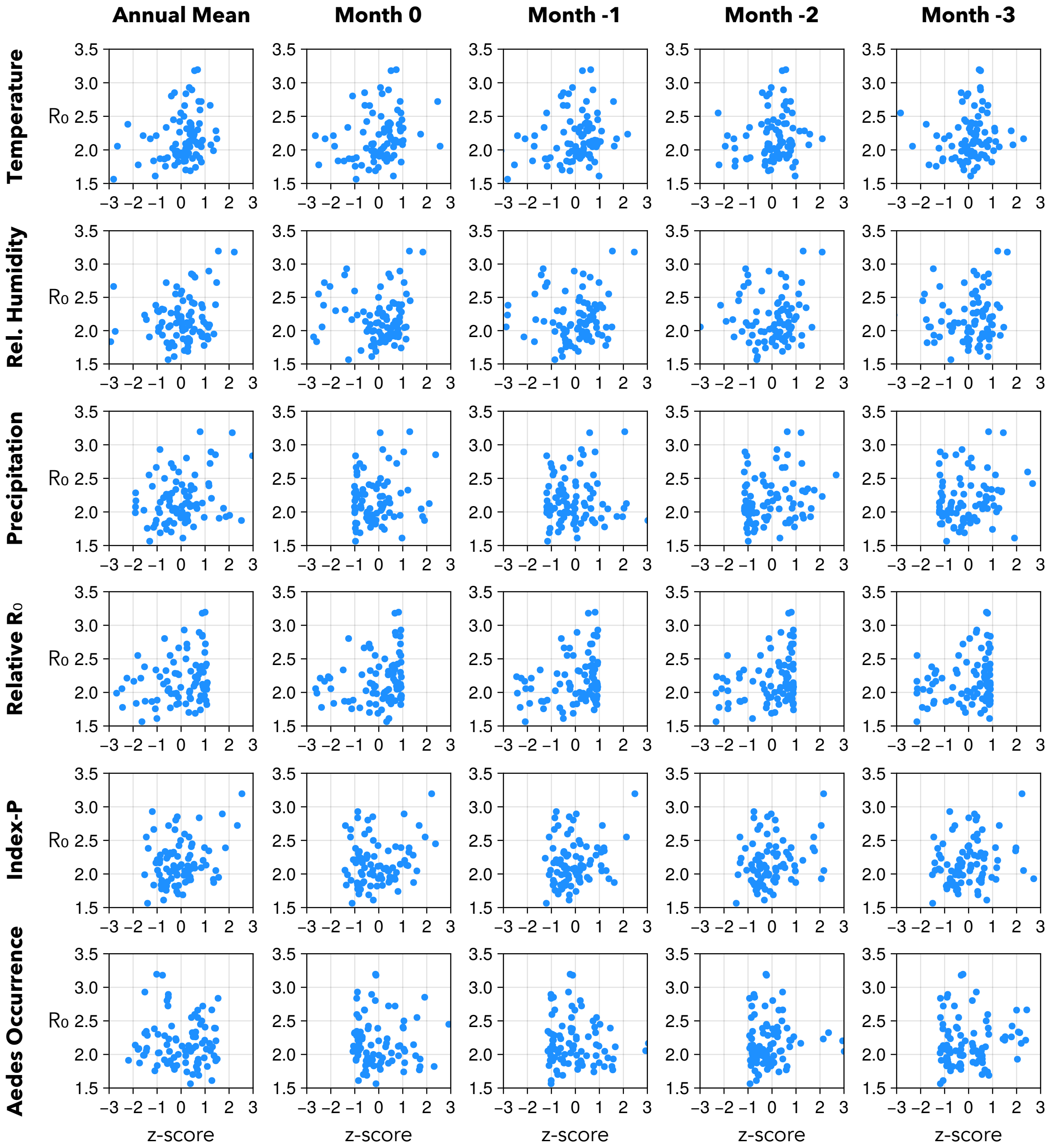

Figure S23: Exploratory data analysis of climatic predictors of R_0 available at multiple timepoints. In each panel, the y-axis variable is the outbreak-based posterior mean estimate of R_0. The x-axis variable is the climatic predictor indicated for each row at the timepoint indicated in each column. All variables were scaled to have mean 0 and unit variance to facilitate visualization.

where $\mathscr{V}_{\text{pars}}$, $\mathscr{V}_{\text{network}}$, $\mathscr{V}_{\text{ixn}}$, and $\mathscr{V}_{\text{unc}}$ represents non-stochastic due to parameter differences only, network differences only, the interaction of parameters with network structure, and parameter

uncertainty, respectively. We applied the Law of Total Variance three different ways to resolve these individual terms: with respect to network structure,

$$\mathsf{Var}(Y_{NDP}) = \underbrace{\mathsf{Var}(\mathsf{E}[Y_{NDP}|N])}_{\mathscr{V}_{\text{network}}} + \underbrace{\mathsf{E}[\mathsf{Var}(Y_{NDP}|N)]}_{\mathscr{V}_{\text{pars}}+\mathscr{V}_{\text{ixn}}+\mathscr{V}_{\text{unc}}}, \tag{S3}$$

with respect to parameter differences among populations,

$$\mathsf{Var}(Y_{NDP}) = \underbrace{\mathsf{Var}(\mathsf{E}[Y_{NDP}|D])}_{\mathscr{V}_{\text{pars}}} + \underbrace{\mathsf{E}[\mathsf{Var}(Y_{NDP}|P)]}_{\mathscr{V}_{\text{ixn}}+\mathscr{V}_{\text{unc}}+\mathscr{V}_{\text{network}}}, \tag{S4}$$

and with respect to all parameter differences,

$$\mathsf{Var}(Y_{NDP}) = \underbrace{\mathsf{Var}(\mathsf{E}[Y_{NDP}|P])}_{\mathscr{U}_3=\mathscr{V}_{\text{pars}}+\mathscr{V}_{\text{unc}}} + \underbrace{\mathsf{E}[\mathsf{Var}(Y_{NDP}|P)]}_{\mathscr{U}_4=\mathscr{V}_{\text{network}}+\mathscr{V}_{\text{ixn}}}. \tag{S5}$$

Finally, $\mathscr{V}_{\text{ixn}} = \mathscr{U}_2 - \mathscr{V}_{\text{network}}$ and $\mathscr{V}_{\text{unc}} = \mathscr{U}_1 - \mathscr{V}_{\text{pars}}$.

S5.2 Four-term partition

The five-term partition above treats parameter uncertainty as a source of variance. Another way to handle uncertainty would be to randomly select a parameter set from each outbreak's posterior distribution, P, then apply a four-term variance partition to $\hat{Y} = \mathsf{E}[Y|P]$ that attributes variance to parameters, network structure, the interaction thereof, and stochasticity. The uncertainty in $\hat{P}$ results in a posterior distribution for the variance partition, rather than an additional variance source. Formally,

$$\mathsf{Var}(\hat{Y}) = \underbrace{\mathsf{Var}(\mathsf{E}[\hat{Y}|N,D])}_{\mathscr{V}_{\text{nonstoch}}(P)} + \underbrace{\mathsf{E}[\mathsf{Var}(\hat{Y}|N,D)]}_{\mathscr{V}_{\text{stoch}}(P)}, \tag{S6}$$

then letting $\hat{Y}_{ND} = \mathsf{E}[\hat{Y}|N,D]$ and proceding as before,

$$\mathsf{Var}(\hat{Y}_{ND}) = \underbrace{\mathsf{Var}(\mathsf{E}[\hat{Y}_{ND}|N])}_{\mathscr{V}_{\text{network}}(P)} + \underbrace{\mathsf{E}[\mathsf{Var}(\hat{Y}_{ND}|N)]}_{\mathscr{U}=\mathscr{V}_{\text{pars}}(P)+\mathscr{V}_{\text{ixn}}(P)}, \tag{S7}$$

$$\mathsf{Var}(\hat{Y}_{ND}) = \underbrace{\mathsf{Var}(\mathsf{E}[\hat{Y}_{ND}|D])}_{\mathscr{V}_{\text{pars}}(P)} + \underbrace{\mathsf{E}[\mathsf{Var}(\hat{Y}_{ND}|D)]}_{\mathscr{V}_{\text{network}}(P)+\mathscr{V}_{\text{ixn}}(P)}, \tag{S8}$$

$$\mathscr{V}_{\text{ixn}}(P) = \mathscr{U} - \mathscr{V}_{\text{pars}}(P). \tag{S9}$$

This alternative partition is useful because it demonstrates that while uncertainty increases the total variance in outbreak severity Y, it does not alter the fraction of that variance attributable to parameter values ($\mathscr{V}_{\text{pars}} + \mathscr{V}_{\text{unc}}$ in the five-term partition, and the mean of $\mathscr{V}_{\text{pars}}(P)$ in the four-term partition). This is visible in Figure S24, which compares the five-term variance partition from the main text to four-term one presented here.

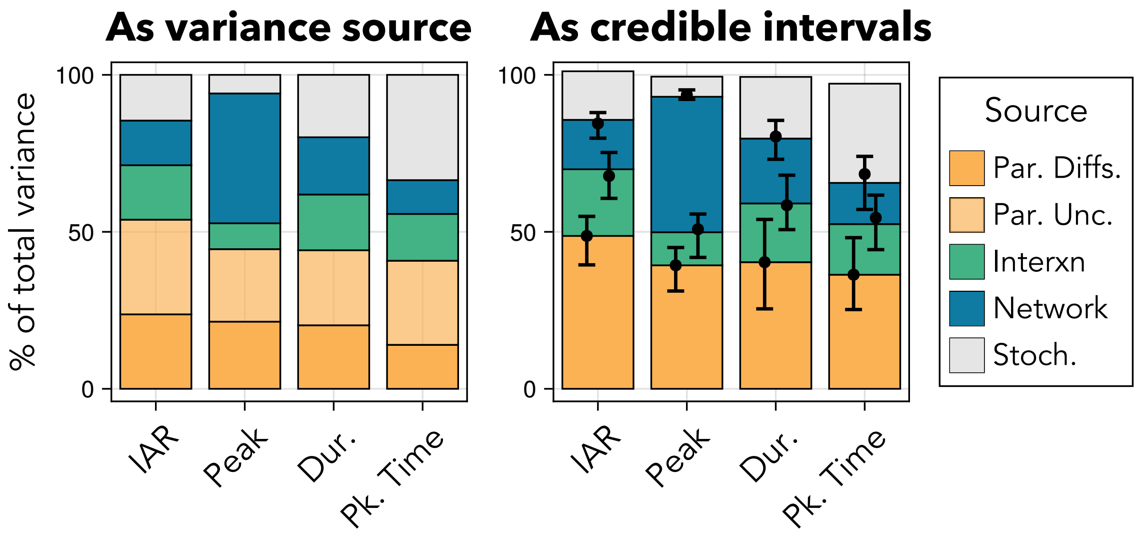

Figure S24: Variance partitions for log-transformed infection attack rate, peak daily incidence of new CHIKV infections, outbreak duration, and peak timing. The five-term partition on the left treats parameter uncertainty as a source of variance in outbreak severity. This is the same as main text **Figure 4A**. The four-term partition on the right instead translates parameter uncertainty into uncertainty in the partition. The error bars indicate posterior 95% credible intervals for the divisions in the variance partition (in increasing order: $\mathscr{V}_{\text{pars}}(P)$, $\mathscr{V}_{\text{nonstoch}}(P) - \mathscr{V}_{\text{pars}}(P)$, and $\mathscr{V}_{\text{nonstoch}}(P)$).

S5.3 Variance in R_0 and R_e due to network structure

We used the five-term variance partition to quantify the contribution of network structure to variability in our R_0 and $R_e = (1 - p_{\text{immune}})R_0$. Because these quantities are deterministic, $\mathscr{V}_{\text{stoch}} = 0$ for both. Surprisingly, the entirety of the non-stochastic variance was attributable to parameter values ($\mathscr{V}_{\text{network}} = \mathscr{V}_{\text{ixn}} = 0$ for both R_0 and R_e), with differences among populations contributing 30% of the variance in R_0 and 32% of the variance in R_e, and the remaining fractions due to parameter uncertainty. This means that R_0 was almost completely determined by parameter values, rather than a combination of parameters and population network structure.

S6 Supplemental Data

Data S1: Non-outbreak predictors of R_0

This data set contains the non-outbreak data from each outbreak that was used during our regression analysis of R_0, along with other relevant information. This includes population size, latitude and longitude coordinates, outbreak features (e.g., attack rate and duration, if known), outbreak start and end month (if known; -1 if not), vector species and viral lineage, and climatic variables (annual means and values $n = 0, \ldots, 11$ months prior to each outbreak, e.g., `temp_lag1` for temperature one month prior). The column `ref_number` provides the citation number for each outbreak within this study's bibliography.

Data S2: Posterior parameter estimates

This data set contains 1,000 posterior samples of R_0, R_e, and all model parameters (B, C, p_{immune}, p_{asymp}, p_{report}, p_{house}, L, G, I_0) for each of the 86 chikungunya outbreaks.

REFERENCES AND NOTES

1. M. L. Childs, N. Nova, J. Colvin, E. A. Mordecai, Mosquito and primate ecology predict human risk of yellow fever virus spillover in Brazil. *Philos. Trans. R. Soc. B. Biol. Sci.* **374**, 20180335 (2019).

2. K. E. Jones, N. G. Patel, M. A. Levy, A. Storeygard, D. Balk, J. L. Gittleman, P. Daszak, Global trends in emerging infectious diseases. *Nature* **451**, 990–993 (2008).

3. A. J. Tatem, D. J. Rogers, S. I. Hay, "Advances in parasitology," in *Global Mapping of Infectious Diseases: Methods*, *Examples and Emerging Applications*, S. I. Hay, A. Graham, D. J. Rogers, Eds. (Academic Press, 2006), vol. 62, pp. 293–343.

4. M. E. J. Woolhouse, D. T. Haydon, R. Antia, Emerging pathogens: The epidemiology and evolution of species jumps. *Trends Ecol. Evol.* **20**, 238–244 (2005).

5. J. C. Miller, Distribution of outbreak sizes for SIR disease in finite populations. arXiv:1907.05138 (2019).

6. F. Brauer, C. Castillo-Chavez, *Mathematical Models in Population Biology and Epidemiology*, vol. 40 of *Texts in Applied Mathematics* (Springer, 2012).

7. M. J. Keeling, P. Rohani, *Modeling Infectious Diseases in Humans and Animals* (Princeton Univ. Press, 2008).

8. H. W. Hethcote, The mathematics of infectious diseases. *SIAM Rev.* **42**, 599–653 (2000).

9. A. D. Barbour, The duration of the closed stochastic epidemic. *Biometrika* **62**, 477–482 (1975).

10. B. P. Bell, Overview, control strategies, and lessons learned in the CDC response to the 2014–2016 Ebola epidemic. *MMWR Suppl.* **65**, 4–11 (2016).

11. D. Griffith, L. Kelly-Hope, M. Miller, Review of reported cholera outbreaks worldwide, 1995–2005. *Am. J. Trop. Med. Hyg.* **75**, 973–977 (2006).

12. A. Endo, S. Abbott, A. J. Kucharski, S. Funk, Estimating the overdispersion in COVID-19 transmission using outbreak sizes outside China. *Wellcome Open Res.* **5**, 67 (2020).

13. Y. Liu, K. Lillepold, J. C. Semenza, Y. Tozan, M. B. M. Quam, J. Rocklöv, Reviewing estimates of the basic reproduction number for dengue, Zika and chikungunya across global climate zones. *Environ. Res.* **182**, 109114 (2020).

14. L. P. Lounibos, L. D. Kramer, Invasiveness of *Aedes aegypti* and *Aedes albopictus* and vectorial capacity for chikungunya virus. *J. Infect. Dis.* **214**, S453–S458 (2016).

15. H. Padmanabha, F. Correa, C. Rubio, A. Baeza, S. Osorio, J. Mendez, J. H. Jones, M. A. Diuk-Wasser, Human social behavior and demography drive patterns of fine-scale dengue transmission in endemic areas of Colombia. *PLOS ONE* **10**, e0144451 (2015).

16. A. Camacho, A. J. Kucharski, S. Funk, J. Breman, P. Piot, W. J. Edmunds, Potential for large outbreaks of Ebola virus disease. *Epidemics* **9**, 70–78 (2014).

17. E. A. Mordecai, J. M. Caldwell, M. K. Grossman, C. A. Lippi, L. R. Johnson, M. Neira, J. R. Rohr, S. J. Ryan, V. Savage, M. S. Shocket, R. Sippy, A. M. Stewart Ibarra, M. B. Thomas, O. Villena, Thermal biology of mosquito-borne disease. *Ecol. Lett.* **22**, 1690–1708 (2019).

18. B. Ridenhour, J. M. Kowalik, D. K. Shay, Unraveling R_0: Considerations for public health applications. *Am. J. Public Health* **104**, e32 (2014).

19. D. L. Smith, F. E. McKenzie, R. W. Snow, S. I. Hay, Revisiting the basic reproductive number for malaria and its implications for malaria control. *PLOS Biol.* **5**, e42 (2007).

20. J. Heffernan, R. Smith, L. Wahl, Perspectives on the basic reproductive ratio. *J. R. Soc. Int.* **2**, 281–293 (2005).

21. M. U. Kraemer, M. E. Sinka, K. A. Duda, A. Q. N. Mylne, F. M. Shearer, C. M. Barker, C. G. Moore, R. G. Carvalho, G. E. Coelho, W. Van Bortel, G. Hendrickx, F. Schaffner, I. R. F. Elyazar, H.-J. Teng, O. J. Brady, J. P. Messina, D. M. Pigott, T. W. Scott, D. L. Smith, G. R. W. Wint, N. Golding, S. I. Hay, The global distribution of the arbovirus vectors *Aedes aegypti* and *Ae. albopictus*. *eLife* **4**, e08347 (2015).

22. T. Nakase, M. Giovanetti, U. Obolski, J. Lourenço, Global transmission suitability maps for dengue virus transmitted by *Aedes aegypti* from 1981 to 2019. *Sci. Data* **10**, 275 (2023).

23. L. Cattarino, I. Rodriguez-Barraquer, N. Imai, D. A. T. Cummings, N. M. Ferguson, Mapping global variation in dengue transmission intensity. *Sci. Trans. Med.* **12**, eaax4144 (2020).

24. G. Muñoz, X. Chourio, A. Rivière-Cinnamond, M. A. Diuk-Wasser, P. A. Kache, E. A. Mordecai, L. Harrington, M. C. Thomson, *Ae*DES: A next-generation monitoring and forecasting system for environmental suitability of *Aedes*-borne disease transmission. *Sci. Rep.* **10**, 12640 (2020).

25. M. U. G. Kraemer, R. C. Reiner Jr., O. J. Brady, J. P. Messina, M. Gilbert, D. M. Pigott, D. Yi, K. Johnson, L. Earl, L. B. Marczak, S. Shirude, N. D. Weaver, D. Bisanzio, T. Alex Perkins, S. Lai, X. Lu, P. Jones, G. E. Coelho, R. G. Carvalho, W. Van Bortel, C. Marsboom, G. Hendrickx, F. Schaffner, C. G. Moore, H. H. Nax, L. Bengtsson, E. Wetter, A. J. Tatem, J. S. Brownstein, D. L. Smith, L. Lambrechts, S. Cauchemez, C. Linard, N. R. Faria, O. G. Pybus, T. W. Scott, Q. Liu, H. Yu, G. R. William Wint, S. I. Hay, N. Golding, Past and future spread of the arbovirus vectors *Aedes aegypti* and *Aedes albopictus*. *Nat. Microbiol.* **4**, 854–863 (2019).

26. U. Obolski, P. N. Perez, C. J. Villabona-Arenas, J. Thézé, N. R. Faria, J. Lourenço, MVSE: An R-package that estimates a climate-driven mosquito-borne viral suitability index. *Methods Ecol. Evol.* **10**, 1357–1370 (2019).

27. S. J. Ryan, C. J. Carlson, E. A. Mordecai, L. R. Johnson, Global expansion and redistribution of *Aedes*-borne virus transmission risk with climate change. *PLOS Negl. Trop. Dis.* **13**, e0007213 (2019).

28. B. Tesla, L. R. Demakovsky, E. A. Mordecai, S. J. Ryan, M. H. Bonds, C. N. Ngonghala, M. A. Brindley, C. C. Murdock, Temperature drives Zika virus transmission: Evidence from empirical and mathematical models. *Proc. R. Soc. B. Biol. Sci.* **285**, 20180795 (2018).

29. E. A. Mordecai, J. M. Cohen, M. V. Evans, P. Gudapati, L. R. Johnson, C. A. Lippi, K. Miazgowicz, C. C. Murdock, J. R. Rohr, S. J. Ryan, V. Savage, M. S. Shocket, A. S. Ibarra,

M. B. Thomas, D. P. Weikel, Detecting the impact of temperature on transmission of Zika, dengue, and chikungunya using mechanistic models. *PLOS Negl. Trop. Dis.* **11**, e0005568 (2017).

30. O. J. Brady, N. Golding, D. M. Pigott, M. U. G. Kraemer, J. P. Messina, R. C. Reiner Jr., T. W. Scott, D. L. Smith, P. W. Gething, S. I. Hay, Global temperature constraints on *Aedes aegypti* and *Ae. albopictus* persistence and competence for dengue virus transmission. *Parasit. Vectors* **7**, 338 (2014).

31. J. Liu-Helmersson, H. Stenlund, A. Wilder-Smith, J. Rocklöv, Vectorial capacity of *Aedes aegypti*: Effects of temperature and implications for global dengue epidemic potential. *PLOS ONE* **9**, e89783 (2014).

32. H. Salje, J. Lessler, K. K. Paul, A. S. Azman, M. Waliur Rahman, M. Rahman, D. Cummings, E. S. Gurley, S. Cauchemez, How social structures, space, and behaviors shape the spread of infectious diseases using chikungunya as a case study. *Proc. Natl. Acad. Sci. U.S.A.* **113**, 13420–13425 (2016).

33. R. C. Reiner, S. T. Stoddard, T. W. Scott, Socially structured human movement shapes dengue transmission despite the diffusive effect of mosquito dispersal. *Epidemics* **6**, 30–36 (2014).

34. T. A. Perkins, T. W. Scott, A. L. Menach, D. L. Smith, Heterogeneity, mixing, and the spatial scales of mosquito-borne pathogen transmission. *PLOS Comput. Biol.* **9**, e1003327 (2013).

35. J. C. Miller, Epidemic size and probability in populations with heterogeneous infectivity and susceptibility. *Phys. Rev. E* **76**, 010101 (2007).

36. J. O. Lloyd-Smith, S. J. Schreiber, P. E. Kopp, W. M. Getz, Superspreading and the effect of individual variation on disease emergence. *Nature* **438**, 355–359 (2005).

37. L. A. Meyers, B. Pourbohloul, M. Newman, D. M. Skowronski, R. C. Brunham, Network theory and SARS: Predicting outbreak diversity. *J. Theor. Biol.* **232**, 71–81 (2005).

38. A. D. Meyer, S. M. Guerrero, N. E. Dean, K. B. Anderson, S. T. Stoddard, T. A. Perkins, Model-based estimates of chikungunya epidemiological parameters and outbreak risk from varied data types. *Epidemics* **45**, 100721 (2023).

39. L. B. Tauro, C. W. Cardoso, R. L. Souza, L. C. J. Nascimento, D. R. Santos, G. S. Campos, S. Sardi, O. B. Reis, M. G. Reis, U. Kitron, G. S. Ribeiro, A localized outbreak of Chikungunya virus in Salvador, Bahia, Brazil. *Mem. Inst. Oswaldo Cruz* **114**, e180597 (2019).

40. S. P. Manimunda, D. Mavalankar, T. Bandyopadhyay, A. P. Sugunan, Chikungunya epidemic-related mortality. *Epidemiol. Infect.* **139**, 1410–1412 (2011).

41. E. Lizarazo, M. Vincenti-Gonzalez, M. E. Grillet, S. Bethencourt, O. Diaz, N. Ojeda, H. Ochoa, M. A. Rangel, A. Tami, Spatial dynamics of Chikungunya virus, Venezuela, 2014. *Emerg. Infect. Dis.* **25**, 672–680 (2019).

42. P. P. Samuel, R. Krishnamoorthi, K. K. Hamzakoya, C. S. Aggarwal, Entomo-epidemiological investigations on chikungunya outbreak in the Lakshadweep islands, Indian Ocean. *Indian J. Med. Res.* **129**, 442–445 (2009).

43. P. Nakkhara, V. Chongsuvivatwong, S. Thammapalo, Risk factors for symptomatic and asymptomatic chikungunya infection. *Trans. R. Soc. Trop. Med. Hyg.* **107**, 789–796 (2013).

44. R. V. Cunha, K. S. Trinta, C. A. Montalbano, M. V. F. Sucupira, M. M. de Lima, E. Marques, I. H. Romanholi, J. Croda, Seroprevalence of chikungunya virus in a rural community in Brazil. *PLOS Negl. Trop. Dis.* **11**, e0005319 (2017).

45. F. Vairo, A. Mammone, S. Lanini, E. Nicastri, C. Castilletti, F. Carletti, V. Puro, D. di Lallo, V. Panella, D. Varrenti, P. Scaramozzino, A. di Caro, P. Scognamiglio, M. R. Capobianchi, G. Ippolito, Chikungunya Lazio Outbreak Group, Local transmission of chikungunya in Rome and the Lazio region, Italy. *PLoS ONE* **13**, e0208896 (2018).

46. D. M. Pastula, W. T. Hancock, M. Bel, H. Biggs, M. Marfel, R. Lanciotti, J. Laven, T. H. Chen, J. E. Staples, M. Fischer, S. L. Hills, Chikungunya virus disease outbreak in Yap State, Federated States of Micronesia. *PLOS Negl. Trop. Dis.* **11**, e0005410 (2017).

47. A. Gordon, L. Gresh, S. Ojeda, G. Chowell, K. Gonzalez, N. Sanchez, S. Saborio, J. C. Mercado, G. Kuan, A. Balmaseda, E. Harris, Differences in transmission and disease severity between 2 successive waves of chikungunya. *Clin. Infect. Dis.* **67**, 1760–1767 (2018).

48. A. A. Bettis, M. L'Azou Jackson, I.-K. Yoon, J. Gabrielle Breugelmans, A. Goios, D. J. Gubler, A. M. Powers, The global epidemiology of chikungunya from 1999 to 2020: A systematic literature review to inform the development and introduction of vaccines. *PLOS Negl. Trop. Dis.* **16**, e0010069 (2022).

49. H. Noël, C. Rizzo, Spread of chikungunya from the Caribbean to mainland Central and South America: A greater risk of spillover in Europe? *Eurosurveillance* **19**, 20855 (2014).

50. G. Rezza, L. Nicoletti, R. Angelini, R. Romi, A. C. Finarelli, M. Panning, P. Cordioli, C. Fortuna, S. Boros, F. Magurano, G. Silvi, P. Angelini, M. Dottori, M. G. Ciufolini, G. C. Majori, A. Cassone, Infection with chikungunya virus in Italy: An outbreak in a temperate region. *Lancet* **370**, 1840–1846 (2007).

51. Z. Qiaoli, H. Jianfeng, W. De, W. Zijun, Z. Xinguang, Z. Haojie, D. Fan, L. Zhiquan, W. Shiwen, H. Zhenyu, Z. Yonghui, K. Changwen, Y. Dakang, L. Wenjia, L. Deqiong, C. Pinghua, Maiden outbreak of chikungunya in Dongguan City, Guangdong Province, China: Epidemiological characteristics. *PLOS ONE* **7**, e42830 (2012).

52. C. Soulaphy, P. Souliphone, K. Phanthavong, D. Phonekeo, S. Phimmasine, B. Khamphaphongphane, V. Kitthiphong, H. C. Lewis, Emergence of chikungunya in Moonlapamok and Khong Districts, Champassak Province, the Lao People's Democratic Republic, May to September 2012. *Western Pac. Surveill. Response J* **4**, 46–50 (2013).

53. P. F. Horwood, L. J. Reimer, R. Dagina, M. Susapu, G. Bande, M. Katusele, G. Koimbu, S. Jimmy, B. Ropa, P. M. Siba, B. I. Pavlin, Outbreak of chikungunya virus infection, Vanimo, Papua New Guinea. *Emerg. Infect. Dis.* **19**, 1535–1538 (2013).

54. M. R. Malik, A. Mnzava, E. Mohareb, A. Zayed, A. al Kohlani, A. A. K. Thabet, H. el Bushra, Chikungunya outbreak in Al-Hudaydah, Yemen, 2011: Epidemiological

characterization and key lessons learned for early detection and control. *J. Epidemiol. Glob. Health* **4**, 203–211 (2014).

55. K. Laras, N. C. Sukri, R. P. Larasati, M. J. Bangs, R. Kosim, Djauzi, T. Wandra, J. Master, H. Kosasih, S. Hartati, C. Beckett, E. R. Sedyaningsih, H. J. Beecham III, A. L. Corwin, Tracking the re-emergence of epidemic chikungunya virus in Indonesia. *Trans. R. Soc. Trop. Med. Hyg.* **99**, 128–141 (2005).

56. F. Bustos Carrillo, D. Collado, N. Sanchez, S. Ojeda, B. L. Mercado, R. Burger-Calderon, L. Gresh, A. Gordon, A. Balmaseda, G. Kuan, E. Harris, Epidemiological evidence for lineage-specific differences in the risk of inapparent chikungunya virus infection. *J. Virol.* **93**, e01622-18 (2019).

57. J. Riou, C. Poletto, P.-Y. Boëlle, A comparative analysis of Chikungunya and Zika transmission. *Epidemics* **19**, 43–52 (2017).

58. O. J. Brady, M. A. Johansson, C. A. Guerra, S. Bhatt, N. Golding, D. M. Pigott, H. Delatte, M. G. Grech, P. T. Leisnham, R. Maciel-de-Freitas, L. M. Styer, D. L. Smith, T. W. Scott, P. W. Gething, S. I. Hay, Modelling adult *Aedes aegypti* and *Aedes albopictus* survival at different temperatures in laboratory and field settings. *Parasit. Vectors* **6**, 351 (2013).

59. C. T. Codeço, D. A. M. Villela, F. C. Coelho, Estimating the effective reproduction number of dengue considering temperature-dependent generation intervals. *Epidemics* **25**, 101–111 (2018).

60. A. S. Siraj, R. J. Oidtman, J. H. Huber, M. U. G. Kraemer, O. J. Brady, M. A. Johansson, T. A. Perkins, Temperature modulates dengue virus epidemic growth rates through its effects on reproduction numbers and generation intervals. *PLOS Negl. Trop. Dis.* **11**, e0005797 (2017).

61. T. A. Perkins, C. J. E. Metcalf, B. T. Grenfell, A. J. Tatem, Estimating drivers of autochthonous transmission of chikungunya virus in its invasion of the Americas. *PLOS Curr.* **7**, 10.1371/currents.outbreaks.a4c7b6ac10e0420b1788c9767946d1fc (2015).

62. S. P. Manimunda, A. P. Sugunan, S. K. Rai, P. Vijayachari, A. N. Shriram, S. Sharma, N. Muruganandam, I. K. Chaitanya, D. R. Guruprasad, A. B. Sudeep, Outbreak of chikungunya fever, Dakshina Kannada District, South India, 2008. *Am. J. Trop. Med. Hyg.* **83**, 751–754 (2010).

63. E. Štrumbelj, I. Kononenko, Explaining prediction models and individual predictions with feature contributions. *Knowl. Inf. Syst.* **41**, 647–665 (2014).

64. J. Smits, I. Permanyer, The subnational human development database. *Sci. Data* **6**, 190038 (2019).

65. L. Hébert-Dufresne, B. M. Althouse, S. V. Scarpino, A. Allard, Beyond R_0: Heterogeneity in secondary infections and probabilistic epidemic forecasting. *J. R. Soc. Int.* **17**, 20200393 (2020).

66. E. Plischke, An effective algorithm for computing global sensitivity indices (EASI). *Reliab. Eng. Syst. Safe.* **95**, 354–360 (2010).

67. C. Fritzell, D. Rousset, A. Adde, M. Kazanji, M. D. Van Kerkhove, C. Flamand, Current challenges and implications for dengue, chikungunya and Zika seroprevalence studies worldwide: A scoping review. *PLOS Negl. Trop. Dis.* **12**, e0006533 (2018).

68. Q. M. Tran, J. Soda, A. Siraj, S. Moore, H. Clapham, T. Alex Perkins, Expected endpoints from future chikungunya vaccine trial sites informed by serological data and modeling. *Vaccine* **41**, 182–192 (2023).

69. J. Rocklöv, Y. Tozan, A. Ramadona, M. O. Sewe, B. Sudre, J. Garrido, C. B. de Saint Lary, W. Lohr, J. C. Semenza, Using big data to monitor the introduction and spread of Chikungunya, Europe, 2017. *Emerg. Infect. Dis.* **25**, 1041–1049 (2019).

70. X. Feng, X. Huo, B. Tang, S. Tang, K. Wang, J. Wu, Modelling and analyzing virus mutation dynamics of chikungunya outbreaks. *Sci. Rep.* **9**, 2860 (2019).

71. P. Poletti, G. Messeri, M. Ajelli, R. Vallorani, C. Rizzo, S. Merler, Transmission potential of chikungunya virus and control measures: The case of Italy. *PLOS ONE* **6**, e18860 (2011).

72. M. Robinson, A. Conan, V. Duong, S. Ly, C. Ngan, P. Buchy, A. Tarantola, X. Rodó, A model for a chikungunya outbreak in a rural cambodian setting: Implications for disease control in uninfected areas. *PLOS Negl. Trop. Dis.* **8**, e3120 (2014).

73. A. Whiteman, J. R. Loaiza, D. A. Yee, K. C. Poh, A. S. Watkins, K. J. Lucas, T. J. Rapp, L. Kline, A. Ahmed, S. Chen, E. Delmelle, J. U. Oguzie, Do socioeconomic factors drive *Aedes* mosquito vectors and their arboviral diseases? A systematic review of dengue, chikungunya, yellow fever, and Zika Virus. *One Health* **11**, 100188 (2020).

74. J. H. Huber, M. L. Childs, J. M. Caldwell, E. A. Mordecai, Seasonal temperature variation influences climate suitability for dengue, chikungunya, and Zika transmission. *PLOS Negl. Trop. Dis.* **12**, e0006451 (2018).

75. P. N. Perez-Guzman, L. C. J. Alcantara, U. Obolski, M. M. de Lima, E. A. Ashley, F. Smithuis, P. Horby, R. J. Maude, Z. Lin, A. M. M. Kyaw, J. Lourenço, Measuring mosquito-borne viral suitability in Myanmar and implications for local Zika virus transmission. *PLOS Curr.* **10**, 10.1371/currents.outbreaks.7a6c64436a3085ebba37e5329ba169e6 (2018).

76. T. Le Viet, M. Choisy, J. E. Bryant, D. V. Trong, T. P. Quang, P. Horby, H. N. Tran, H. T. T. Kieu, T. N. Vu, K. N. Van, M. L. Quynh, H. F. L. Wertheim, A dengue outbreak on a floating village at Cat Ba Island in Vietnam. *BMC Public Health* **15**, 940 (2015).

77. A. Burgueño, V. Fonseca, N. Morel, M. Lima, E. Castro, N. R. Guimarães, F. C. M. Iani, V. Bormida, M. N. Cortinas, V. Ramas, L. Coppola, A. I. Bento, L. Franco, J. M. Rico, J. Lourenço, Luiz Carlos Junior Alcantara, H. Chiparelli, Genomic and eco-epidemiological investigations in Uruguay reveal local Chikungunya virus transmission dynamics during its expansion across the Americas in 2023. medRxiv 23294156 [Preprint] (2023). https://doi.org/10.1101/2023.08.17.23294156.

78. R. Lowe, A. Gasparrini, C. J. Van Meerbeeck, C. A. Lippi, R. Mahon, A. R. Trotman, L. Rollock, A. Q. J. Hinds, S. J. Ryan, A. M. Stewart-Ibarra, Nonlinear and delayed impacts of climate on dengue risk in Barbados: A modelling study. *PLOS Med.* **15**, e1002613 (2018).

79. M. S. Shocket, S. J. Ryan, E. A. Mordecai, Temperature explains broad patterns of Ross River virus transmission. *eLife* **7**, e37762 (2018).

80. A. Rohani, I. Suzilah, M. Malinda, I. Anuar, I. Mohd Mazlan, M. Salmah Maszaitun, O. Topek, Y. Tanrang, S. C. Ooi, H. Rozilawati, H. L. Lee, Aedes larval population dynamics and risk for dengue epidemics in Malaysia. *Trop. Biomed.* **28**, 237–248 (2011).

81. A. Leibenzon, M. Assaf, Heterogeneity can markedly increase final outbreak size in the SIR model of epidemics. *Phys. Rev. Res.* **6**, L012010 (2024).

82. S. V. Scarpino, G. Petri, On the predictability of infectious disease outbreaks. *Nat. Commun.* **10**, 898 (2019).

83. L. A. White, J. D. Forester, M. E. Craft, Disease outbreak thresholds emerge from interactions between movement behavior, landscape structure, and epidemiology. *Proc. Natl. Acad. Sci. U.S.A.* **115**, 7374–7379 (2018).

84. E. M. Volz, J. C. Miller, A. Galvani, L. A. Meyers, Effects of heterogeneous and clustered contact patterns on infectious disease dynamics. *PLOS Comput. Biol.* **7**, e1002042 (2011).

85. S. T. Stoddard, B. M. Forshey, A. C. Morrison, V. A. Paz-Soldan, G. M. Vazquez-Prokopec, H. Astete, R. C. Reiner Jr., S. Vilcarromero, J. P. Elder, E. S. Halsey, T. J. Kochel, U. Kitron, T. W. Scott, House-to-house human movement drives dengue virus transmission. *Proc. Natl. Acad. Sci. U.S.A.* **110**, 994–999 (2013).

86. J. Li, D. Blakeley, R. J. Smith, The failure of R_0. *Comput. Math. Methods Med.* **2011**, 527610 (2011).

87. S. C. Eneh, O. Uwishema, A. Nazir, E. E. Jurdi, O. F. Olanrewaju, Z. Abbass, M. M. Jolayemi, N. Mina, L. Kseiry, H. Onyeaka, Chikungunya outbreak in Africa: A review of the literature. *Ann. Med. Surg.* **85**, 3545–3552 (2023).

88. E. Viennet, K. Knope, H. Faddy, C. Williams, D. Harley, Assessing the threat of chikungunya virus emergence in Australia. *Commun. Duis. Intell. Q. Rep.* **37**, E136–E143 (2013).

89. GIDEON, Global Infectious Disease and Epidemiology Online Network (2021); https://www.gideononline.com/.

90. B. Dwibedi, J. Sabat, N. Mahapatra, S. K. Kar, A. S. Kerketta, R. K. Hazra, S. K. Parida, N. S. Marai, M. K. Beuria, Rapid spread of Chikungunya virus infection in Orissa: India. *Indian J. Med. Res.* **133**, 316–321 (2011).

91. A. J. Rodriguez-Morales, J. E. Bedoya-Arias, V. Ramírez-Jaramillo, C. P. Montoya-Arias, E. A. Guerrero-Matituy, E. V. Cárdenas-Giraldo, Using geographic information system (GIS) to mapping and assess changes in transmission patterns of chikungunya fever in municipalities of the Coffee-Triangle region of Colombia during 2014–2015 outbreak: Implications for travel advice. *Travel Med. Infect Dis.* **14**, 62–65 (2016).

92. A. J. Rodriguez-Morales, E. V. Cárdenas-Giraldo, C. P. Montoya-Arias, E. A. Guerrero-Matituy, J. E. Bedoya-Arias, V. Ramírez-Jaramillo, W. E. Villamil-Gómez, Mapping chikungunya fever in municipalities of one coastal department of Colombia (Sucre) using geographic information system (GIS) during 2014 outbreak: Implications for travel advice. *Travel Med. Infect Dis.* **13**, 256–258 (2015).

93. L. I. Zambrano, M. Sierra, B. Lara, I. Rodríguez-Núñez, M. T. Medina, C. O. Lozada-Riascos, A. J. Rodríguez-Morales, Estimating and mapping the incidence of dengue and chikungunya in Honduras during 2015 using Geographic Information Systems (GIS). *J. Infect. Public Health* **10**, 446–456 (2017).

94. J. Bezanson, A. Edelman, S. Karpinski, V. B. Shah, Julia: A fresh approach to numerical computing. *SIAM Rev.* **59**, 65–98 (2017).

95. Esri, ArcGIS Online (2022); https://www.arcgis.com/home/index.html.

96. World Bank Group, Population Estimates and Projections, 1959–2049 (2023); https://databank.worldbank.org/source/population-estimates-and-projections.

97. M. Bondarenko, D. Kerr, A. Sorichetta, A. Tatem, Census/projection-disaggregated gridded population datasets, adjusted to match the corresponding UNPD 2020 estimates, for 183

countries in 2020 using Built-Settlement Growth Model (BSGM) outputs (2020); http://eprints.soton.ac.uk/444005/.

98. R. J. Oidtman, G. España, T. A. Perkins, Co-circulation and misdiagnosis led to underestimation of the 2015–2017 Zika epidemic in the Americas. *PLOS Negl. Trop. Dis.* **15**, e0009208 (2021).

99. L. Godaert, S. Bartholet, Y. Gazeuse, Y. Brouste, F. Najioullah, L. Kanagaratnam, R. Césaire, J.-L. Fanon, M. Dramé, Misdiagnosis of chikungunya virus infection: Comparison of old and younger adults. *J. Am. Geriat. Soc.* **66**, 1768–1772 (2018).

100. S. M. Cavany, G. España, G. M. Vazquez-Prokopec, T. W. Scott, T. A. Perkins, Pandemic-associated mobility restrictions could cause increases in dengue virus transmission. *PLOS Negl. Trop. Dis.* **15**, e0009603 (2021).

101. K. Cranmer, J. Brehmer, G. Louppe, The frontier of simulation-based inference. *Proc. Natl. Acad. Sci. U.S.A.* **117**, 30055–30062 (2020).

102. A. D. Blaom, F. Kiraly, T. Lienart, Y. Simillides, D. Arenas, S. Vollmer, MLJ: A Julia package for composable machine learning. *J. Open Source Softw.* **5**, 2704 (2020).

103. T. A. Perkins, J. H. Huber, Q. M. Tran, R. J. Oidtman, M. K. Walters, A. S. Siraj, S. M. Moore, Burden is in the eye of the beholder: Sensitivity of yellow fever disease burden estimates to modeling assumptions. *Sci. Adv.* **7**, eabg5033 (2021).

104. I. M. Sobol', Global sensitivity indices for nonlinear mathematical models and their Monte Carlo estimates. *Math. Comput. Simul.* **55**, 271–280 (2001).

105. V. K. Dixit, C. Rackauckas, GlobalSensitivity.jl: Performant and parallel globalsensitivity analysis with Julia. *J. Open Source Softw.* **7**, 4561 (2022).

106. M. Woolhouse, C. Dye, J. F. Etard, T. Smith, J. D. Charlwood, G. P. Garnett, P. Hagan, J. L. Hii, P. D. Ndhlovu, R. J. Quinnell, C. H. Watts, S. K. Chandiwana, R. M. Anderson, Heterogeneities in the transmission of infectious agents: Implications for the design of control programs. *Proc. Natl. Acad. Sci. U.S.A.* **94**, 338–342 (1997).

107. M. P. Mammen Jr., C. Pimgate, C. J. M. Koenraadt, A. L. Rothman, J. Aldstadt, A. Nisalak, R. G. Jarman, J. W. Jones, A. Srikiatkhachorn, C. A. Ypil-Butac, A. Getis, S. Thammapalo, A. C. Morrison, D. H. Libraty, S. Green, T. W. Scott, Spatial and temporal clustering of dengue virus transmission in Thai villages. *PLOS Med.* **5**, e205 (2008).

108. G. M. Vazquez-Prokopec, U. Kitron, B. Montgomery, P. Horne, S. A. Ritchie, Quantifying the spatial dimension of dengue virus epidemic spread within a tropical urban environment. *PLOS Negl. Trop. Dis.* **4**, e920 (2010).

109. G. Guzzetta, C. A. Marques-Toledo, R. Rosà, M. Teixeira, S. Merler, Quantifying the spatial spread of dengue in a non-endemic Brazilian metropolis via transmission chain reconstruction. *Nat. Commun.* **9**, 2837 (2018).

110. I. M. Berry, M. C. Melendrez, S. Pollett, K. Figueroa, D. Buddhari, C. Klungthong, A. Nisalak, M. Panciera, B. Thaisomboonsuk, T. Li, T. G. Vallard, L. Macareo, I. K. Yoon, S. J. Thomas, T. Endy, R. G. Jarman, Precision tracing of household dengue spread using inter- and intra-host viral variation data, Kamphaeng Phet, Thailand. *Emerg. Infect. Dis.* **27**, 1637–1644 (2021).

111. M. Casas-Martínez, R. Tamayo-Domínguez, J. G. Bond-Compeán, J. C. Rojas, M. Weber, A. Ulloa-García, Oogenic development and gonotrophic cycle of *Aedes aegypti* and Aedes albopictus in laboratory. *Salud Publica Mex.* **62**, 372–378 (2020).

112. G. Rezza, S. C. Weaver, Chikungunya as a paradigm for emerging viral diseases: Evaluating disease impact and hurdles to vaccine development. *PLOS Negl. Trop. Dis.* **13**, e0006919 (2019).

113. L. A. Silva, T. S. Dermody, Chikungunya virus: Epidemiology, replication, disease mechanisms, and prospective intervention strategies. *J. Clin. Invest.* **127**, 737–749 (2017).

114. J. T. Abatzoglou, S. Z. Dobrowski, S. A. Parks, K. C. Hegewisch, TerraClimate, a high-resolution global dataset of monthly climate and climatic water balance from 1958-2015. *Sci. Data* **5**, 170191 (2018).

115. B. Davenport, What is the standard of living in St. Maarten? (2021); https://www.sint-maarten.net/population/life.

116. B. Cassella, A risk assessment of the United States' Island Territories, 2008-2020 (2020); https://sites.tufts.edu/gis/files/2020/08/cassella_bryan_DHPP207_Spring2020.pdf.

117. Google Maps, Maps of chikungunya-affected populations (2021); https://www.google.com/maps.

118. A. Andrew, T. N. Navien, T. S. Yeoh, M. Citartan, E. Mangantig, M. S. H. Sum, E. Seng Ch'ng, T.-H. Tang, Diagnostic accuracy of serological tests for the diagnosis of Chikungunya virus infection: A systematic review and meta-analysis. *PLOS Negl. Trop. Dis.* **16**, e0010152 (2022).

119. B. W. Johnson, B. J. Russell, C. H. Goodman, Laboratory diagnosis of chikungunya virus infections and commercial sources for diagnostic assays. *J. Infect. Dis.* **214**, S471–S474 (2016).

120. T. Edwards, L. Del Carmen Castillo Signor, C. Williams, C. Larcher, M. Espinel, J. Theaker, E. Donis, L. E. Cuevas, E. R. Adams, Analytical and clinical performance of a Chikungunya qRT-PCR for Central and South America. *Diagn. Microbiol. Infect. Dis.* **89**, 35–39 (2017).

121. P. Grivard, K. Le Roux, P. Laurent, A. Fianu, J. Perrau, J. Gigan, G. Hoarau, N. Grondin, F. Staikowsky, F. Favier, A. Michault, Molecular and serological diagnosis of Chikungunya virus infection. *Pathol. Biol.* **55**, 490–494 (2007).

122. S. Khatun, A. Chakraborty, M. Rahman, N. N. Banu, M. M. Rahman, S. M. Murshid Hasan, S. P. Luby, E. S. Gurley, An outbreak of chikungunya in rural Bangladesh, 2011. *PLOS Negl. Trop. Dis.* **9**, e0003907 (2015).

123. A. Kumar, C. Best, G. Benskin, Epidemiology, clinical and laboratory features and course of chikungunya among a cohort of children during the first caribbean epidemic. *J. Trop. Pediatr.* **63**, 43–49 (2017).

124. S. Wangchuk, P. Chinnawirotpisan, T. Dorji, T. Tobgay, T. Dorji, I. K. Yoon, S. Fernandez, Chikungunya fever outbreak, Bhutan, 2012. *Emerg. Infect. Dis.* **19**, 1681–1684 (2013).

125. J. P. Dias, M. C. N. Costa, G. S. Campos, E. S. Paixão, M. S. Natividade, F. R. Barreto, M. S. C. Itaparica, C. Goes, F. L. S. Oliveira, E. B. Santana, N. S. J. Silva, C. A. A. Brito, L. C. Rodrigues, S. I. Sardi, R. C. Saavedra, M. G. Teixeira, Seroprevalence of Chikungunya virus after its emergence in Brazil. *Emerg. Infect. Dis.* **24**, 617–624 (2018).

126. Centers for Disease Control and Prevention (CDC), Chikungunya Outbreak – Cambodia, February–March 2012. *MMWR Morb. Mortal. Wkly. Rep.* **61**, 737–740 (2012).

127. S. Ahmed, L. Francis, R. P. Ricketts, T. Christian, K. Polson-Edwards, B. Olowokure, Chikungunya virus outbreak, Dominica, 2014. *Emerg. Infect. Dis.* **21**, 909–911 (2015).

128. R. M. Langsjoen, R. J. Rubinstein, T. F. Kautz, A. J. Auguste, J. H. Erasmus, L. Kiaty-Figueroa, R. Gerhardt, D. Lin, K. L. Hari, R. Jain, N. Ruiz, A. E. Muruato, J. Silfa, F. Bido, M. Dacso, S. C. Weaver, Molecular virologic and clinical characteristics of a Chikungunya Fever outbreak in La Romana, Dominican republic, 2014. *PLOS Negl. Trop. Dis.* **10**, e0005189 (2016).

129. R. Pimentel, R. Skewes-Ramm, J. Moya, Chikungunya in the Dominican Republic: Lessons learned in the first six months. *Rev. Panam. Salud. Publica.* **36**, 336–341 (2014).

130. M. Caron, C. Paupy, G. Grard, P. Becquart, I. Mombo, B. B. B. Nso, F. Kassa Kassa, D. Nkoghe, E. M. Leroy, Recent introduction and rapid dissemination of chikungunya virus and dengue virus serotype 2 associated with human and mosquito coinfections in Gabon, Central Africa. *Clin. Infect. Dis.* **55**, e45 (2012).

131. C. Macpherson, T. Noël, P. Fields, D. Jungkind, K. Yearwood, M. Simmons, S. Widjaja, G. Mitchell, D. Noel, S. Bidaisee, T. E. Myers, A. D. LaBeaud, Clinical and serological insights from the Asian lineage Chikungunya outbreak in Grenada, 2014: An observational study. *Am. J. Trop. Med. Hyg.* **95**, 890–893 (2016).

132. C. K. Uthappa, R. R. Allam, D. Gunti, C. Nalini, P. R. Udaragudi, G. P. Tadi, M. V. Murhekar, Chikungunya outbreak in Atmakur village, Medak district, Telangana State, India. *Indian J. Med. Res.* **142**, S108–S110 (2015).

133. A. Chopra, V. Anuradha, R. Ghorpade, M. Saluja, Acute Chikungunya and persistent musculoskeletal pain following the 2006 Indian epidemic: A 2-year prospective rural community study. *Epidemiol. Infect.* **140**, 842 (2012).

134. P. Kaur, M. Ponniah, M. V. Murhekar, V. Ramachandran, R. Ramachandran, H. K. Raju, V. Perumal, A. C. Mishra, M. D. Gupte, Chikungunya outbreak, South India, 2006. *Emerg. Infect. Dis.* **14**, 1623–1625 (2008).

135. S. A. Khan, P. Dutta, R. Topno, J. Borah, P. Chowdhury, J. Mahanta, Chikungunya outbreak in Garo Hills, Meghalaya: An epidemiological perspective. *Indian J. Med. Res.* **141**, 591–597 (2015).

136. K. Sari, K. S. A. Myint, A. R. Andayani, P. D. Adi, R. Dhenni, A. Perkasa, C. N. Ma'roef, N. P. D. Witari, D. Megawati, A. M. Powers, U. A. Jaya, Chikungunya fever outbreak identified in North Bali, Indonesia. *Trans. R. Soc. Trop. Med. Hyg.* **111**, 325–327 (2017).

137. F. Y. Sitepu, E. Depari, Epidemiological and entomological investigation of Chikungunya fever outbreak, in Serdang Bedagai District, North Sumatera Province, Indonesia, 2013. *Global Biosec.* **1**, (2019).

138. F. Y. Sitepu, A. Suprayogi, D. Pramono, H. Harapan, M. Mudatsir, Epidemiological investigation of chikungunya outbreak, West Kalimantan, Indonesia. *Clin. Epidemiol. Global Health* **8**, 113–116 (2020).

139. F. Riccardo, G. Venturi, M. di Luca, M. del Manso, F. Severini, X. Andrianou, C. Fortuna, M. E. Remoli, E. Benedetti, M. G. Caporali, F. Fratto, A. D. Mignuoli, L. Rizzo, G. de Vito, V. de Giorgio, L. Surace, F. Vairo, P. Angelini, M. C. Re, A. Amendola, C. Fiorentini, G. Marsili, L. Toma, D. Boccolini, R. Romi, P. Pezzotti, G. Rezza, C. Rizzo, Secondary autochthonous outbreak of Chikungunya, Southern Italy, 2017. *Emerg. Infect. Dis.* **25**, 2093–2095 (2019).

140. S. Somlor, K. Vongpayloth, L. Diancourt, P. Buchy, V. Duong, D. Phonekeo, P. Ketmayoon, P. Vongphrachanh, P. T. Brey, V. Caro, Y. Buisson, M. Grandadam, Chikungunya virus emergence in the Lao PDR, 2012–2013. *PLOS ONE* **12**, e0189879 (2017).

141. O. Noridah, V. Paranthaman, S. K. Nayar, M. Masliza, K. Ranjit, I. Norizah, Y. K. Chem, B. Mustafa, V. Kumarasamy, K. B. Chua, Outbreak of Chikungunya due to virus of Central/East African genotype in Malaysia. *Med. J. Malaysia* **62**, 6 (2007).

142. S. K. Lam, K. B. Chua, P. S. Hooi, M. A. Rahimah, S. Kumari, M. Tharmaratnam, S. K. Chuah, D. W. Smith, I. A. Sampson, Chikungunya infection—An emerging disease in Malaysia. *Southeast Asian J. Trop. Med. Public Health* **32**, 447–451 (2001).

143. J. E. Ballera, M. J. Zapanta, V. C. de los Reyes, M. N. Sucaldito, E. Tayag, Investigation of chikungunya fever outbreak in Laguna, Philippines, 2012. *Western Pac. Surveill. Response J.* **6**, 3 (2015).

144. L.-C. Ng, L. K. Tan, C. H. Tan, S. S. Y. Tan, H. C. Hapuarachchi, K. Y. Pok, Y. L. Lai, S. G. Lam-Phua, G. Bucht, R. T. P. Lin, Y. S. Leo, B. H. Tan, H. K. Han, P. L. S. Ooi, L. James, S. P. Khoo, Entomologic and virologic investigation of Chikungunya, Singapore. *Emerg. Infect. Dis.* **15**, 1243–1249 (2009).

145. S. A. M. Kularatne, S. C. Weerasinghe, C. Gihan, S. Wickramasinghe, S. Dharmarathne, A. Abeyrathna, T. Jayalath, Epidemiology, clinical manifestations, and long-term outcomes of a major outbreak of Chikungunya in a Hamlet in Sri Lanka, in 2007: A longitudinal cohort study. *J. Trop. Med.* **2012**, e639178 (2012).

146. N. Gay, D. Rousset, P. Huc, S. Matheus, M. Ledrans, J. Rosine, S. Cassadou, H. Noël, Seroprevalence of Asian lineage chikungunya virus infection on Saint Martin Island, 7 months after the 2013 emergence. *Am. J. Trop. Med. Hyg.* **94**, 393–396 (2016).

147. M. Henry, L. Francis, V. Asin, K. Polson-Edwards, B. Olowokure, Chikungunya virus outbreak in Sint Maarten, 2013–2014. *Rev. Panam. Salud Pública* **41**, e61 (2017).

148. S. Thanajirasak, N. Hoomhual, S. Poonkesorn, S. Rangsriwong, S. Poungsombat, An investigation of chikungunya outbreak in Satun, May–September 2018. *Wkly. Epidemiol. Surveill. Rep.* **50**, 505 (2019).

149. L. R. Feldstein, E. M. Ellis, A. Rowhani-Rahbar, M. E. Halloran, B. R. Ellis, The first reported outbreak of Chikungunya in the U.S. Virgin Islands, 2014–2015. *Am. J. Trop. Med. Hyg.* **95**, 885–889 (2016).

150. S. M. Moore, R. J. Oidtman, K. J. Soda, A. S. Siraj, R. C. Reiner Jr., C. M. Barker, T. A. Perkins, Leveraging multiple data types to estimate the size of the Zika epidemic in the Americas. *PLOS Negl. Trop. Dis.* **14**, e0008640 (2020).

151. G. Guzzetta, F. Vairo, A. Mammone, S. Lanini, P. Poletti, M. Manica, R. Rosa, B. Caputo, A. Solimini, A. D. Torre, P. Scognamiglio, A. Zumla, G. Ippolito, S. Merler, Spatial modes for transmission of chikungunya virus during a large chikungunya outbreak in Italy: A modeling analysis. *BMC Med.* **18**, 226 (2020).

152. H. Salje, H. Salje, A. Wesolowski, T. S. Brown, M. V. Kiang, I. M. Berry, N. Lefrancq, S. Fernandez, R. G. Jarman, K. Ruchusatsawat, S. Iamsirithaworn, W. P. Vandepitte, P. Suntarattiwong, J. M. Read, C. Klungthong, B. Thaisomboonsuk, K. Engø-Monsen, C. Buckee, S. Cauchemez, D. A. T. Cummings, Reconstructing unseen transmission events to infer dengue dynamics from viral sequences. *Nat. Commun.* **12**, 1810 (2021).

153. A. Vega-Rúa, K. Zouache, R. Girod, A.-B. Failloux, R. Lourenço-de Oliveira, High level of vector competence of *Aedes aegypti* and *Aedes albopictus* from ten American countries as a crucial factor in the spread of chikungunya virus. *J. Virol.* **88**, 6294–6306 (2014).

154. A. Vega-Rúa, R. Lourenço-de-Oliveira, L. Mousson, M. Vazeille, S. Fuchs, A. Yébakima, J. Gustave, R. Girod, I. Dusfour, I. Leparc-Goffart, D. L. Vanlandingham, Y. J. S. Huang, L. P. Lounibos, S. Mohamed Ali, A. Nougairede, X. de Lamballerie, A. B. Failloux, Chikungunya virus transmission potential by local Aedes mosquitoes in the Americas and Europe. *PLOS Negl. Trop. Dis.* **9**, e0003780 (2015).

155. L. I.-K. Lin, A concordance correlation coefficient to evaluate reproducibility. *Biometrics* **45**, 255 (1989).

156. Y. Sun, M. G. Genton, Functional boxplots. *J. Comput. Graph. Stat.* **20**, 316 (2011).
